# Supplementary material for: ON selectivity in the Drosophila visual system is a multisynaptic process involving both glutamatergic and GABAergic inhibition
Source: eLife. 2019 Sep 19;8:e49373. doi: 10.7554/eLife.49373 (PMC6845231; doi:10.7554/eLife.49373)
Supplement: Figure 2—source data 1. — Data related to quantifications shown in main Figure 2, sorted by genotype and experimental condition. [file elife-49373-fig2-data1.docx]

**Figure 2 – source data 1:** Table 1 contains all mean ± s.e.m. data related to quantifications shown in main Figure 2, sorted by genotype and experimental condition.

**Table 1**

| **Figure 2 B,D** |  |  |  |  |
| --- | --- | --- | --- | --- |
|  | **ON Step Layer M9/10** | | | |
|  | **sham** | **1μM PTX** | **2.5μM PTX** | **5μM PTX** |
| **Mi1 >> GCaMP6f** | 0.746 ± 0.067 | 0.439 ± 0.133 | 0.032 ± 0.002 | -0.016 ± 0.008 |
| **Tm3 >> GaMP6f** | 0.951 ± 0.136 | 0.418 ± 0.095 | 0.190 ± 0.020 | 0.328 ± 0.094 |
|  |  | **25μM PTX** | **50μM PTX** | **100μM PTX** |
| **Mi1 >> GCaMP6f** |  | -0.043 ± 0.027 | -0.029 ± 0.011 | -0.023 ± 0.007 |
| **Tm3 >> GaMP6f** |  | -0.061 ± 0.015 | -0.057 ± 0.007 | -0.102 ± 0.012 |

| **Figure 2 F** | | |  | |  | |  | |
| --- | --- | --- | --- | --- | --- | --- | --- | --- |
|  | | | **ON Step Axon terminals** | | | | | |
|  | | | **0μM PTX** | | **2.5μM PTX** | | **100μM PTX** | |
| **T4/T5 >> GCaMP6f** | | | 0.161 ± 0.038 | | 1.697± 0.199, | | -0.073 ± 0.020 | |
|  | | |  | |  | |  | |
|  | | | **OFF Step Axon terminals** | | | | | |
|  | | | **0μM PTX** | | **2.5μM PTX** | | **100μM PTX** | |
| **T4/T5 >> GCaMP6f** | | | 0.212 ± 0.060 | | 1.719 ± 0.139 | | 1.373 ± 0.440 | |
|  | | |  | |  | |  | |
| **Figure 2 H** |  | |  | |  | |  | |
|  | **ON Step Layer M1** | | | | **ON Step Layer M5** | | | |
|  | **0μM PTX** | | **100μM PTX** | | **0μM PTX** | | **100μM PTX** | |
| **L1 >> GCaMP6f** | -0.441 ± 0.057 | | -0.643 ± 0.015 | | -0.210 ± 0.039 | | -0.458 ± 0.035 | |
|  |  | |  | |  | |  | |
|  | **OFF Step Layer M1** | | | | **OFF Step Layer M5** | | | |
|  | **0μM PTX** | | **100μM PTX** | | **0μM PTX** | | **100μM PTX** | |
| **L1 >> GCaMP6f** | 0.496 ± 0.082 | | 0.432± 0.045 | | 0.176 ± 0.040 | | 0.169 ± 0.021 | |
