# Supplementary material for: ON selectivity in the Drosophila visual system is a multisynaptic process involving both glutamatergic and GABAergic inhibition
Source: eLife. 2019 Sep 19;8:e49373. doi: 10.7554/eLife.49373 (PMC6845231; doi:10.7554/eLife.49373)
Supplement: Figure 2—figure supplement 1—source data 1. — Data related to quantifications shown in main Figure 2—figure supplement 1, sorted by genotype and experimental condition. [file elife-49373-fig2-figsupp1-data1.docx]

**Figure 2-figure supplement 1 – source data 1:** Table 1 contains all mean ± s.e.m. data related to quantifications shown in main Figure 2-figure supplement 1, sorted by genotype and experimental condition.

**Table 1**

| **Figure S1 B,D** |  |  |
| --- | --- | --- |
|  | **ON Step LAYER M9/10** | |
|  | **0μM MPEP** | **100μM MPEP** |
| **Mi1 >> GCaMP6f** | 1.000± 0.000 | 1.147 ± 0.033 |
| **Tm3 >> GaMP6f** | 1.000± 0.000 | 0.819 ± 0.164 |
