# Supplementary material for: ON selectivity in the Drosophila visual system is a multisynaptic process involving both glutamatergic and GABAergic inhibition
Source: eLife. 2019 Sep 19;8:e49373. doi: 10.7554/eLife.49373 (PMC6845231; doi:10.7554/eLife.49373)
Supplement: Figure 2—figure supplement 2—source data 1. — Data related to quantifications shown in main Figure 2—figure supplement 2, sorted by genotype and experimental condition. [file elife-49373-fig2-figsupp2-data1.docx]

**Figure 2-figure supplement 2 – source data 1:** Table 1 contains all mean ± s.e.m. data related to quantifications shown in main Figure 2-figure supplement 2, sorted by genotype and experimental condition.

**Table 1**

| **Figure S2 C,D** |  |  |  |  |
| --- | --- | --- | --- | --- |
|  | **ON Step Layer M1** | | | |
|  | **sham** | **1μM PTX** | **5μM PTX** | **100μM PTX** |
| **Mi1 >> GCaMP6f** | 0.740 ± 0.069 | 0.445 ± 0.125 | 0.072 ± 0.020 | -0.170 ± 0.039 |
| **Tm3 >> GaMP6f** | 0.991 ± 0.277 | 0.589 ± 0.150 | 0.208 ± 0.035 | -0.187 ± 0.026 |
|  |  |  |  |  |
|  | **ON Step Layer M5** | | | |
|  | **sham** | **1μM PTX** | **5μM PTX** | **100μM PTX** |
| **Mi1 >> GCaMP6f** | 0.649 ± 0.050 | 0.450 ± 0.133 | 0.046 ± 0.003 | -0.147 ± 0.038 |
| **Tm3 >> GaMP6f** | 0.942 ± 0.168 | 0.523 ± 0.158 | 0.217 ± 0.037 | -0.192 ± 0.031 |
