# Supplementary material for: ON selectivity in the Drosophila visual system is a multisynaptic process involving both glutamatergic and GABAergic inhibition
Source: eLife. 2019 Sep 19;8:e49373. doi: 10.7554/eLife.49373 (PMC6845231; doi:10.7554/eLife.49373)
Supplement: Figure 2—figure supplement 3—source data 1. — Data related to quantifications shown in main Figure 2—figure supplement 3, sorted by genotype and experimental condition. [file elife-49373-fig2-figsupp3-data1.docx]

**Figure 2-figure supplement 3 – source data 1:** Table 1 contains all mean ± s.e.m. data related to quantifications shown in main Figure 2-figure supplement 3, sorted by genotype and experimental condition.

**Table 1**

| **Figure S3 A** |  |  |  |  |
| --- | --- | --- | --- | --- |
|  | **ON Step** | | | |
|  | **layer M1** | | **layer M5** | |
|  | **0 μM** | **5 μM** | **0 μM** | **5 μM** |
| **Mi1 >> iGluSnFR** | -0.092 ± 0.007 | -0.173 ± 0.025 | -0.044 ± 0.003 | -0.144 ± 0.031 |
|  | **0 μM** | **100 μM** | **0 μM** | **100 μM** |
| **Mi1 >> iGluSnFR** | -0.113 ± 0.010 | -0.289 ± 0.005 | -0.056 ± 0.008 | -0.229 ± 0.013 |
|  |  |  |  |  |
|  | **OFF Step** | | | |
|  | **layer M1** | | **layer M5** | |
|  | **0 μM** | **5 μM** | **0 μM** | **5 μM** |
| **Mi1 >> iGluSnFR** | 0.181 ± 0.018 | 0.200 ± 0.018 | 0.070 ± 0.004 | 0.097± 0.016 |
|  | **0 μM** | **100 μM** | **0 μM** | **100 μM** |
| **Mi1 >> iGluSnFR** | 0.194 ± 0.025 | 0.283± 0.046 | 0.081 ± 0.008 | 0.194 ± 0.013 |
|  |  |  |  |  |
|  |  |  |  |  |
| **Figure S3 B** |  |  |  |  |
|  | **ON Step** | | | |
|  | **layer M1** | | **layer M5** | |
|  | **0 μM** | **5 μM** | **0 μM** | **5 μM** |
| **Tm3 >> iGluSnFR** | -0.161 ± 0.002 | -0.174 ± 0.014 | -0.068 ± 0.009 | -0.099 ± 0.024 |
|  | **0 μM** | **100 μM** | **0 μM** | **100 μM** |
| **Tm3 >> iGluSnFR** | -0.181 ± 0.006 | -0.285± 0.019 | -0.083 ± 0.011 | -0.233 ± 0.032 |
|  |  |  |  |  |
|  | **OFF Step** | | | |
|  | **layer M1** | | **layer M5** | |
|  | **0 μM** | **5 μM** | **0 μM** | **5 μM** |
| **Tm3 >> iGluSnFR** | 0.275 ± 0.033 | 0.271 ± 0.018 | 0.144 ± 0.005 | 0.125 ± 0.007 |
|  | **0 μM** | **100 μM** | **0 μM** | **100 μM** |
| **Tm3 >> iGluSnFR** | 0.217 ± 0.036 | 0.256 ± 0.022 | 0.139 ± 0.013 | 0.209 ± 0.013 |
