# Supplementary material for: ON selectivity in the Drosophila visual system is a multisynaptic process involving both glutamatergic and GABAergic inhibition
Source: eLife. 2019 Sep 19;8:e49373. doi: 10.7554/eLife.49373 (PMC6845231; doi:10.7554/eLife.49373)
Supplement: Figure 4—source data 1. — Data related to quantifications shown in main Figure 4, sorted by genotype and experimental condition. [file elife-49373-fig4-data1.docx]

**Figure 4 – source data 1:** Table 1 contains all mean ± s.e.m. data related to quantifications shown in main Figure 4, sorted by genotype and experimental condition.

**Table 1**

| **Figure 4 C,F** |  |  |  |  |
| --- | --- | --- | --- | --- |
|  | **Ctrl Layer M1** | | **MDRR Layer M1** | |
|  | **ON Step** | | **ON Step** | |
|  | **0μM PTX** | **2.5μM PTX** | **0μM PTX** | **2.5μM PTX** |
| **Mi1 >> GCaMP6f** | 1.000 ± 0.000 | 0 .300 ± 0.051 | 1.000 ± 0.000 | 0.710 ± 0.114 |
| **Tm3 >> GaMP6f** | 1.000 ± 0.000 | 0.356 ± 0.105 | 1.000 ± 0.000 | 0.948 ± 0.106 |
|  |  |  |  |  |
|  | **ON Plateau** | | **ON Plateau** | |
|  | **0μM PTX** | **2.5μM PTX** | **0μM PTX** | **2.5μM PTX** |
| **Mi1 >> GCaMP6f** | 0.234± 0.049 | -0.050± 0.031 | 0.238 ± 0.072 | 0.169 ± 0.055 |
| **Tm3 >> GaMP6f** | 0.020 ± 0.006 | 0.035 ± 0.001 | 0.068 ± 0.020 | 0.165 ± 0.036 |
|  |  |  |  |  |
|  | **ON Integral** | | **ON Integral** | |
|  | **0μM PTX** | **2.5μM PTX** | **0μM PTX** | **2.5μM PTX** |
| **Mi1 >> GCaMP6f** | 1.000 ± 0.000 | -0.247 ± 0.150 | 1.000 ± 0.000 | 0.586 ± 0.151 |
| **Tm3 >> GaMP6f** | 1.000 ± 0.000 | 0.383 ± 0.232 | 1.000 ± 0.000 | 1.286 ± 0.230 |
