# Supplementary material for: ON selectivity in the Drosophila visual system is a multisynaptic process involving both glutamatergic and GABAergic inhibition
Source: eLife. 2019 Sep 19;8:e49373. doi: 10.7554/eLife.49373 (PMC6845231; doi:10.7554/eLife.49373)
Supplement: Figure 4—figure supplement 1—source data 1. — Data related to quantifications shown in main Figure 4—figure supplement 1, sorted by genotype and experimental condition. [file elife-49373-fig4-figsupp1-data1.docx]

**Figure 4-figure supplement 1 – source data 1:** Table 1 contains all mean ± s.e.m. data related to quantifications shown in main Figure 4-figure supplement 1, sorted by genotype and experimental condition.

**Table 1**

| **Figure S5 D,G** |  |  |  |  |
| --- | --- | --- | --- | --- |
|  | **Ctrl Layer M5** | | **MDRR Layer M5** | |
|  | **ON Step** | | **ON Step** | |
|  | **0μM PTX** | **2.5μM PTX** | **0μM PTX** | **2.5μM PTX** |
| **Mi1 >> GCaMP6f** | 1.000± 0.000 | 0.486± 0.077 | 1.000± 0.000 | 0.617± 0.085 |
| **Tm3 >> GaMP6f** | 1.000± 0.000 | 0.471 ± 0.195 | 1.000± 0.000 | 0.795 ± 0.084 |
|  |  |  |  |  |
|  | **ON Plateau** | | **ON Plateau** | |
|  | **0μM PTX** | **2.5μM PTX** | **0μM PTX** | **2.5μM PTX** |
| **Mi1 >> GCaMP6f** | 0.289 ± 0.037 | -0.006 ± 0.040 | 0.253 ± 0.062 | 0.172 ± 0.053 |
| **Tm3 >> GaMP6f** | 0.308 ± 0.105 | 0.187 ± 0.098 | 0.358 ± 0.060 | 0.281 ± 0.043 |
|  |  |  |  |  |
|  | **ON Integral** | | **ON Integral** | |
|  | **0μM PTX** | **2.5μM** | **0μM PTX** | **2.5μM PTX** |
| **Mi1 >> GCaMP6f** | 1.000± 0.000 | 0.131 ± 0.067 | 1.000± 0.000 | 0.565 ± 0.242 |
| **Tm3 >> GaMP6f** | 1.000± 0.000 | 0.463 ± 0.249 | 1.000± 0.000 | 0.871 ± 0.149 |
|  |  |  |  |  |
|  |  |  |  |  |
|  | **Ctrl Layer M9/10** | | **MDRR Layer M9/10** | |
|  | **ON Step** | | **ON Step** | |
|  | **0μM PTX** | **2.5μM PTX** | **0μM PTX** | **2.5μM PTX** |
| **Mi1 >> GCaMP6f** | 1.000± 0.000 | 0.317± 0.070 | 1.000± 0.000 | 0.483 ± 0.102 |
| **Tm3 >> GaMP6f** | 1.000± 0.000 | 0.333 ± 0.127 | 1.000± 0.000 | 0.628 ± 0.134 |
|  |  |  |  |  |
|  | **ON Plateau** | | **ON Plateau** | |
|  | **0μM PTX** | **2.5μM PTX** | **0μM PTX** | **2.5μM PTX** |
| **Mi1 >> GCaMP6f** | 0.272 ± 0.020 | 0.030 ± 0.020 | 0.234 ± 0.055 | 0.156 ± 0.049, |
| **Tm3 >> GaMP6f** | 0.070 ± 0.004 | 0.025 ± 0.011 | 0.127 ± 0.001 | 0.123 ± 0.031 |
|  |  |  |  |  |
|  | **ON Integral** | | **ON Integral** | |
|  | **0μM PTX** | **2.5μM PTX** | **0μM PTX** | **2.5μM PTX** |
| **Mi1 >> GCaMP6f** | 1.000± 0.000 | 0.037 ± 0.082 | 1.000± 0.000 | 0.416 ± 0.117 |
| **Tm3 >> GaMP6f** | 1.000± 0.000 | 0.368± 0.2284 | 1.000± 0.000 | 0.699 ± 0.142 |
