# Supplementary material for: ON selectivity in the Drosophila visual system is a multisynaptic process involving both glutamatergic and GABAergic inhibition
Source: eLife. 2019 Sep 19;8:e49373. doi: 10.7554/eLife.49373 (PMC6845231; doi:10.7554/eLife.49373)
Supplement: Figure 5—source data 1. — Data related to quantifications shown in main Figure 5, sorted by genotype and experimental condition. [file elife-49373-fig5-data1.docx]

**Figure 5 – source data 1:** Table 1 contains all mean ± s.e.m. data related to quantifications shown in main Figure 5, sorted by genotype and experimental condition.

**Table 1**

| **Figure 5 E** |  |  |  |
| --- | --- | --- | --- |
|  | **Peak/Post** | | |
|  | **Basal** | **10μM PTX** | **100μM PTX** |
| **WT** | 1.106 ± 0.109 | 0.805 ± 0.061 | 0.416 ± 0.113 |
| **S278T** | 0.988 ± 0.093 | 1.001 ± 0.064 | 0.884 ± 0.039 |
