# Supplementary material for: ON selectivity in the Drosophila visual system is a multisynaptic process involving both glutamatergic and GABAergic inhibition
Source: eLife. 2019 Sep 19;8:e49373. doi: 10.7554/eLife.49373 (PMC6845231; doi:10.7554/eLife.49373)
Supplement: Figure 6—source data 1. — Data related to quantifications shown in main Figure 6, sorted by genotype and experimental condition. [file elife-49373-fig6-data1.docx]

**Figure 6 – source data 1:** Table 1 contains all mean ± s.e.m. data related to quantifications shown in main Figure 6, sorted by genotype and experimental condition.

**Table 1**

| **Figure 6 C,F** |  |  |  |  |
| --- | --- | --- | --- | --- |
|  | **Ctrl Layer M1** | | **S278T Layer M1** | |
|  | **ON Step** | | **ON Step** | |
|  | **0μM PTX** | **2.5μM PTX** | **0μM PTX** | **2.5μM PTX** |
| **Mi1 >> GCaMP6f** | 1.000 ± 0.000 | 0.140 ± 0.041 | 1.000 ± 0.000 | 0.632 ± 0.104 |
| **Tm3 >> GaMP6f** | 1.000 ± 0.000 | 0.731 ± 0.253 | 1.000 ± 0.000 | 1.086 ± 0.222 |
|  |  |  |  |  |
|  | **ON Plateau** | | **ON Plateau** | |
|  | **0μM PTX** | **2.5μM PTX** | **0μM PTX** | **2.5μM PTX** |
| **Mi1 >> GCaMP6f** | 0.072 ± 0.005 | 0.018 ± 0.007 | 0.288± 0.035 | 0.102 ± 0.031 |
| **Tm3 >> GaMP6f** | 0.055 ± 0.062 | 0.007 ± 0.009 | 0.139 ± 0.024 | 0.071 ± 0.023 |
|  |  |  |  |  |
|  | **ON Integral** | | **ON Integral** | |
|  | **0μM PTX** | **2.5μM PTX** | **0μM PTX** | **2.5μM PTX** |
| **Mi1 >> GCaMP6f** | 1.000 ± 0.000 | 0.178 ± 0.070 | 1.000 ± 0.000 | 0.446 ± 0.199 |
| **Tm3 >> GaMP6f** | 1.000 ± 0.000 | 0.170 ± 0.175 | 1.000 ± 0.000 | 0.765 ± 0.003 |

| **Figure 6 I** |  |  |  |  |
| --- | --- | --- | --- | --- |
|  | **Ctrl Axon terminals** | | **S278T Axon terminals** | |
|  | **ON Step** | | **ON Step** | |
|  | **0μM PTX** | **100μM PTX** | **0μM PTX** | **100μM PTX** |
| **T4/T5 >> GCaMP6f** | 0.122 ± 0.022 | -0.535 ± 0.063 | 0.097± 0.026 | 3.039 ± 0.198 |
|  |  |  |  |  |
|  | **OFF Step** | | **OFF Step** | |
|  | **0μM PTX** | **100μM PTX** | **0μM PTX** | **100μM PTX** |
| **T4/T5 >> GCaMP6f** | 0.186 ± 0.036 | 1.936 ± 0.209 | 0.344/- 0.040 | 1,534 ± 0.189 |
