# Supplementary material for: ON selectivity in the Drosophila visual system is a multisynaptic process involving both glutamatergic and GABAergic inhibition
Source: eLife. 2019 Sep 19;8:e49373. doi: 10.7554/eLife.49373 (PMC6845231; doi:10.7554/eLife.49373)
Supplement: Figure 6—figure supplement 1—source data 1. — Data related to quantifications shown in main Figure 6—figure supplement 1, sorted by genotype and experimental condition. [file elife-49373-fig6-figsupp1-data1.docx]

**Figure 6-figure supplement 1 – source data 1:** Table 1 contains all mean ± s.e.m. data related to quantifications shown in main Figure 6-figure supplement 1, sorted by genotype and experimental condition.

**Table 1**

| **Figure S7 C,F** |  |  |  |  |
| --- | --- | --- | --- | --- |
|  | **Ctrl Layer M5** | | **S278T Layer M5** | |
|  | **ON Step** | | **ON Step** | |
|  | **0μM PTX** | **2.5μM PTX** | **0μM PTX** | **2.5μM PTX** |
| **Mi1 >> GCaMP6f** | 1.000± 0.000 | 0.152 ± 0.042 | 1.000± 0.000 | 0.869 ± 0.212 |
| **Tm3 >> GaMP6f** | 1.000± 0.000 | 0.454 ± 0.147 | 1.000± 0.000 | 0.824 ± 0.169 |
|  |  |  |  |  |
|  | **ON Plateau** | | **ON Plateau** | |
|  | **0μM PTX** | **2.5μM PTX** | **0μM PTX** | **2.5μM PTX** |
| **Mi1 >> GCaMP6f** | 0.116 ± 0.041 | 0.032 ± 0.013 | 0.341 ± 0.045 | 0.112 ± 0.053 |
| **Tm3 >> GaMP6f** | 0.254 ± 0.065 | 0.029 ± 0.036 | 0.429 ± 0.091 | 0.178 ± 0.084 |
|  |  |  |  |  |
|  | **ON Integral** | | **ON Integral** | |
|  | **0μM PTX** | **2.5μM PTX** | **0μM PTX** | **2.5μM PTX** |
| **Mi1 >> GCaMP6f** | 1.000± 0.000 | 0.185 ± 0.067 | 1.000± 0.000 | 0.456 ± 0.153 |
| **Tm3 >> GaMP6f** | 1.000± 0.000 | 0.116 ± 0.127 | 1.000± 0.000 | 0.521 ± 0.229 |
|  |  |  |  |  |
|  |  |  |  |  |
|  | **Ctrl Layer M9/10** | | **S278T Layer M9/10** | |
|  | **ON Step** | | **ON Step** | |
|  | **0μM PTX** | **2.5μM PTX** | **0μM PTX** | **2.5μM PTX** |
| **Mi1 >> GCaMP6f** | 1.000± 0.000 | 0.015 ± 0.006 | 1.000± 0.000 | 0.459 ± 0.101 |
| **Tm3 >> GaMP6f** | 1.000± 0.000 | 0.316 ± 0.181 | 1.000± 0.000 | 0.818 ± 0.156 |
|  |  |  |  |  |
|  | **ON Plateau** | | **ON Plateau** | |
|  | **0μM PTX** | **2.5μM PTX** | **0μM PTX** | **2.5μM PTX** |
| **Mi1 >> GCaMP6f** | 0.277 ± 0.062 | 0.007 ± 0.004 | 0.544 ± 0.051 | 0.171 ± 0.083 |
| **Tm3 >> GaMP6f** | 0.068 ± 0.087 | -0.001 ± 0.029 | 0.249 ± 0.058 | 0.102 ± 0.049 |
|  |  |  |  |  |
|  | **ON Integral** | | **ON Integral** | |
|  | **0μM PTX** | **2.5μM PTX** | **0μM PTX** | **2.5μM PTX** |
| **Mi1 >> GCaMP6f** | 1.000± 0.000 | 0.007 ± 0.005 | 1.000± 0.000 | 0.061 ± 0.035 |
| **Tm3 >> GaMP6f** | 1.000± 0.000 | 0.036 ± 0.038 | 1.000± 0.000 | 0.670 ± 0.115 |

| **Figure S7 I,L** |  |  |  |  |
| --- | --- | --- | --- | --- |
|  | **ON Step Ctrl** | | **ON Step S278T** | |
|  | **Layer M1** | | **Layer M1** | |
|  | **0μM PTX** | **100μM PTX** | **0μM PTX** | **100μM PTX** |
| **Mi1 >> GCaMP6f** | 1.000± 0.000 | -0.121 ± 0.025 | 1.000± 0.000 | -0.085 ± 0.008 |
| **Tm3 >> GaMP6f** | 1.000± 0.000 | -0.060 ± 0.007 | 1.000± 0.000 | -0.119 ± 0.026 |
|  | **Layer M5** | | **Layer M5** | |
|  | **0μM PTX** | **100μM PTX** | **0μM PTX** | **100μM PTX** |
| **Mi1 >> GCaMP6f** | 1.000± 0.000 | -0.195± 0.053 | 1.000± 0.000 | -0.071 ± 0.009 |
| **Tm3 >> GaMP6f** | 1.000± 0.000 | -0.051 ± 0.003 | 1.000± 0.000 | -0.098 ± 0.001 |
|  | **Layer M9/10** | | **Layer M9/10** | |
|  | **0μM PTX** | **100μM PTX** | **0μM PTX** | **100μM PTX** |
| **Mi1 >> GCaMP6f** | 1.000± 0.000 | -0.059 ± 0.023 | 1.000± 0.000 | -0.040 ± 0.006 |
| **Tm3 >> GaMP6f** | 1.000± 0.000 | -0.038 ± 0.006 | 1.000± 0.000 | -0.052 ± 0.004 |
