# Supplementary material for: ON selectivity in the Drosophila visual system is a multisynaptic process involving both glutamatergic and GABAergic inhibition
Source: eLife. 2019 Sep 19;8:e49373. doi: 10.7554/eLife.49373 (PMC6845231; doi:10.7554/eLife.49373)
Supplement: Figure 7—source data 1. — Data related to quantifications shown in main Figure 7, sorted by genotype and experimental condition. [file elife-49373-fig7-data1.docx]

**Figure 7 – source data 1:** Table 1 contains all mean ± s.e.m. data related to quantifications shown in main Figure 7, sorted by genotype and experimental condition.

**Table 1**

| **Figure 7 A** |  |
| --- | --- |
|  | **GluClα mRNA** |
| **FlpSTOP.ND/+** | 1.000 ± 0.017 |
| **elav>Flp; FlpSTOP.ND/+** | 0.567 ± 0.107 |

| **Figure 7 C** |  |
| --- | --- |
|  | **ON Step Layer M1** |
| **Mi1 >> GCaMP6f** |  |
| UAS-Flp, FlpStop.ND/Df | 1.879 ± 0.321 |
| UAS-Flp, FlpStop.ND/+ | 2.122 ± 0.379 |
| FlpStop.ND/Df | 1.843 ± 0.258 |

| **Figure 7 D** |  |
| --- | --- |
|  | **GluClα mRNA** |
| **+/+** | 1.000± 0.000 |
| **elav>GluClα dsRNA** | 0.164 ± 0.740 |

| **Figure 7 E,F** |  |  |
| --- | --- | --- |
|  | **ON Step Layer M1** | |
|  | **Ctrl** | **+ GluClα dsRNA** |
| **Mi1 >> GCaMP6f** | 1.803 ± 0.342 | 1.403 ± 0.308 |
| **Tm3 >> GaMP6f** | 1.908 ± 0.621 | 1.702 ± 0.512 |

| **Figure 7 G** |  |
| --- | --- |
|  | **GluClα mRNA** |
| **FlpSTOP.D/+** | 0.401 ± 0.022 |
| **Df/+** | 0.402 ± 0.082 |
| **FlpSTOP.D/Df** | 0.037 ± 0.060 |

| **Figure 7 H,I** |  |
| --- | --- |
|  | **ON Step Layer M1** |
| **Mi1 >> GCaMP6f** |  |
| FlpStop.D/Df | -0.430 ± 0.036 |
| FlpStop.D/+ | 2.485 ± 0.545 |
| Df/+ | 1.383 ± 0.408 |
| **Tm3 >> GaMP6f** |  |
| FlpStop.D/Df | 0.111 ± 0.086 |
| FlpStop.D/+ | 2.978 ± 0.252 |
| Df/+ | 4.564 ± 0.647 |
