# Supplementary material for: ON selectivity in the Drosophila visual system is a multisynaptic process involving both glutamatergic and GABAergic inhibition
Source: eLife. 2019 Sep 19;8:e49373. doi: 10.7554/eLife.49373 (PMC6845231; doi:10.7554/eLife.49373)
Supplement: Figure 8—source data 1. — Data related to quantifications shown in main Figure 8, sorted by genotype and experimental condition. [file elife-49373-fig8-data1.docx]

**Figure 8 – source data 1:** Table 1 contains all mean ± s.e.m. data related to quantifications shown in main Figure 8, sorted by genotype and experimental condition.

**Table 1**

| **Figure 8 C** |  |  |  |  |
| --- | --- | --- | --- | --- |
|  | **ON Edge** | | | |
| **T4/T5 >> GCaMP6f** | **Layer A** | | **Layer B** | |
|  | **PD** | **DSI** | **PD** | **DSI** |
| FlpStop.D/Df | 0.284 ± 0.058 | 0.156 ± 0.030 | 0.248 ± 0.068 | 0.136 ± 0.034 |
| FlpStop.D/+ | 1.640 ± 0.066 | 0.502 ± 0.019 | 1.940 ± 0.125 | 0.568 ± 0.016 |
| Df/+ | 1.289 ± 0.306 | 0.361 ± 0.047 | 1.451 ± 0.358 | 0.387 ± 0.079 |
|  |  |  |  |  |
|  | **Layer C** | | **Layer D** | |
|  | **PD** | **DSI** | **PD** | **DSI** |
| FlpStop.D/Df | 0.195 ± 0.045 | 0.124 ± 0.022 | 0.266 ± 0.067 | 0.139 ± 0.032 |
| FlpStop.D/+ | 2.044 ± 0.108 | 0.598 ± 0.016 | 1.800 ± 0.107 | 0.573 ± 0.014 |
| Df/+ | 1.469 ± 0.318 | 0.437 ± 0.053 | 1.334 ± 0.170 | 0.414 ± 0.045 |

| **Figure 8 D** |  |  |  |  |
| --- | --- | --- | --- | --- |
|  | **OFF Edge** | | | |
| **T4/T5 >> GCaMP6f** | **Layer A** | | **Layer B** | |
|  | **PD** | **DSI** | **PD** | **DSI** |
| FlpStop.D/Df | 1.548 ± 0.162 | 0.504 ± 0.035 | 1.345 ± 0.165 | 0.468 ± 0.0386 |
| FlpStop.D/+ | 1.986 ± 0.120 | 0.550 ± 0.025 | 1.981 ± 0.063 | 0.624 ± 0.007 |
| Df/+ | 1.858 ± 0.420 | 0.477 ± 0.054 | 1.543 ± 0.303 | 0.433 ± 0.048 |
|  |  |  |  |  |
|  | **Layer C** | | **Layer D** | |
|  | **PD** | **DSI** | **PD** | **DSI** |
| FlpStop.D/Df | 1.237 ± 0.131 | 0.395 ± 0.027 | 1.398 ± 0.155 | 0.425 ± 0.051 |
| FlpStop.D/+ | 1.690 ± 0.073 | 0.570 ± 0.013 | 1.391 ± 0.084 | 0.527 ± 0.023 |
| Df/+ | 1.536 ± 0.258 | 0.426 ± 0.053 | 1.438 ± 0.215 | 0.484 ± 0.062 |

| **Figure 8 G,H** |  |  |  |  |
| --- | --- | --- | --- | --- |
|  | **ON Step** | | **OFF Step** | |
| **T4/T5 >> GCaMP6f** | **Axon terminals** | **Dendrites (T4)** | **Axon terminals** | **Dendrites (T4)** |
| FlpStop.D/Df | -0.252 ± 0.043 | -0.190 ± 0.036 | 0.425 ± 0.055 | 0.145 ± 0.027 |
| FlpStop.D/+ | 0.101 ± 0.013 | 0.262 ± 0.014 | 0.192 ± 0.032 | -0.075 ± 0.009 |
| Df/+ | 0.241 ± 0.084 | 0.351 ± 0.113 | 0.241 ± 0.084 | -0.086 ± 0.020 |
