# Supplementary material for: ON selectivity in the Drosophila visual system is a multisynaptic process involving both glutamatergic and GABAergic inhibition
Source: eLife. 2019 Sep 19;8:e49373. doi: 10.7554/eLife.49373 (PMC6845231; doi:10.7554/eLife.49373)
Supplement: Source code 1. [file elife-49373-code1.pdf]

Source Code File

This document contains all codes (and related sub-functions) for analyzing and plotting responses to 5sec ON/OFF full field flashes (fff) and ON/OFF moving edges stimuli.

Full field flash analysis code

Script name: fff_analysis_elife

Sub-functions needed:

- data_base_select_samples_elife.m
- load_genotypes_conditions_elife.m
- create_experimental_conditions_structure_elife.m
- load_neuron_data10Hz_byLayer_elife.m
- load_neuron_data10Hz_elife.m
- BASELINE_aggregate_fffall_means10Hz_BleedThruFix_elife.m
- mean_cat_elife.m
- fff_stimulus_response_filters_elife.m
- niceaxes.m
- plot_err_patch_v2.m

```
% Analysis and plotting script for ON / OFF full field flashes (fff).
% It loads and analyze all files for given genotypes conditions given in a txt file. It saves variables for future use.
% file (automatized mode)or for an specific genotype (non-automatized
% mode)

% Author: Sebastian Molina-Obando

opengl software
clear all;
close all;

%add the path of needed functions
addpath(genpath('C:\MATLAB_FOLDER\TP_code_Seb'));

%% USER INPUT: GENERAL CONSTANTS
% The user need to define this.
Automatized = true; %"True" if a txt file for the automatized mode is used. Otherwise, write "false".
outlier_filter = false; % "True" for filetering out the outliers on ROI matrix. Otherwise, write "false".
outlier_measure = 'Integral'; % It defines the measurement for outliers (Step, Integral...)
save_data = true; %"True" for saving data (variables and plots) in a folder in your computer. Otherwise, write "false".
```

```
save_in_folder = 'C:\MATLAB_FOLDER\2p\2p_pData\5secfff\eLife\GenotypesData\'; % Write the path of the folder



%% USER INPUT: CONSTANTS FOR THE NON-AUTOMATIZED MODE
% The user need to define this only when using the non-automatized mode.
% If theAutomatized mode is used, the constants will be automatically
% redefine as stated in the txt file.

% General Information
GENOTYPE = 'Mi1_LexA_LexAop_GCaMP6f_GluCla.Flp.D_+'; % This GENOTYPE name will be used to save the data in the computer
PAIRED_CONTROL = 'Mi4_LexA_LexAop_GCaMP6f_GluCla.Flp.D_+'; % It is the existing GENOTYPE data of the paired-control (e.g. responses before DRUG are paired with responses after DRUG)
paired_data = false; %"True" if the current analyzed GENOTYPE has a PAIRED_CONTROL. Otherwise, write "false".
experimental_conditions = 'i_fff5s.*i_Mi1_LexA_LexAop_GCaMP6f_GluClaFlpD.*i_GluCla_KO_exp'; % Concatenation of all conditions that define the experimental GENOTYPE
NEURONAL_TYPE = 'Mi1'; %Write the the cell type being analyzed. Each cell type responses are plotted with individualized axis
NEURON_POLARITY = 'ON'; %Select the cell-type response polarity to apply polarity-spesific response filters. Write 'ON', 'OFF' or '' (blank, in the case of not defined polarity)
BASELINE = 'mean'; %Define what is the baseline (F) signal for the dF/F analysis. Write 'ON' > baseline = mean response at the end of the ON stimulus, 'OFF' > baseline = mean response at the end of the OFF stimulus, or 'mean' > baseline = whole trace mean
NORMALIZE_ROI = false; %"True" to NORMALIZE the response of each ROI (here, a cell). Otherwise, write "false". By defaul it normalized all the values to the max value in the response trace.
NORMALIZE_FLY = true; %"True" to NORMALIZE the response of each FLY (mean of cells). Otherwise, write "false".
NORMALIZATION_TYPE = 'ON_Step';% Choose between 'ON_Step', 'OFF_Step', 'ON_Integral', 'OFF_Integral', or 'Max'
analysis_by_layer = true; %"True" if the selected ROIs (cells) have been categorized in different layers. Otherwise, write "false".
LAYER = 'M1'; %Select the layer to be analyzed. (E.g. 'M1', 'M2', 'M5', 'M9', 'Ax', 'De', 'Lo')
UseControlIDs = false;
col1 = [99/256 99/256 99/256] ;%Define the color for the mean trace. (here, grey)
col2 = [189/256 189/256 189/256];%Define the color for the sem patch trace. (here, light grey)

% Input the values for the different response filters

stimulus_response_correlation_filter = 0; % Correlation value to filter non-correlated responses. (1 = correlated, -1 = anticorrelated, 0 = non-correlated)
ApplyThreshold_ON = false; %"True" to apply a treshold for ON-responding ROIs. Otherwise, write "false".
ApplyThreshold_OFF = false; %"True" to apply a treshold for OFF-responding ROIs. Otherwise, write "false".
Threshold_ON = 2.5; %Value for the ON filter. The final treshold value will be  = Treshold_ON*std of the 1 sec preciding the ON step
Threshold_OFF = 2.5;%Value for the OFF filter. The final treshold value will be  = Treshold_OFF*std of the 1 sec preciding the OFF step
ApplyPeakThreshold = false; %"True" to apply dF/F absolute threshold. Otherwise, write "false".
Threshold_Peak = 0.5; %dF/F threshold, absolut value.
Apply_STD_filter = true; %"True" to apply an std filter of 0.2 to discard noisy/moving ROIs. Otherwise, write "false". The std is checked when the neuron is not responding to the stimulus.

%% Selecting samples from Database
```

```
pdatapath=('C:\MATLAB_FOLDER\2p\2p_pData'); % Path where the pData files are. (pData
files contains all the raw data per ROI)
cd(pdatapath);
database_select_samples_elife; %Here all the different experimental conditions are added (E.g.
genotype, stimulus presented, experiment number, quality of recording, DRUG...)

%% Automatized Analysis: data selection from a txt file list
% A 'Genotype_List.txt' file is needed in the "pdatapath". This contains
% the list of all the genotypes, general information and filter conditions
% to be applied in every case.

stopForLoop = false;

  for aut_i = 1:length(GENOTYPE)
     %Here we load all the information per genotype in every loop
     if stopForLoop
        break
     end
     if Automatized
        close all
        [GENOTYPE, PAIRED_CONTROL,paired_data,analysis_by_layer,LAYER,...
        ApplyThreshold_ON,Threshold_ON,ApplyThreshold_OFF,Threshold_OFF,...
        ApplyPeakThreshold,
Threshold_Peak,BASELINE,NORMALIZE_ROI,NORMALIZE_FLY,...
        NORMALIZATION_TYPE,NEURON_POLARITY,stimulus_response_correlation_filter,
experimental_conditions,Apply_STD_filter]...

=load_genotype_conditions_elife(Automatized,GENOTYPE,PAIRED_CONTROL,paired_data,a
nalysis_by_layer,LAYER,...
        ApplyThreshold_ON,Threshold_ON,ApplyThreshold_OFF,Threshold_OFF,...
        ApplyPeakThreshold,
Threshold_Peak,BASELINE,NORMALIZE_ROI,NORMALIZE_FLY,...

NORMALIZATION_TYPE,NEURON_POLARITY,stimulus_response_correlation_filter,Apply_ST
D_filter);

        GENOTYPE = char(GENOTYPE(aut_i));
        PAIRED_CONTROL = char(PAIRED_CONTROL(aut_i));
        paired_data = paired_data(aut_i);
        analysis_by_layer = analysis_by_layer(aut_i);
        LAYER = char(LAYER(aut_i));
        ApplyThreshold_ON = ApplyThreshold_ON(aut_i);
        Threshold_ON  = Threshold_ON(aut_i);
        ApplyThreshold_OFF = ApplyThreshold_OFF(aut_i);
        Threshold_OFF = Threshold_OFF(aut_i);
        ApplyPeakThreshold = ApplyPeakThreshold(aut_i);
        Threshold_Peak = Threshold_Peak(aut_i);
        Apply_STD_filter = Apply_STD_filter(aut_i);
        BASELINE = char(BASELINE(aut_i));
        NORMALIZE_ROI =  NORMALIZE_ROI(aut_i);
        NORMALIZE_FLY = NORMALIZE_FLY(aut_i);
        NORMALIZATION_TYPE = char(NORMALIZATION_TYPE(aut_i));
        NEURON_POLARITY = char(NEURON_POLARITY(aut_i));
        stimulus_response_correlation_filter = stimulus_response_correlation_filter(aut_i);
        experimental_conditions = find(eval(char(experimental_conditions(aut_i)))); %
experimental_conditions

        if NORMALIZE_ROI
           gen_str = [GENOTYPE ' Normalized responses']
```

```
            else
                gen_str = GENOTYPE;
            end

        else
            experimental_conditions  = find(eval(experimental_conditions));
            stopForLoop = true;

            if NORMALIZE_ROI
                gen_str = [GENOTYPE ' Normalized responses to ROIs']
            else
                gen_str = GENOTYPE;
            end
        end

        %% ANALYSIS
        % Here we create a experimental conditions structure which contains
        % the pData files matching the defined "experimental_conditions"
        ExpCondStrct = create_experimental_conditions_structure_elife(experimental_conditions);


        % Here we interpolates our data at 10 Hz
        if analysis_by_layer == true
            IntExpCondStrct = load_neuron_data10Hz_byLayer_elife(ExpCondStrct,pdatapath,
LAYER);

        else
            IntExpCondStrct = load_neuron_data10Hz_elife(ExpCondStrct,pdatapath);
        end

        % Baseline calculation, dF/F and aggregation (trial average) of all selected ROI responses;
          ROIresponses =
BASELINE_aggregate_fffall_means10Hz_BleedThruFix_elife(IntExpCondStrct,BASELINE);


        %% Data rearrangement and  mean across flies calculation

        iRATE = 10; %rate at which data was interpolated
        dur = size(ROIresponses.rats,2);
        DURS = 5; % duration of the stimulus epoch (e.g. 5 sec for ON or OFF)
        % We start defining matrices to work with. Cur_mat will have all
        % the ROIs responses after aggregation (trial average).
        cur_mat = ROIresponses.rats;
        cur_IDs = ROIresponses.flyID;
        cur_stims = ROIresponses.stims;
        Stimulus_trace = cur_stims;
        inds = ~isnan(sum(cur_mat,2));%Discards NaNs values in the dataset
        cur_mat = cur_mat(inds,:);
        cur_IDs = cur_IDs(inds);
        cur_t = [1:size(cur_mat,2)]/iRATE;% Converts the time vector in seconds units

        %Calculating means across flies. x  > mean across ROIS per fly,
        %m  > mean across fly means, e > sem across fly means
        [x_all,m_all,e_all] = mean_cat_elife(cur_mat,1,cur_IDs);

       %% Epochs definition
       switch NEURON_POLARITY
          case 'ON'
```

```
            epoch_start = 51;
            epoch_end = 100;
        case 'OFF'
            epoch_start = 1;
            epoch_end = 50;
    end

    %% Outlier Analysis
    %This filter gets rid of the the outliers
    if outlier_filter &&  ~paired_data %The filter is apply if...
        % ...chosen by the user (outlier_filter = true)
        % ...and if the experiment condition is a control (defined as "not paired")

        ROI_mat = cur_mat;
            switch outlier_measure
                case 'Integral'
                    outlier_variable = sum(ROI_mat(:,epoch_start:epoch_end),2);
                case 'Step'
                    outlier_variable = max(ROI_mat(:,epoch_start:epoch_start+10),[],2);
            end

        inds_outliers = isoutlier(outlier_variable);
        inds_response_filter = find(~ inds_outliers);
        cur_mat = cur_mat(inds_response_filter,:);
        cur_IDs = cur_IDs(inds_response_filter);
    end
     %% Obtaining Mean, flyMeans, and Error
     % For paired data, it loads the already existing information of its control
     % condition in order to apply the same filters
     if paired_data == true
         if NORMALIZE_ROI

load(['C:\MATLAB_FOLDER\2p\2p_pData\5secfff\NORMALIZED_DATA\',PAIRED_CONTROL,'
\',LAYER], 'indices', 'indices_responding','indices_dF_F_Threshold','ROI_max_control');

         else
             load(['C:\MATLAB_FOLDER\2p\2p_pData\5secfff\',PAIRED_CONTROL,'\',LAYER],
'indices', 'indices_responding','indices_dF_F_Threshold','final_mat','final_IDs');
             final_mat_control = final_mat;
             final_IDs_control = final_IDs;
         end

         final_mat = cur_mat(indices,:);
         final_IDs = cur_IDs(indices);

         % Applying the filters
         if size(indices,2) ~= size(indices_responding,2)
             final_mat = final_mat(indices_responding,:);
             final_IDs = final_IDs(indices_responding);
         end

         if ApplyPeakThreshold == true
             final_mat = final_mat(indices_dF_F_Threshold,:);
             final_IDs = final_IDs(:,indices_dF_F_Threshold);
         end

         if NORMALIZE_ROI
             final_mat_norm = final_mat./ROI_max_control; % Normalized by dividing by the max
responses for each ROI
```

```
            final_mat = final_mat_norm;
        end

        % Getting final means and errors for experimental conditions
        % and its corresponding control
        [x_final,m_final,e_final] = mean_cat_elife(final_mat,1,final_IDs);
        [x_final_control,m_final_control,e_final_control] =
mean_cat_elife(final_mat_control,1,final_IDs_control);

        % Normalization by control response
        if NORMALIZE_FLY && paired_data %The normalization is apply if...
        % ...chosen by the user (NORMALIZE_FLY = true)
        % ...and if the experiment condition is paired with a control (defined as "paired_data")
            % The data can be normalized to disctinc measurements:
            % "Max" > Max response value along the trace
            % "X_Step" > distance between mean of the values preceding
            % the epoch and the max response during that epoch.
            % "X_Integral" > sum of all values during that epoch.
            switch NORMALIZATION_TYPE
                case 'Max'
                    Fly_max = max(x_final_control,[],2)*ones(1,length(x_final_control(1,:)));
                    x_final_norm = x_final./Fly_max;
                    e_final_norm = std(x_final_norm)/sqrt(size(x_final_norm,1));
                case 'ON_Step'
                    FlyOnStep = (abs(max(x_final_control(:,45:60),[],2)) +
abs(mean(x_final_control(:,40:50),2)))*ones(1,length(x_final_control(1,:)));
                    x_final_norm = x_final./FlyOnStep;
                    e_final_norm = std(x_final_norm)/sqrt(size(x_final_norm,1));
                case 'OFF_Step'
                    FlyOffStep = (abs(max(x_final_control(:,95:110),[],2)) +
abs(mean(x_final_control(:,90:100),2)))*ones(1,length(x_final_control(1,:)));
                    x_final_norm = x_final./FlyOffStep;
                    e_final_norm = std(x_final_norm)/sqrt(size(x_final_norm,1));
                case 'ON_Integral'
                     FlyOnIntegral =
sum(x_final_control(:,51:100),2)*ones(1,length(x_final_control(1,:)));
                     x_final_norm = x_final./FlyOnIntegral;
                     e_final_norm = std(x_final_norm)/sqrt(size(x_final_norm,1));
                case 'OFF_Integral'
                     FlyOffIntegral =
sum(x_final_control(:,1:50),2)*ones(1,length(x_final_control(1,:)));
                     x_final_norm = x_final./FlyOffIntegral;
                     e_final_norm = std(x_final_norm)/sqrt(size(x_final_norm,1));
            end

        end

    % If the data is not defined as paired and it not going to be
    % normalized to an existing control, the ROIs responses will pass
    % the chossen filter by the user (See "USER INPUT" above)
    else

[x,m,e,mat,IDs,inds,inds_responding,inds_notresponding,cur_mat_notresp,inds_peakThreshold
,ROI_max]...
            = fff_stimulus_response_filters_elife...

(cur_mat,cur_stims,cur_IDs,NEURON_POLARITY,stimulus_response_correlation_filter,ApplyT
hreshold_ON,...
```

```
Threshold_ON,Threshold_OFF,ApplyThreshold_OFF,ApplyPeakThreshold,Threshold_Peak,NO
RMALIZE_ROI,Apply_STD_filter);

            x_final = x;
            m_final = m;
            e_final = e;
            final_mat = mat;
            final_IDs = IDs;
            indices = inds;
            indices_responding = inds_responding;
            indices_dF_F_Threshold = inds_peakThreshold;
            inds_NR = inds_notresponding;
            cur_mat_NR = cur_mat_notresp;
            ROI_max_control = ROI_max;



            if NORMALIZE_FLY
                switch NORMALIZATION_TYPE
                    case 'Max'
                        Fly_max = max(x_final,[],2)*ones(1,length(x_final(1,:)));
                        x_final_norm = x_final./Fly_max;
                        e_final_norm = std(x_final_norm)/sqrt(size(x_final_norm,1));
                    case 'ON_Step'
                        FlyOnStep = (abs(max(x_final(:,45:60),[],2)) +
abs(mean(x_final(:,40:50),2)))*ones(1,length(x_final(1,:)));
                        x_final_norm = x_final./FlyOnStep;
                        e_final_norm = std(x_final_norm)/sqrt(size(x_final_norm,1));
                    case 'OFF_Step'
                        FlyOffStep = (abs(max(x_final(:,95:120),[],2)) +
abs(mean(x_final(:,90:100),2)))*ones(1,length(x_final(1,:)));
                        x_final_norm = x_final./FlyOffStep;
                        e_final_norm = std(x_final_norm)/sqrt(size(x_final_norm,1));
                    case 'ON_Integral'
                         FlyOnIntegral = sum(x_final(:,51:100),2)*ones(1,length(x_final(1,:)));
                         x_final_norm = x_final./FlyOnIntegral;
                         e_final_norm = std(x_final_norm)/sqrt(size(x_final_norm,1));
                    case 'OFF_Integral'
                         FlyOffIntegral = sum(x_final(:,1:50),2)*ones(1,length(x_final(1,:)));
                         x_final_norm = x_final./FlyOffIntegral;
                         e_final_norm = std(x_final_norm)/sqrt(size(x_final_norm,1));

                end
            end
        end

        %% Quantification per Fly
        % The following line calculates: Peak (Max response), Step, Plateau,
        % Peak above plateau and integral for every fly:

        % First, normalization by fly.
        if NORMALIZE_FLY

            % For OFF Step and Peak
               preStimValue = mean(mean(x_final_norm(:,80:100),2));
               postStimValue = mean(mean(x_final_norm(:,100:110),2));
               if preStimValue > postStimValue % Evaluates if the signal is increasing or decreasing
                       FlyOffStep_norm = (abs(min(x_final_norm(:,100:110),[],2)) +
abs(mean(x_final_norm(:,95:100),2)))*-1;
```

```
            FlyOffPeak_norm = abs(min(x_final_norm(:,100:110),[],2))*-1;
        else
            FlyOffStep_norm = abs(max(x_final_norm(:,100:110),[],2)) +
abs(mean(x_final_norm(:,95:100),2));
            FlyOffPeak_norm = abs(max(x_final_norm(:,100:110),[],2));
        end

      % For ON Step and Peak
        preStimValue = mean(mean(x_final_norm(:,30:50),2));
        postStimValue = mean(mean(x_final_norm(:,50:60),2));
        if preStimValue > postStimValue % Evaluates if the signal is increasing or decreasing
            FlyOnStep_norm = (abs(min(x_final_norm(:,50:60),[],2)) +
abs(mean(x_final_norm(:,45:50),2)))*-1;
            FlyOnPeak_norm = abs(min(x_final_norm(:,50:60),[],2))*-1;
        else
            FlyOnStep_norm = abs(max(x_final_norm(:,50:60),[],2)) +
abs(mean(x_final_norm(:,45:50),2));
            FlyOnPeak_norm = abs(max(x_final_norm(:,50:60),[],2));
        end



      % For ON Plateau
        preStimValue = mean(mean(x_final_norm(:,30:50),2));
        postStimValue = mean(mean(x_final_norm(:,90:95),2));
        if preStimValue > postStimValue
            FlyOnPlateau_norm = (abs(mean(x_final_norm(:,90:95),2)) +
abs(mean(x_final_norm(:,45:50),2)))*-1;
        else
            FlyOnPlateau_norm = abs(mean(x_final_norm(:,90:95),2)) +
abs(mean(x_final_norm(:,45:50),2));
        end

      % For OFF Plateau
        preStimValue = mean(mean(x_final_norm(:,80:100),2));
        postStimValue = mean(mean(x_final_norm(:,40:45),2));
        if preStimValue > postStimValue
            FlyOffPlateau_norm = (abs(mean(x_final_norm(:,40:45),2)) +
abs(mean(x_final_norm(:,90:95),2)))*-1;
        else
            FlyOffPlateau_norm = abs(mean(x_final_norm(:,40:45),2)) +
abs(mean(x_final_norm(:,90:95),2));
        end

      %For Peaks above plateau
         FlyOnPeakAbovePlateau_norm = FlyOnStep_norm - FlyOnPlateau_norm;
         FlyOffPeakAbovePlateau_norm = FlyOffStep_norm - FlyOffPlateau_norm;

      % For ON Integral responses
         FlyOnIntegral_norm = sum(x_final_norm(:,51:100),2);
         FlyOffIntegral_norm = sum(x_final_norm(:,1:50),2);
    end

    % Second, no normalization by fly.
      % For OFF Step and Peak
        preStimValue = mean(mean(x_final(:,80:100),2));
        postStimValue = mean(mean(x_final(:,100:110),2));
        if preStimValue > postStimValue
            FlyOffStep = (abs(min(x_final(:,100:110),[],2)) + abs(mean(x_final(:,95:100),2)))*-
1;
```

```
        FlyOffPeak = abs(min(x_final(:,100:110),[],2))*-1;
    else
        FlyOffStep = abs(max(x_final(:,100:110),[],2)) + abs(mean(x_final(:,95:100),2));
        FlyOffPeak = abs(max(x_final(:,100:110),[],2));
    end

  % For ON Step and Max
    preStimValue = mean(mean(x_final(:,30:50),2));
    postStimValue = mean(mean(x_final(:,50:60),2));
    if preStimValue > postStimValue
        FlyOnStep = (abs(min(x_final(:,50:60),[],2)) + abs(mean(x_final(:,45:50),2)))*-1;
        FlyOnPeak = abs(min(x_final(:,50:60),[],2))*-1;
    else
        FlyOnStep = abs(max(x_final(:,50:60),[],2)) + abs(mean(x_final(:,45:50),2));
        FlyOnPeak = abs(max(x_final(:,50:60),[],2));
    end



  % For ON Plateau
    preStimValue = mean(mean(x_final(:,30:50),2));
    postStimValue = mean(mean(x_final(:,90:95),2));
    if preStimValue > postStimValue
        FlyOnPlateau = (abs(mean(x_final(:,90:95),2)) + abs(mean(x_final(:,45:50),2)))*-
1;
    else
        FlyOnPlateau = abs(mean(x_final(:,90:95),2)) + abs(mean(x_final(:,45:50),2));
    end

  % For OFF Plateau
    preStimValue = mean(mean(x_final(:,80:100),2));
    postStimValue = mean(mean(x_final(:,40:45),2));
    if preStimValue > postStimValue
        FlyOffPlateau = (abs(mean(x_final(:,40:45),2)) + abs(mean(x_final(:,90:95),2)))*-
1;
    else
        FlyOffPlateau = abs(mean(x_final(:,40:45),2)) + abs(mean(x_final(:,90:95),2));
    end

  %For Peaks above plateau
     FlyOnPeakAbovePlateau = FlyOnStep - FlyOnPlateau;
     FlyOffPeakAbovePlateau = FlyOffStep - FlyOffPlateau;

  % For Integrative Response
     FlyOnIntegral = sum(x_final(:,51:100),2);
     FlyOffIntegral = sum(x_final(:,1:50),2);


%% Plotting FLY MEAN and individual FLY traces

% plot all cells, mean across flies
f1 = figure(1); hold on
subplot(2,1,1);
cm=colormap('lines');
h2 = plot_err_patch_v2(cur_t,m_final,e_final,col1,col2);
title({gen_str; 'Mean by fly. Layer: '; LAYER});
axis('tight');

subplot(2,1,2);
```

```
        plot(cur_t,x_final);

        title(sprintf(['Individual fly means, N = %d ( %d )'],size(x_final,1),size(final_mat,1)))
        axis('tight');
        yLims=ylim;
        set(gcf,'Color','w');
        set(gca,'ytick',[0 : 0.5: 4]);
        niceaxes;

        %% Plotting ROIs
        f2= figure(2); hold on;
        plot(cur_t,final_mat);
        title(['individual ROI means', gen_str, ' ' LAYER]);
        axis('tight');
        yLims=ylim;

        line([DURS DURS],yLims,'color',[0 0 0],'linestyle','--');
        line([0 DURS],[0 0],'color',[0 0 0]);
        set(gcf,'Color','w');
        niceaxes;

        %% Plotting Normalized Data per Fly
        if NORMALIZE_FLY && size(x_final_norm,1) >1
            f4= figure(4); hold on;
            subplot(2,1,1);
            h3 = plot_err_patch_v2(cur_t,mean(x_final_norm),e_final_norm,col1,col2);
            title({'Mean by Fly, Normalized to Control'; gen_str; LAYER});
            axis('tight');

            subplot(2,1,2);
            plot(cur_t,x_final_norm);

            title(sprintf(['Individual fly means, NORMALIZED to Control, N = %d ( %d
    )'],size(x_final_norm,1),size(final_mat,1)));
            axis('tight');
            yLims=ylim;

            set(gcf,'Color','w');
            set(gca,'ytick',[0 : 0.5: 4]);
            niceaxes;

        end

        %% Bar Plotting FLY ON and OFF Steps
        f3 = figure(3); hold on
        for i = 1:4

            if i == 1
                ax = subplot(2,2,i);
                data1 = FlyOnStep';
                data2 = FlyOffStep';
                Step_str = cell(1,2);
                Step_str{1} = 'ON Step';
                Step_str{2} = 'OFF Step';
            elseif i == 2
                ax = subplot(2,2,i);
                data1 = FlyOnStep';
                data2 = FlyOnPlateau';
```

```
    Step_str = cell(1,2);
    Step_str{1} = 'ON Step';
    Step_str{2} = 'ON Plateau';
elseif i == 3
    ax = subplot(2,2,i);
    data1 = FlyOffStep';
    data2 = FlyOffPlateau';
    Step_str = cell(1,2);
    Step_str{1} = 'OFF Step';
    Step_str{2} = 'OFF Plateau';
 elseif i == 4
    ax = subplot(2,2,i);
    data1 = FlyOnIntegral';
    data2 = FlyOffIntegral';
    Step_str = cell(1,2);
    Step_str{1} = 'ON Integral';
    Step_str{2} = 'OFF Integral';
end

mean_data1 = mean(data1);
mean_data2 = mean(data2);
std_data1= std(data1);
std_data2 = std(data2);
sem_data1 = std(data1)/sqrt(size(x_final,1));
sem_data2 = std(data2)/sqrt(size(x_final,1));
yData = [mean_data1 mean_data2];
stdData = [std_data1 std_data2];
semData = [sem_data1 sem_data2];
xData = [1, 2];

col3 = [49/256 130/256 189/256];  % dark blue
col4 = [158/256 202/256 225/256]; % light blue


b = bar(ax,xData,yData,'FaceColor',col1);
hold on;

xErrorData= [1, 2];
for i=1:length(xErrorData)  % iterate over number of bar objects
  e2 = errorbar(xErrorData(i),yData(i),semData(i),'k.');
  e2.LineWidth = 0.75;
end

sz = 5; % Scatter Plot
xData= [1, 2];

for i = 1:size(data1,2)-1
xData = [xData; 1,2];
end

s4 = scatter(xData(:,1), data1,sz, 'filled');
s4.MarkerEdgeColor = 'k';
s4.MarkerFaceColor = col1;
hold on
s5 = scatter(xData(:,2), data2,sz, 'filled');
s5.MarkerEdgeColor = 'k';
s5.MarkerFaceColor = col1;

set(ax,'xticklabel',Step_str)
```

```
    title({'Response'});
    axis('tight');
end
subtitle (gen_str);

%% Bar Plotting FLY ON and OFF Steps NORMALIZED By Fly
if NORMALIZE_FLY && size(x_final_norm,1) >1
    f7 = figure(7); hold on
    for i = 1:4
        if i == 1
            ax = subplot(2,2,i);
            data1 = FlyOnStep_norm';
            data2 = FlyOffStep_norm';
            Step_str = cell(1,2);
            Step_str{1} = 'ON Step';
            Step_str{2} = 'OFF Step';
        elseif i == 2
            ax = subplot(2,2,i);
            data1 = FlyOnStep_norm';
            data2 = FlyOnPlateau_norm';
            Step_str = cell(1,2);
            Step_str{1} = 'ON Step';
            Step_str{2} = 'ON Plateau';
        elseif i == 3
            ax = subplot(2,2,i);
            data1 = FlyOffStep_norm';
            data2 = FlyOffPlateau_norm';
            Step_str = cell(1,2);
            Step_str{1} = 'OFF Step';
            Step_str{2} = 'OFF Plateau';
         elseif i == 4
            ax = subplot(2,2,i);
            data1 = FlyOnIntegral_norm';
            data2 = FlyOffIntegral_norm';
            Step_str = cell(1,2);
            Step_str{1} = 'ON Integral';
            Step_str{2} = 'OFF Integral';
        end

        mean_data1 = mean(data1);
        mean_data2 = mean(data2);
        std_data1= std(data1);
        std_data2 = std(data2);
        sem_data1 = std(data1)/sqrt(size(x_final_norm,1));
        sem_data2 = std(data2)/sqrt(size(x_final_norm,1));
        yData = [mean_data1 mean_data2];
        stdData = [std_data1 std_data2];
        semData = [sem_data1 sem_data2];
        xData = [1, 2];

        col3 = [49/256 130/256 189/256];  % dark blue
        col4 = [158/256 202/256 225/256]; % light blue



        b = bar(ax,xData,yData,'FaceColor',col1);
        hold on;

        xErrorData= [1, 2];
        for i=1:length(xErrorData)  % iterate over number of bar objects
```

```
            e2 = errorbar(xErrorData(i),yData(i),semData(i),'k.');
            e2.LineWidth = 0.75;
          end

          sz = 5; % Scatter Plot
          xData= [1, 2];

          for i = 1:size(data1,2)-1
          xData = [xData; 1,2];
          end

          s4 = scatter(xData(:,1), data1,sz, 'filled');
          s4.MarkerEdgeColor = 'k';
          s4.MarkerFaceColor = col1;
          hold on
          s5 = scatter(xData(:,2), data2,sz, 'filled');
          s5.MarkerEdgeColor = 'k';
          s5.MarkerFaceColor = col1;

          set(ax,'xticklabel',Step_str)
          title({'Normalyzed by Control Fly'});
          axis('tight');
    end
end
subtitle (gen_str);

%% Plotting FLY MEANs BEFORE AND AFTER applying filters

% plot all cells, mean across flies
f5=figure(5); hold on
subplot(2,1,1);
cm=colormap('lines');
h2 = plot_err_patch_v2(cur_t,m_final,e_final,col1,col2); hold on;
plot(cur_t,x_final);
title({gen_str ;'Mean by fly AFTER Threshold. Layer: '; LAYER})
plot(cur_t, round(mean(ROIresponses.stims))*0.5 + 1)
axis('tight');
yLims=ylim;
set(gcf,'Color','w');
set(gca,'ytick',[0 : 0.5: 4]);
niceaxes;

subplot(2,1,2);
cm=colormap('lines');
h2 = plot_err_patch_v2(cur_t,m_all,e_all,col1,col2); hold on;
plot(cur_t,x_all);
title({gen_str ; 'Mean by fly BEFORE Threshold. Layer: '; LAYER})
plot(cur_t, round(mean(ROIresponses.stims))*0.5 + 1)
axis('tight');
yLims=ylim;
set(gcf,'Color','w');
set(gca,'ytick',[0 : 0.5: 4]);
niceaxes;

%% Plotting All ROIs BEFORE AND AFTER applying filters

% plot all cells, mean across flies
f6=figure(6); hold on
```

```matlab
        subplot(2,1,1);
        plot(cur_t,cur_mat);
        title({'# of ROI BEFORE Threshold'; gen_str; LAYER; size(cur_mat,1)});
        axis('tight')
        yLims=ylim;

        line([DURS DURS],yLims,'color',[0 0 0],'linestyle','--');
        line([0 DURS],[0 0],'color',[0 0 0]);
        set(gcf,'Color','w');
        niceaxes;

        subplot(2,1,2);
        plot(cur_t,final_mat);
        title({'# of ROI AFTER Threshold'; gen_str; LAYER;size(final_mat,1)});
        axis('tight')
        yLims=ylim;

        line([DURS DURS],yLims,'color',[0 0 0],'linestyle','--');
        line([0 DURS],[0 0],'color',[0 0 0]);
        set(gcf,'Color','w');
        niceaxes;

        %% Saving Data

        if save_data
            saveInFolder = [save_in_folder,GENOTYPE,'\',LAYER];
            if NORMALIZE_ROI
                cd('C:\MATLAB_FOLDER\2p\2p_pData\5secfff\NORMALIZED_DATA')
                allFolders = dir;
                listing = dir(GENOTYPE);
                if isempty(listing)
                    mkdir(GENOTYPE)
                end
                saveInFolder =
['C:\MATLAB_FOLDER\2p\2p_pData\5secfff\NORMALIZED_DATA\',GENOTYPE,'\',LAYER];
                save(saveInFolder, 'FlyOnStep','FlyOffStep','FlyOnPlateau','FlyOffPlateau',
'FlyOnPeak','FlyOffPeak'...
                    ,'final_mat','final_IDs','indices','indices_responding','Stimulus_trace'...
                    ,'indices_dF_F_Threshold','ROI_max_control');
                cd(['C:\MATLAB_FOLDER\2p\2p_pData\5secfff\NORMALIZED_DATA\',GENOTYPE])
                saveas(f1,[LAYER,'_5sfff_Normalized.pdf'])
                saveas(f2,[LAYER,'_ROIs_Normalized.pdf'])
                saveas(f3,[LAYER,'_Steps_Normalized.pdf'])
            else

                cd(save_in_folder);
                allFolders = dir;
                listing = dir(GENOTYPE);
                if isempty(listing)
                    mkdir(GENOTYPE)
                end



                if NORMALIZE_FLY
                    if strcmp(NORMALIZATION_TYPE(end-4:end),'egral')

save(saveInFolder,'FlyOffIntegral','FlyOffIntegral_norm','FlyOnIntegral_norm','FlyOnIntegral','-
append');
```

```
                    cd([save_in_folder,GENOTYPE])
                    try
                        saveas(f7,[LAYER,'_Integral_NomarlizedByFlies.pdf'])
                    catch
                        disp('Normalized Data was not saved');
                    end
                else

save(saveInFolder,'FlyOnIntegral','FlyOffIntegral','FlyOnPeak','FlyOnPeak_norm','FlyOffPeak','FlyOffPeak_norm','FlyOnStep','FlyOnStep_norm','FlyOffStep','FlyOffStep_norm','FlyOnPlateau'...

,'FlyOnPlateau_norm','FlyOffPlateau','FlyOffPlateau_norm','FlyOnPeak','FlyOffPeak','FlyOnPeakAbovePlateau','FlyOffPeakAbovePlateau'...

,'FlyOnPeakAbovePlateau_norm','FlyOffPeakAbovePlateau_norm','final_mat','final_IDs','indices'...

,'indices_responding','Stimulus_trace','indices_dF_F_Threshold','x_final','e_final','m_final'...
                        ,'x_final_norm', 'e_final_norm');
                    cd([save_in_folder,GENOTYPE])
                    saveas(f1,[LAYER,'_5sfff.pdf'])
                    saveas(f2,[LAYER,'_ROIs.pdf'])
                    saveas(f3,[LAYER,'_Steps.pdf'])
                    try
                        saveas(f4,[LAYER,'_5sfff_NormalizedByFlies.pdf'])
                        saveas(f7,[LAYER,'_Steps_NomarlizedByFlies.pdf'])
                    catch
                        disp('Normalized Data was not saved');
                    end
                end
            else

save(saveInFolder,'FlyOffIntegral','FlyOnIntegral','FlyOnStep','FlyOffStep','FlyOnPeak','FlyOffPeak','FlyOnPlateau','FlyOffPlateau', 'FlyOnPeak','FlyOffPeak'...

,'FlyOnPeakAbovePlateau','FlyOffPeakAbovePlateau','final_mat','final_IDs','indices','indices_responding','Stimulus_trace'...
                    ,'indices_dF_F_Threshold','x_final','e_final','m_final');
                    cd([save_in_folder,GENOTYPE])
                    saveas(f1,[LAYER,'_5sfff.pdf'])
                    saveas(f2,[LAYER,'_ROIs.pdf'])
                    saveas(f3,[LAYER,'_Steps.pdf'])
            end

        end

    end

    fprintf([gen_str,': N = %d ( %d )'],size(x_final,1),size(final_mat,1));
    end
```

Fff analysis-related script

Script name: data_base_select_samples_elife.m

```
%Script to read in database (txt file) and make various categories for
```

```matlab
%specific experimental conditions. It needs as an input the
%MASTER_oldersummary txt file where all data summary is stored.

%% Reading the data base
pdatapath=('C:\MATLAB_FOLDER\2p\2p_pData'); % Path where the pData files are. (pData
files contains all the raw data per ROI)
cd(pdatapath);
data_base_name = 'MASTER_foldersummary_Seb.txt'; % The file needs to be saved in the
"pdatapath"

[fname,datetime,frames,zdepth,tottime,stimbouts,locnum,...
    activecells,inverted,stimcode,quality,driver,...
    moving,layer,wavelength,flyID, responsivefly, condition1, condition2, condition3]...
    =textread(data_base_name,...
    '%s %s %f %f %f %f %f %s %f %s %f %s %f %s %f %f %f %s %s %s \r',...
    'headerlines',1,'delimiter','\t');


%% Stimulus Codes
% Creating variable for different stimuli. The stimulus names are in the
% "stimcode" column in the data base txt file

% ON - OFF full field flashes:
i_fff5s =
strcmpi('LocalCircle_5sec_220deg_0degAz_0degEl_Sequential_LumDec_LumInc',(stimcode));

% Moving Edges:
i_drifting_edge_20degps_Rand8DIR_IncDec_opt =
strcmpi('DriftingEdge_LumDecLumInc_1period_20degPerSec_90deg_BlankRand_8Dirs_optimi
zed', (stimcode));

%% Genotypes
% Creating variable for different genotypes. The genotypes names are in the
% "driver" column in the data base txt file

% Medulla neurons expressing iGluSnFR
i_Mi1_809_SplitGal4_UAS_iGluSnFR_A =
strcmpi('Mi1_809_SplitGal4_UAS_iGluSnFR_A',driver);
i_Tm3_300_SplitGal4_UAS_iGluSnFR_A =
strcmpi('Tm3_300_SplitGal4_UAS_iGluSnFR_A',driver);

% -------------------------Lamina neurons-----------------------------
i_L1_c202_Gal4_UAS_GCaMP6f = strcmpi('L1_c202_Gal4_UAS_GCaMP6f',driver);

% -------------------------Medulla neurons-----------------------------
% Mi1
i_Mi1_809_SplitGal4_UAS_GCaMP6f = strcmpi('Mi1_809_SplitGal4_UAS_GCaMP6f',driver);

% GluCla dsRNA knock down
i_Mi1_809_UAS_RNAi_GluCla_UAS_GCaMP6f =
strcmpi('Mi1_809_UAS_RNAi_GluCla_UAS_GCaMP6f',driver);
i_Mi1_809_UAS_GCaMP6f_UAS_tdTom =
strcmpi('Mi1_809_UAS_GCaMP6f_UAS_tdTom',driver);

% GluCla cell type-specific loss of function
i_Mi1_UAS_Flp_UAS_GCaMp6f_GluClalphaFlpSTOP_DfED6025 =
strcmpi('Mi1_809_SplitGal4_UAS_Flp_UAS_GCaMp6f_GluClalphaFlpSTOP_DfED6025_',driver
);
```

```
i_Mi1_HeteroyzgousControl_UAS_GCaMp6f_GluClalphaFlpSTOP =
strcmpi('Mi1_809_SplitGal4_HeteroyzgousControl_UAS_GCaMp6f_GluClalphaFlpSTOP_',drive
r);
i_Mi1_NoFlp_UAS_GCaMp6f_GluClalphaFlpSTOP_DfED6025 =
strcmpi('Mi1_809_SplitGal4_NoFlpControl_UAS_GCaMp6f_GluClalphaFlpSTOP_DfED6025_',d
river);

% GluCla full loss of function
i_Mi1_LexA_LexAop_GCaMP6f_DfED6025_GluClaFlpD =
strcmpi('Mi1_LexA_LexAop_GCaMP6f_DfED6025_GluCla.Flp.D',driver);
i_Mi1_LexA_LexAop_GCaMP6f_GluClaFlpD =
strcmpi('Mi1_LexA_LexAop_GCaMP6f_GluCla.Flp.D_+',driver);
i_Mi1_LexA_LexAop_GCaMP6f_DfED6025 =
strcmpi('Mi1_LexA_LexAop_GCaMP6f_DfED6025_+',driver);

% GluCla S278T rescue experiment
i_Mi1_LexA_LexAop_GCaMP6f_GluClaS278T =
strcmpi('Mi1_LexA_LexAop_GCaMP6f_GluClaS278T_+',driver);
i_Mi1_LexA_LexAop_GCaMP6f_GluClaS278T_DfED6025 =
strcmpi('Mi1_LexA_LexAop_GCaMP6f_GluClaS278T_DfED6025_',driver);

% Rdl MDRR rescue experiment
i_Mi1_Lexa_UAS_Gcamp6f_Rdl1_RdlMDRR =
strcmpi('Mi1_Lexa_UAS_Gcamp6f_RDL1+RDLMDR',driver);
i_Mi1_Lexa_UAS_Gcamp6f_Rdl1 =
strcmpi('Mi1_lexA_LexAopGcamp_RDL1background',driver);

%Tm3
i_Tm3_300_SplitGal4_UAS_GCaMP6f = strcmpi('Tm3_300_SplitGal4_UAS_GCaMP6f',driver);

% GluCla dsRNA knock down
i_Tm3_300_UAS_RNAi_GluCla_UAS_GCaMP6f =
strcmpi('Tm3_300_UAS_RNAi_GluCla_UAS_GCaMP6f',driver);
i_Tm3_300_UAS_GCaMP6f_UAS_tdTom =
strcmpi('Tm3_300_UAS_GCaMP6f_UAS_tdTom',driver);

% GluCla full loss of function
i_Tm3_LexA_LexAop_GCaMP6f_DfED6025_GluClaFlpD =
strcmpi('Tm3_LexA_LexAop_GCaMP6f_Df_GluCla.Flp.D',driver);
i_Tm3_LexA_LexAop_GCaMP6f_GluClaFlpD =
strcmpi('Tm3_LexA_LexAop_GCaMP6f_GluCla.Flp.D_+',driver);
i_Tm3_LexA_LexAop_GCaMP6f_DfED6025 =
strcmpi('Tm3_LexA_LexAop_GCaMP6f_DfED6025_+',driver);

% GluCla S278T rescue experiment
i_Tm3_LexA_LexAop_GCaMP6f_GluClaS278T =
strcmpi('Tm3_LexA_LexAop_GCaMP6f_GluClaS278T_+',driver);
i_Tm3_LexA_LexAop_GCaMP6f_GluClaS278T_DfED6025 =
strcmpi('Tm3_LexA_LexAop_GCaMP6f_GluClaS278T_DfED6025_',driver);

% Rdl MDRR rescue experiment
i_Tm3_LexA_lexAop_GCaMP6f = strcmpi('Tm3_LexA_lexAop_GCaMP6f',driver);
i_Tm3_LexA_lexAop_GCaMP6f_RdlMDRR =
strcmpi('Tm3_LexA_lexAop_GCaMP6f_RdlMDRR',driver);

% -------------------------Lobula complex neurons------------------------------
%T4/T5
i_T4_T5_LexA_LexAop_GCaMP6f_28rec =
strcmpi('T4_T5_LexA_LexAop_GCaMP6f_28rec',driver);
```

```
% GluCla full loss of function
i_T4_T5_LexA_LexAop_GCaMP6f_28rec_GluClalphaFlpSTOP_DfED6025=
strcmpi('T4_T5_LexA_LexAop_GCaMP6f_28rec_Df_GluCla.Flp.D',driver);
i_T4_T5_LexA_LexAop_GCaMP6f_28rec_GluClalphaFlpSTOP =
strcmpi('T4_T5_LexA_LexAop_GCaMP6f_28rec_GluCla.Flp.D_+',driver);
i_T4_T5_LexA_LexAop_GCaMP6f_28rec_DfED6025 =
strcmpi('T4_T5_LexA_LexAop_GCaMP6f_28rec_DfED6025_+',driver);

% GluCla S278T rescue experiment
i_T4_T5_LexA_LexAop_GCaMP6f_28rec_GluClaS278T =
strcmpi('T4_T5_LexA_LexAop_GCaMP6f_28rec_GluClaS278T_+',driver);
i_T4_T5_LexA_LexAop_GCaMP6f_28rec_GluClaS278T_DfED6025 =
strcmpi('T4_T5_LexA_LexAop_GCaMP6f_rec28_GluClaS278T_DfED6025_',driver);


%% Esperimentals Conditions
% Creating variable for different conditions. The conditions names are in the
% "condition" columns in the data base txt file

% Condition 1
i_Control = strcmpi('Control', condition1);
i_PTX_Control = strcmpi('PTX_Control', condition1);
i_PTX_0uM = strcmpi('PTX_0uM', condition1);
i_PTX_0_5uM = strcmpi('PTX_0.5uM', condition1);
i_PTX_1uM = strcmpi('PTX_1uM', condition1);
i_PTX_2_5uM = strcmpi('PTX_2.5uM', condition1);
i_PTX_5uM = strcmpi('PTX_5uM', condition1);
i_PTX_25uM = strcmpi('PTX_25uM', condition1);
i_PTX_50uM = strcmpi('PTX_50uM', condition1);
i_PTX_100uM = strcmpi('PTX_100uM', condition1);
i_MPEP_Control = strcmpi('MPEP_Control', condition1);
i_MPEP_100uM = strcmpi('MPEP_100uM', condition1);

% Condition 2
i_Exp1 = strcmpi('Exp1_', condition2);
i_Exp2 = strcmpi('Exp2_', condition2);
i_Common1 = strcmpi('Common1_', condition2);

% Condition 3
i_PTX_exp_0uM = strcmpi('PTX_exp_0uM', condition3);
i_PTX_exp_0_5uM = strcmpi('PTX_exp_0.5uM', condition3);
i_PTX_exp_1uM = strcmpi('PTX_exp_1uM', condition3);
i_PTX_exp_2_5uM = strcmpi('PTX_exp_2.5uM', condition3);
i_PTX_exp_5uM = strcmpi('PTX_exp_5uM', condition3);
i_PTX_exp_25uM = strcmpi('PTX_exp_25uM', condition3);
i_PTX_exp_50uM = strcmpi('PTX_exp_50uM', condition3);
i_PTX_exp_100uM = strcmpi('PTX_exp_100uM', condition3);
i_PTX_exp_0_50uM = strcmpi('PTX_exp_0_50uM', condition3);
i_PTX_exp_5_100uM = strcmpi('PTX_exp_5_100uM', condition3);

i_MPEP_exp_100uM = strcmpi('MPEP_exp_100uM', condition3);
i_GluCla_RNAi_exp = strcmpi('GluCla_RNAi_exp', condition3);

i_GluCla_FlpSTOP_exp = strcmpi('GluCla_FlpSTOP_exp', condition3);
i_GluCla_KD_exp = strcmpi('GluCla_KD_exp', condition3);
i_GluCla_KO_exp = strcmpi('GluCla_KO_exp', condition3);
------------------------------------------------------------------------------------------------------------------------
```

Fff analysis-related script

Script name: load_genotypes_conditions_elife.m

```
% The function loads the experimental conditions listed in a txt file.
% It creates a list of booleans for each condition variable. It needs as an input a txt file
(Genotype_List.txt) with the different the genotype conditions

function [GENOTYPE, PAIRED_CONTROL,UseControlIDs,byLayer,LAYER,...
        ApplyThreshold_ON,Threshold_ON,ApplyThreshold_OFF,Threshold_OFF,...
        ApplyPeakThreshold, Threshold_Peak,BASELINE,NORMALIZE_ROI,NORMALIZE_FLY,...

NORMALIZATION_TYPE,NEURON_POLARITY,CorrelationValue,experimental_conditions,App
ly_STD_filter]...

=load_genotype_conditions_elife(Automatized,GENOTYPE,PAIRED_CONTROL,UseControlID
s,byLayer,LAYER,...
        ApplyThreshold_ON,Threshold_ON,ApplyThreshold_OFF,Threshold_OFF,...
        ApplyPeakThreshold, Threshold_Peak,BASELINE,NORMALIZE_ROI,NORMALIZE_FLY,...
        NORMALIZATION_TYPE,NEURON_POLARITY,CorrelationValue,Apply_STD_filter)

if Automatized
    disp('------------ Automatized Analysis MODE ------------')
    pdatapath=('C:\MATLAB_FOLDER\2p\2p_pData');
    cd(pdatapath);
    [GENOTYPE, PAIRED_CONTROL,UseControlIDs,byLayer,LAYER,...
        ApplyThreshold_ON,Threshold_ON,ApplyThreshold_OFF,Threshold_OFF,...
        ApplyPeakThreshold, Threshold_Peak,BASELINE,NORMALIZE_ROI,NORMALIZE_FLY,...

NORMALIZATION_TYPE,NEURON_POLARITY,CorrelationValue,experimental_conditions,App
ly_STD_filter]=textread('Genotype_List.txt','%s %s %s %s %s %s %f %s %f %s %f %s %s %s
%s %s %f %s %s \r','headerlines',1,'delimiter','\t');

    % Transforming all logical strings to logicals
    UseControlIDs = strcmpi(UseControlIDs, 'true');
    byLayer = strcmpi(byLayer, 'true');
    ApplyThreshold_ON = strcmpi(ApplyThreshold_ON, 'true');
    ApplyThreshold_OFF = strcmpi(ApplyThreshold_OFF, 'true');
    ApplyPeakThreshold = strcmpi(ApplyPeakThreshold, 'true');
    Apply_STD_filter = strcmpi(Apply_STD_filter, 'true');
    NORMALIZE_ROI = strcmpi(NORMALIZE_ROI, 'true');
    NORMALIZE_FLY = strcmpi(NORMALIZE_FLY, 'true');



else
    disp('------------ Not Automatized Analysis MODE ------------');
    experimental_conditions = [];
end
```

Fff analysis-related script

Script name: create_experimental_conditions_structure_elife.m

```
% This function loads the pData files for a particular experimental
% condition
```

```
function out=create_experimental_conditions_structure_elife(inds)
% Takes indices you want to analyze as input inds

% Reads the masterfolder and seperates the different categories

[fname,datetime,frames,zdepth,tottime,stimbouts,locnum,...
    activecells,inverted,stimcode,quality,driver,...
    moving,layer,wavelength,flyID, responsivefly, condition1, condition2, condition3]...
    =textread('MASTER_foldersummary_Seb.txt',...
    '%s %s %f %f %f %f %f %s %f %s %f %s %f %s %f %f %f %s %s %s \r',...
    'headerlines',1,'delimiter','\t');

out=[];

count=1;
for ii=1:length(inds) %Will iterate for all the indices
    if exist(fname{inds(ii)}) %If the index has a file name fname
        x=load(fname{inds(ii)}); %Load the fname of that index
        try
            ns=[1:size(x.strct.dRatio,1)]; %put dRatio in ns variable for all ROIs
        catch
            ns=[1:size(x.strct.dRatio1,1)]; %put dRatio1 in ns variable for all ROIs
        end
        for jj=1:length(ns) %For each ROI in that pData file
            out(count).name=fname{inds(ii)}; % Put the fname in the output structure
            out(count).cell=ns(jj); %Create a cell for that output index and put each ROIs dRatio
            count=count+1;
        end
    end
end
```

Fff analysis-related script

Script name: load_neuron_data10Hz_byLayer_elife.m

```
% This function figures out,among all recorded ROIs, which ROI belongs
% to a specific layer. The identification number of each ROIs in each layer
% is specified in the "layer" column of the masterfolder txt file. For all
% selected ROIs, important information is extracted and created. E.g. the stimulus and
response time vectors are genertaed and
% extrapolated to 10 Hz.

function out=load_neuron_data10Hz_byLayer_elife(in,locdir, layerTypeWanted)

% Reads the masterfolder and seperates the different categories

[fname,datetime,frames,zdepth,tottime,stimbouts,locnum,...
    activecells,inverted,stimcode,quality,driver,...
    moving,layer,wavelength,flyID, responsivefly, condition1, condition2, condition3]...
    =textread('MASTER_foldersummary_Seb.txt',...
    '%s %s %f %f %f %f %f %s %f %s %f %s %f %s %f %f %f %s %s %s \r',...
    'headerlines',1,'delimiter','\t');


currd=pwd;
cd(locdir);
```

```
count=1;
for ii=1:length(in)  % all the presentations of the correct stimulus by ROI
    qq=in(ii).cell;  % which ROI 'cell' we are currently on.
    ss=strcmpi(in(ii).name,fname);  % logical that tells you which LDM out of all of them we are
currently on.
    if exist(in(ii).name) * (qq>0)
        x=load(in(ii).name);  % open that data file
        currLayerInfo=layer{ss};
        % now use this info to determine if the current "cell" value stored
        % in qq matches any of the numbers that follow the "layer" that
        % we want  e.g. LPA 1:5
        str_i_layer=strfind(currLayerInfo, layerTypeWanted); % gives you the first location of
"layerTypeWanted" in "currLayerInfo"

        if(~isempty(str_i_layer))
            str_start= str_i_layer + length(layerTypeWanted)-1;
            i=str_start; % index one before where the numbers will start

            while(true)
                i=i+1;

                if(isletter(currLayerInfo(i))) % a letter denotes next layer "label tag"
                    break;
                end

                if(i==length(currLayerInfo)) % end of the layer string
                    i=i+1; % this is the last number so step one more to get the indexing to include it....
                    break;
                end

            end
            %Identification numbers of the ROI's that we want the data from
            layersToUse=str2num(currLayerInfo(str_start+1:i-1));

            if(isfield(x.strct,'dSignal2')) % For two channel imaging
               x.strct.dRatio1 = x.strct.dSignal2;
            else
               (isfield(x.strct,'dSignal1'));% For one channel imaging
                x.strct.dRatio1 = x.strct.dSignal1;
            end

            if qq<=size(x.strct.dRatio1,1) && sum(layersToUse==qq) % Variables for the current
ROI are stored if...
                % ...this ROI is within the "layerTypeWanted" list of number of ROIs recorded
                % ...and if this ROI was listed within the wanted layer.
                out(count).stimcode=stimcode{ss};
                out(count).quality=quality(ss);
                out(count).driver=driver{ss};
                out(count).layer=layer(ss);
                out(count).wavelength=wavelength(ss);
                out(count).flyID=flyID(ss);
                out(count).name=in(ii).name;
                out(count).cell=in(ii).cell;
                try
                    out(count).zlayer = x.strct.layer;
                catch
                    disp(fprintf('This pData: %s  does not have (z)layer information', out(count).name));
                end
                out(count).xml=x.strct.xml;
```

```
            out(count).ratio=x.strct.dRatio1(qq,:);
            out(count).stim = x.strct.fstimval;
            out(count).raw_stim = x.strct.ch3;
            out(count).avrstimval = x.strct.avrstimval;
            out(count).frame_nums = x.strct.frame_nums;
            fps=x.strct.xml.framerate;
            out(count).t=[1:length(out(count).ratio)]/fps; %Here we generate the time vector: 
frames / framerate
            out(count).it=[0.5/fps:0.1:(length(out(count).ratio)+0.5)/fps]; %New time vector for 
interpolation at 10Hz (0.1), starts and ends with 0.5/fps shift

            %Nearest neighbor interpolation for the stimulus (should maintain discrete values) of 
.stim (at 10Hz)
            out(count).istim=interp1(out(count).t,out(count).stim,out(count).it,'nearest','extrap');


            %Linear interpolation for the response/signal (ratio)
            out(count).iratio=interp1(out(count).t,out(count).ratio,out(count).it,'linear','extrap');


            % Added stimpos and centers  interpl is based on the length of out.t
            if(isfield(x.strct,'fstimpos1'))

out(count).ifstimpos1=interp1(out(count).t,x.strct.fstimpos1,out(count).it,'nearest','extrap');

out(count).ifstimpos2=interp1(out(count).t,x.strct.fstimpos2,out(count).it,'nearest','extrap');

            end

            if(isfield(x.strct,'cell_nums'))
                out(count).ref_cell_num = x.strct.cell_nums(qq);
            end
            count=count+1;
        end
    else
        disp('No layer information for this fly:'); % Message to be displayed if the layer 
information was not found for a specific fly
        in(ii).name
        layersToUse= [1:length(x.strct.masks)]; % If no layer, it takes all selected ROIs and does 
the same analyis (see above)

        if(isfield(x.strct,'dSignal2'))
          x.strct.dRatio1 = x.strct.dSignal2;
        else
          (isfield(x.strct,'dSignal1'));
            x.strct.dRatio1 = x.strct.dSignal1;
        end

        if qq<=size(x.strct.dRatio1,1) && sum(layersToUse==qq)
            out(count).stimcode=stimcode{ss};
            out(count).quality=quality(ss);
            out(count).driver=driver{ss};
            out(count).layer=layer(ss);
            out(count).wavelength=wavelength(ss);
            out(count).flyID=flyID(ss);
            out(count).name=in(ii).name;
            out(count).cell=in(ii).cell;
            try
                out(count).zlayer = x.strct.layer;
```

```
            catch
               disp(fprintf('This pData: %s  does not have (z)layer information', out(count).name));
            end
            out(count).xml=x.strct.xml;
            out(count).ratio=x.strct.dRatio1(qq,:);
            out(count).stim = x.strct.fstimval;
            out(count).raw_stim = x.strct.ch3;
            out(count).avrstimval = x.strct.avrstimval;
            out(count).frame_nums = x.strct.frame_nums;
            fps=x.strct.xml.framerate;
            out(count).t=[1:length(out(count).ratio)]/fps;
            out(count).it=[0.5/fps:0.1:(length(out(count).ratio)+0.5)/fps];
            out(count).istim=interp1(out(count).t,out(count).stim,out(count).it,'nearest','extrap');
            out(count).iratio=interp1(out(count).t,out(count).ratio,out(count).it,'linear','extrap');

            if(isfield(x.strct,'fstimpos1'))

out(count).ifstimpos1=interp1(out(count).t,x.strct.fstimpos1,out(count).it,'nearest','extrap');

out(count).ifstimpos2=interp1(out(count).t,x.strct.fstimpos2,out(count).it,'nearest','extrap');

            end

            if(isfield(x.strct,'centers'))
               out(count).center=x.strct.centers(qq,:);
            end

            if(isfield(x.strct,'barResponse'))
               out(count).barResponse=x.strct.barResponse(qq);
            end


            if(isfield(x.strct,'cell_nums'))
               out(count).ref_cell_num = x.strct.cell_nums(qq);
            end
            count=count+1;
         end
      end

   else
      disp([in(ii).name ' unfound -- skipping']);
   end
end

cd(currd);
```

Fff analysis-related script

Script name: load_neuron_data10Hz_elife.m

```
% For all selected ROIs, important information is extracted and created. E.g. the stimulus and response time vectors are genertaed and
% extrapolated to 10 Hz.

function out=load_neuron_data10Hz_elife(in,locdir)

[fname,datetime,frames,zdepth,tottime,stimbouts,locnum,...
```

```
    activecells,inverted,stimcode,quality,driver,...
    moving,layer,wavelength,flyID, responsivefly, condition1, condition2, condition3]...
    =textread('MASTER_foldersummary_Seb.txt',...
    '%s %s %f %f %f %f %f %s %f %s %f %s %f %s %f %f %f %s %s %s \r',...
    'headerlines',1,'delimiter','\t');

currd=pwd;
cd(locdir);

count=1;
for ii=1:length(in)
    ii
    qq=in(ii).cell;
    ss=strcmpi(in(ii).name,fname);
    if exist(in(ii).name) * (qq>0)
        x=load(in(ii).name);
        if (isfield(x.strct,'dRatio'))
            x.strct.dRatio1 = x.strct.dRatio;
        end
        if qq<=size(x.strct.dRatio1,1) % Variables for the current ROI are stored
            out(count).stimcode=stimcode{ss};
                out(count).quality=quality(ss);
                out(count).driver=driver{ss};
                out(count).layer=layer(ss);
                out(count).wavelength=wavelength(ss);
                out(count).flyID=flyID(ss);
                out(count).name=in(ii).name;
                out(count).cell=in(ii).cell;
                try
                    out(count).zlayer = x.strct.layer;
                catch
                    disp(fprintf('This pData: %s  does not have (z)layer information', out(count).name));
                end
                out(count).xml=x.strct.xml;
                out(count).ratio=x.strct.dRatio1(qq,:);
                out(count).stim = x.strct.fstimval;
                out(count).raw_stim = x.strct.ch3;
                out(count).avrstimval = x.strct.avrstimval;
                out(count).frame_nums = x.strct.frame_nums;
                fps=x.strct.xml.framerate;
                out(count).t=[1:length(out(count).ratio)]/fps; %Here we generate the time vector:
frames / framerate
                out(count).it=[0.5/fps:0.1:(length(out(count).ratio)+0.5)/fps]; %New time vector for
interpolation at 10Hz (0.1), starts and ends with 0.5/fps shift

                %Nearest neighbor interpolation for the stimulus (should maintain discrete values) of
.stim (at 10Hz)
                out(count).istim=interp1(out(count).t,out(count).stim,out(count).it,'nearest','extrap');


                %Linear interpolation for the response/signal (ratio)
                out(count).iratio=interp1(out(count).t,out(count).ratio,out(count).it,'linear','extrap');



                % Added stimpos and centers  interpl is based on the length of out.t
                if(isfield(x.strct,'fstimpos1'))

out(count).ifstimpos1=interp1(out(count).t,x.strct.fstimpos1,out(count).it,'nearest','extrap');
```

```
out(count).ifstimpos2=interp1(out(count).t,x.strct.fstimpos2,out(count).it,'nearest','extrap');

                end

                if(isfield(x.strct,'cell_nums'))
                    out(count).ref_cell_num = x.strct.cell_nums(qq);
                end
                count=count+1;
        else
            disp([in(ii).name ': cell out of bounds...']);
        end
    else
        disp([in(ii).name ' unfound -- skipping']);
    end
end

cd(currd);
```

Fff analysis-related script

Script name: BASELINE_aggregate_fffall_means10Hz_BleedThruFix_elife.m

```
% This function calculates the baseline for dF/F calculation

function out = BASELINE_aggregate_fffall_means10Hz_BleedThruFix_elife(in,baseline)

baseline = baseline; % It determines how dF/F is beine calculated


dxi_all = zeros(length(in),1);
%determine the epoch length
for ii=1:length(in)
        s=in(ii).istim;
        xi=find(diff(s(1:end))<0)+1;
        dxi = mean(diff(xi));
        dxi_all(ii,:)=dxi;
end
epochlength = round(mean(dxi_all));
dur = epochlength*1.3;




rats=zeros(length(in),dur);
stims=rats;

mr=zeros(length(in),dur);
ms=zeros(length(in),dur);
name = cell(length(in),1);
driver = cell(length(in),1);

for ii=1:length(in)
        s=in(ii).istim;
        xi=find(diff(s(1:end-dur))<0)-1;
        iratio = in(ii).iratio;
```

```
    for jj=1:length(xi) %Number of repetitions.
        temp = iratio(xi(jj):xi(jj)+dur-1); %start of repetition + duration
        if strcmp(baseline, 'ON')
            F = 'epochlength/2+(4*epochlength/10):epochlength-5';
        elseif strcmp(baseline, 'OFF')
            F = 'epochlength/2-epochlength/10:epochlength/2-epochlength/20';
        else
            F = '1:dur';
        end


        temp = temp./mean(temp(eval(F)))-1;
        mr(jj,:)=temp;

        temp=in(ii).istim(xi(jj):xi(jj)+dur-1);
        ms(jj,:)=temp;
    end
    rats(ii,:)=mean(mr(1:length(xi),:),1); %averaging over stimulus repititions
    stims(ii,:)=mean(ms(1:length(xi),:),1);
    name{ii}=in(ii).name;
    driver{ii}=in(ii).driver;
end
neuron = [in.cell];
flyID = [in.flyID];
if isfield(in,'zlayer')
    zlayer = [in.zlayer];
    out.zlayer = zlayer;
end


out.rats=rats;
out.stims=stims;
out.neuron=neuron;
out.driver = driver;
out.flyID=flyID;
out.name=name;
```

Fff analysis-related script

Script name: mean_cat_elife.m

```
% This function computes mean and sem of a along dimension dim, grouped first by category
(cats)

function [x,m,e]=mean_cat_elife(a,dim,cats)

if dim == 2
    a=a';
end

fnan=find(all(~isnan(a),2));
a=a(fnan,:);
cats=cats(fnan);

u=unique(cats);
```

```
x=zeros(length(u),size(a,2));
for ii=1:length(u)
    x(ii,:)=mean(a(find(cats==u(ii)),:),1);
end

m=mean(x,1);
e=std(x,[],1)/sqrt(length(u));
```

Fff analysis-related script

Script name: fff_stimulus_response_filters_elife.m

```
%This function contains all the different filters to apply for 5sec ON OFF
%fff. The following filters can be applied or not. User choice:
%Pearson correlation between stimulus and response
%STD filter
%ON or OFF response filter
%dF/F absolut-response filter

% Author: Sebastian Molina-Obando

function
[x,m,e,final_mat,final_IDs,inds,inds_responding,inds_notresponding,cur_mat_notresp,inds_pea
kThreshold,ROI_max]...
    = fff_stimulus_response_filters_elife...

(cur_mat,cur_stims,cur_IDs,NEURON_POLARITY,CorrelationValue,ApplyThreshold_ON,Thres
hold_ON,Threshold_OFF,ApplyThreshold_OFF,...
    ApplyPeakThreshold,Threshold_Peak,NORMALIZE_ROI,Apply_STD_filter)



%% Preparing variables
Q = corr(mean(cur_stims)',cur_mat'); % Correlation vector
inds_peakThreshold = [];
ROI_max = [];

%% Applying response filters

    %For ON:
    %It is only applied for defined ON-responding (ON-polarity) neurons.
    %The ON threshold is a user-defined scalar that is multiply by the STD
    %of each response-trace at the end of the ON-Step, where no
    %contrast change is present. This threshold value is compared with the
    %response maximun after the ON-Step stimulation. It means that either
    %noisy or not ON-responding cells (ROIs) are filter out.

if ApplyThreshold_ON == true && strcmp(NEURON_POLARITY, 'ON')

    % Positive correlation with stimulus. Only ON-responding cells will
    % have a positive correlation
    inds = find(Q>CorrelationValue);
    cur_mat_pos = cur_mat(inds,:);  % ROIs responses
    cur_IDs_pos = cur_IDs(inds);    % Fly IDs for ROIs

    %Preparing matrices for data analysis
```

```
    cur_mat_resp = cur_mat_pos;
    cur_mat_notresp = cur_mat_pos;

    %Creating the ON-response filter
    maxPeak = max(cur_mat_resp(:,45:60),[],2);
    meanTraces = mean(cur_mat_resp(:,35:50),2);
    stdTraces = std(cur_mat_resp(:,35:50),[],2);
    filter= meanTraces+(Threshold_ON*stdTraces);

    %Creating the responding and non-responding cells vectors
    if Apply_STD_filter
        std_inds = std(cur_mat_resp(:,25:45),[],2);
        inds_responding = find(maxPeak>filter & std_inds < 0.2);
        inds_notresponding = find(maxPeak<filter & std_inds > 0.2);
    else
        inds_responding = find(maxPeak>filter);
        inds_notresponding = find(maxPeak<filter);
    end

    %Applying the filters and gettin the responding and non-responding
    %matrices
    cur_mat_resp = cur_mat_resp(inds_responding,:);
    cur_mat_notresp = cur_mat_notresp(inds_notresponding,:);
    cur_mat_pos = cur_mat_resp;
    cur_IDs_pos = cur_IDs_pos (inds_responding);



    % dF/F absolut-response filter
    if ApplyPeakThreshold == true
        cur_mat_peakThreshold = cur_mat_pos;
        maxPeak = max(cur_mat_peakThreshold(:,45:60),[],2);
        inds_peakThreshold = find(maxPeak>Threshold_Peak);
        cur_mat_peakThreshold = cur_mat_peakThreshold(inds_peakThreshold,:);
        cur_mat_pos = cur_mat_peakThreshold;
        cur_IDs_pos = cur_IDs_pos (inds_peakThreshold);
    end

    %For OFF:
    %It is only applied for defined OFF-responding (OFF-polarity) neurons.
    %The OFF threshold is a user-defined scalar that is multiply by the STD
    %of each response-trace at the end of the OFF-Step, where no
    %contrast change is present. This threshold value is compared with the
    %response maximun after the OFF-Step stimulation. It means that either
    %noisy or not OFF-responding cells (ROIs) are filter out.

elseif ApplyThreshold_OFF == true && strcmp(NEURON_POLARITY, 'OFF')

    % Negative correlation with stimulus. Only OFF-responding cells will
    % have a negative correlation.
    inds = find(Q<-CorrelationValue);
    cur_mat_neg = cur_mat(inds,:);  % ROIs responses
    cur_IDs_neg = cur_IDs(inds);    % Fly IDs for ROIs

    %Preparing matrices for data analysis
    cur_mat_resp = cur_mat_neg;
    cur_mat_notresp = cur_mat_neg;

    %Creating the OFF-response filter
```

```
    maxPeak = max(cur_mat_resp(:,95:110),[],2);
    meanTraces = mean(cur_mat_resp(:,85:100),2);
    stdTraces = std(cur_mat_resp(:,85:100),[],2);
    filter= meanTraces+(Threshold_OFF*stdTraces);

    %Creating the responding and non-responding cells vectors
    inds_responding = find(maxPeak>filter);
    inds_notresponding = find(maxPeak<filter);

    %Applying the filters and gettin the responding and non-responding
    %matrices
    cur_mat_resp = cur_mat_resp(inds_responding,:);
    cur_mat_notresp = cur_mat_notresp(inds_notresponding,:);
    cur_mat_neg = cur_mat_resp;
    cur_IDs_neg = cur_IDs_neg (inds_responding);

    % dF/F absolut-response filter
    if ApplyPeakThreshold == true
        cur_mat_peakThreshold = cur_mat_neg;
        maxPeak = max(cur_mat_peakThreshold(:,95:110),[],2);
        inds_peakThreshold = find(maxPeak>Threshold_Peak);
        cur_mat_peakThreshold = cur_mat_peakThreshold(inds_peakThreshold,:);
        cur_mat_neg = cur_mat_peakThreshold;
        cur_IDs_neg = cur_IDs_neg (inds_peakThreshold);
    end

else
    % Else, no ON or OFF response-filters are used, only correlation filter
    % is used to take the expected(known)response-polarity ROIs.

    if strcmp(NEURON_POLARITY, 'ON')
        inds = find(Q>CorrelationValue);
        cur_mat_pos = cur_mat(inds,:);
        cur_IDs_pos = cur_IDs(inds);
        inds_responding = inds;
        inds_notresponding = find(Q<CorrelationValue);
        inds_peakThreshold =[];
        cur_mat_notresp = cur_mat(inds_notresponding,:);

    elseif strcmp(NEURON_POLARITY, 'OFF')
        inds = find(Q<-CorrelationValue);
        cur_mat_neg = cur_mat(inds,:);
        cur_IDs_neg = cur_IDs(inds);
        inds_responding = inds;
        inds_notresponding =  find(Q>-CorrelationValue);
        inds_peakThreshold =[];
        cur_mat_notresp = cur_mat(inds_notresponding,:);
    else

      % Else, the response-polarity is not know or if more than one polarity is
      % expected, the correlation analysis is not applied and all ROIs are considered.
        cur_mat_resp = cur_mat;
        inds= 1:size(cur_mat,1);
        inds_responding = inds;
        inds_notresponding =  [];
        inds_peakThreshold =[];
        cur_mat_notresp = [];
    end
end
```

```
%% Calculating response means and errors

%Calculating means across flies. x  > mean across ROIS per fly,
%m  > mean across fly means, e > sem across fly means

if strcmp(NEURON_POLARITY, 'OFF')

    if NORMALIZE_ROI
        ROI_max = max(cur_mat_neg,[],2)*ones(1,length(cur_mat_neg(1,:)));
        cur_mat_neg_norm = cur_mat_neg./ROI_max; % normalized by dividing by the max
responses for each ROI
        cur_mat_neg = cur_mat_neg_norm;
    end

        [x,m,e] = mean_cat_full(cur_mat_neg,1,cur_IDs_neg);
        final_mat = cur_mat_neg;
        final_IDs = cur_IDs_neg;

elseif strcmp(NEURON_POLARITY, 'ON')

    if NORMALIZE_ROI
        ROI_max = max(cur_mat_pos,[],2)*ones(1,length(cur_mat_pos(1,:)));
        cur_mat_pos_norm = cur_mat_pos./ROI_max; % normalized by dividing by the max
responses for each ROI
        cur_mat_pos = cur_mat_pos_norm;
    end

        [x,m,e] = mean_cat_full(cur_mat_pos,1,cur_IDs_pos);
        final_mat = cur_mat_pos;
        final_IDs = cur_IDs_pos;

else

    if NORMALIZE_ROI
        ROI_max = max(cur_mat,[],2)*ones(1,length(cur_mat(1,:)));
        cur_mat_norm = cur_mat./ROI_max; % normalized by dividing by the max responses for
each ROI
        cur_mat = cur_mat_norm;
    end
    [x,m,e] = mean_cat_full(cur_mat,1,cur_IDs);
    final_mat = cur_mat;
    final_IDs = cur_IDs;

end
```

Fff analysis-related script

Script name: niceaxes.m

```
%It makes current axes nicer

set(gca,'fontname','times','fontsize',14);
ax=findall(gca,'Type','line');
for i=1:length(ax)
    set(ax(i),'linewidth',2);
```

```
end
ax=findall(gcf,'Type','text');
for i=1:length(ax)
    set(ax(i),'fontname','times','fontsize',14);
end
```

Fff analysis-related script

Script name: plot_err_patch_v2.m

```
% This function plots the sem shade around the mean.

function g=plot_err_patch_v2(x,y,e,col1,col2,style)

x=x(:)'; %Inverts to column vector
y=y(:)';
e=e(:)';

ye1=y+e;
ye2=y-e; ye2=ye2(end:-1:1);
ye=[ye1,ye2];
xe=[x,x(end:-1:1)];

hold on;
h=patch(xe,ye,col2,'linestyle','none');
set(h,'FaceAlpha',0.7);
if(nargin==6)
    g=plot(x,y,'color',col1,'linewidth',2,'linestyle',style);
else
    g=plot(x,y,'color',col1,'linewidth',2);
end
```

Full field flash plot and statistical analysis

Script name: Trace_per_Category_and_Genotype_elife.m

Sub-functions needed:

database_select_samples_elife.m

load_experiment_conditions.m

niceaxes.m

plot_err_patch_v2.m

```
% Plotting and Stadistical analysis script for ON / OFF full field flashes (fff).
% It loads and analyze all files for given experimental conditions in a txt
% file (automatized mode)or for an specific experiment (non-automatized
% mode)

% Author: Sebastian Molina-Obando

clc;
clear all;
close all;



%% GENERAL CONSTANTS
% The user needs to define this.
Automatized = true; %"True" if a txt file for the automatized mode is used. Otherwise, write
"false".
savingData = true; %"True" for saving data (variables and plots) in a folder in your computer.
Otherwise, write "false".
save_in_folder = 'C:\MATLAB_FOLDER\2p\2p_pData\5secfff\eLife\ExperimentalData\'; % Write
the path of the folder
load_data_from = 'C:\MATLAB_FOLDER\2p\2p_pData\5secfff\eLife\GenotypesData\'; % Write
the path of the folder
BaselineShift = true; %"True" for plotting baseline at 0. Otherwise, write "false".
NeuronType = 'ON'; %Choose between 'ON','OFF' based on the neuron response polarity.
OutlierFilter = false; % "True" for filetering out the outliers in the Fly matrix. Otherwise, write
"false".

%% CONSTANTS for non-Automatized mode
% The user need to define this.
NEURONCLASS = 'Tm3'; % for adjusting plot y limits and adding text on the figure
NORMALIZE_ROI = false; % "True" to plot existing ROI-normalized data. Otherwise, write
"false".
NORMALIZE_FLY = true; % "True" to plot existing Fly-normalized data. Otherwise, write "false".
COLORS = 'greenOrange'; % 'blueOrange', 'greenRed', 'blueRed'...
NUMBER_OF_VARIABLES = 1; % Define the existing number of variables to plot (Step,
Plateau, Integral...)
NUMBER_OF_GROUPS = 2; % Define the desired number of groups (genotypes) to be plotted
together
LAYER = 'M1'; % Select the layer: 'M1', 'M5', 'M9', 'Ax', 'De' ...
EXPERIMENT = 'Tm3 MPEP'; % Define the name of the experiment. There will be a specific
folder created with the data
CONDITION = '100uM PTX'; % Define the name of the experimental condition. There will be a
specific folder created with the data
pairedData = false; % "True" if your groups (genotypes) are paired. Otherwise, write "false".
Statistics = 'T-TEST'; % Choose the statistical test to perform: 'ANOVA1', 'ANOVA2', 'ANOVAn',
'T-TEST'
```

```
Correction = 'None';% Choose the type of post-hoc correction: 'Bonferroni','Tukey', ...

%% Organizing variables
numberOfgroups = NUMBER_OF_GROUPS;
numberOfvariables = NUMBER_OF_VARIABLES;
gen_str = cell(numberOfgroups);
variable_str = cell(numberOfvariables);
DataNames = {};

%Asking for user input in non-automatized mode
if Automatized == false

   for i = 1:numberOfgroups
      prompt = 'Name(genotype): ';
      GENOTYPE_STRING = input(prompt,'s');
      gen_str{i} = GENOTYPE_STRING;

   end

   for i = 1:numberOfvariables
      prompt = 'Name(variable to measure): ';
      VARIABLE_STRING = input(prompt,'s');
      variable_str{i} = VARIABLE_STRING;
   end
end

%% Selecting samples from Database
% add the path of needed functions
% addpath(genpath('C:\Users\s.molinaobando\MATLAB\TP_code_Seb'))
% addpath(genpath('C:\MATLAB_FOLDER\TP_code_Seb'));
database_select_samples_elife; %Here all the different experimental conditions are added (E.g.
genotype, stimulus presented, experiment number, quality of recording, DRUG...)

%% Automatized Analysis: data selection

stopForLoop = false;
   for aut_i = 1:length(EXPERIMENT)
      if stopForLoop
         break
      end
      if Automatized
         close all

[EXPERIMENT,CONDITION,NEURONCLASS,NORMALIZE_ROI,NORMALIZE_FLY,LAYER,C
OLORS,pairedData,...

NUMBER_OF_VARIABLES,NUMBER_OF_GROUPS,variable_str,gen_str,DataNames,Statistic
s,Correction]...

=load_experiment_conditions(Automatized,EXPERIMENT,CONDITION,NEURONCLASS,NOR
MALIZE_ROI,...

NORMALIZE_FLY,LAYER,COLORS,pairedData,NUMBER_OF_VARIABLES,NUMBER_OF_G
ROUPS,variable_str,gen_str,DataNames,Statistics,Correction);


         EXPERIMENT = char(EXPERIMENT(aut_i));
         CONDITION = char(CONDITION(aut_i));
```

```
            NEURONCLASS = char(NEURONCLASS(aut_i));
            NORMALIZE_FLY = NORMALIZE_FLY(aut_i);
            NORMALIZE_ROI = NORMALIZE_ROI(aut_i);
            COLORS = char(COLORS(aut_i));
            LAYER = char(LAYER(aut_i));
            variable_str = char(variable_str(aut_i));
            gen_str = char(gen_str(aut_i));
            numberOfvariables = NUMBER_OF_VARIABLES(aut_i);
            numberOfgroups = NUMBER_OF_GROUPS(aut_i);
            AllDataNames = DataNames;
            Original_variable_str = variable_str;
            Original_gen_str = gen_str;
            Statistics = char(Statistics(aut_i));
            Correction = char(Correction(aut_i));

        else
            stopForLoop = true;

        end



%% COLORS
% Color combinations (mean, sem) for 2 or 3 groups
            switch COLORS

                case 'greenRed'
                    colorss = {[44/256 162/256 95/256] ; [153/256 216/256 201/256];[222/256 45/256
38/256];...
                        [252/256 146/256 114/256]}; %greenRed
                case 'greenOrange'
                    colorss = {[44/256 162/256 95/256] ; [153/256 216/256 201/256];[217/256 95/256
14/256];...
                        [254/256 196/256 79/256]}; %greenOrange

                case 'blueRed'
                    colorss = {[49/256 130/256 189/256] ; [158/256 202/256 225/256];[222/256 45/256
38/256];...
                        [252/256 146/256 114/256]}; %blueRed

                case 'blueOrange'
                    colorss = {[49/256 130/256 189/256] ; [158/256 202/256 225/256];[217/256 95/256
14/256];...
                        [254/256 196/256 79/256]}; %blueOrange

                case 'greyLightgreyRed'
                    colorss = {[99/256 99/256 99/256] ; [189/256 189/256 189/256];[189/256 189/256
189/256]...
                        ;[240/256 240/256 240/256];[222/256 45/256 38/256];[252/256 146/256
114/256]}; %greyLightgreyRed

                case 'greyOrangeRed'
                    colorss = {[99/256 99/256 99/256] ; [189/256 189/256 189/256];[217/256 95/256
14/256]...
                        ;[254/256 196/256 79/256];[222/256 45/256 38/256];[252/256 146/256
114/256]}; %greyOrangeRed

            end
```

```
%% Loading Data and organizing variables in matrices

        for var = 1:numberOfvariables

            %Initializing variables
            allDataCell= cell(numberOfgroups,1);
            m_all_mat =[];
            e_all_mat =[];
            numOfflies_mat =[];
            numOfROIs_mat =[];
            clear GenotypeSampleInfo;

            for i = 1:numberOfgroups

                if var == 1
                    if Automatized
                        DataNames = AllDataNames;
                        DataNames = char(DataNames(aut_i));
                        DataNames = strsplit(DataNames, ',');

                    else

                    prompt = 'select the Data (name of the folder contaning the data): ';
                    Data = input(prompt,'s');
                    DataNames{i} = Data;
                    end
                end

                % Loading previsouly-analysed data
                if NORMALIZE_ROI
                    Data = char(DataNames(i));
                    load([load_data_from,'NORMALIZED_by_ROI\',Data,'\',LAYER], 'final_mat',
'final_IDs','FlyOffStep','FlyOnStep','FlyOffPlateau','FlyOnPlateau','FlyOffPeak','FlyOnPeak','Stim
ulus_trace');
                else
                    Data = char(DataNames(i));
                    load([load_data_from,Data,'\',LAYER],'x_final','m_final','e_final',...
                        'final_mat',
'final_IDs','FlyOffIntegral','FlyOnIntegral','FlyOffIntegral_norm','FlyOnIntegral_norm','FlyOffStep','
FlyOnStep','FlyOffStep_norm','FlyOnStep_norm','FlyOffPlateau',...

'FlyOnPlateau','FlyOffPlateau_norm','FlyOnPlateau_norm','FlyOffPeak','FlyOnPeak','FlyOffPeak
_norm',...

'FlyOnPeak_norm','FlyOffPeakAbovePlateau','FlyOnPeakAbovePlateau','FlyOffPeakAbovePlate
au_norm','FlyOnPeakAbovePlateau_norm','Stimulus_trace');
                end

                m_all_mat(i,:) = m_final;
                e_all_mat(i,:) = e_final;
                numOfflies_mat(i) = size(x_final,1);
                numOfROIs_mat(i) = size(final_mat,1);
                iRATE = 10;
                cur_t = [1:size(final_mat,2)]/iRATE;

                % Creating data matrices for future plots and analysis
                if Automatized
```

```
            variable_str = Original_variable_str;
            variable_str = strsplit(variable_str,',');
        end

        if NORMALIZE_FLY
            FlyOnVariable = eval(['FlyOn', char(variable_str(var)),'_norm']);
            FlyOffVariable = eval(['FlyOff', char(variable_str(var)),'_norm']);
        else
            FlyOnVariable = eval(['FlyOn', char(variable_str(var))]);
            FlyOffVariable = eval(['FlyOff', char(variable_str(var))]);
        end

        % Filtering Data Outliers by Fly
        if OutlierFilter
            outlier_filter_On = ~isoutlier(FlyOnVariable);
            FlyOnVariable = FlyOnVariable(outlier_filter_On);
            outlier_filter_Off = ~isoutlier(FlyOffVariable);
            FlyOffVariable = FlyOffVariable(outlier_filter_Off);
        end

        %Naming variables accoring to LAYERs being analyzed
        if(strcmp(LAYER,'Ax'))
                x.FlyOffVariable_Ax=FlyOffVariable;
                x.FlyOnVariable_Ax=FlyOnVariable;

        elseif(strcmp(LAYER,'M1'))
                x.FlyOffVariable_M1=FlyOffVariable;
                x.FlyOnVariable_M1=FlyOnVariable;

        elseif(strcmp(LAYER,'M5'))
                x.FlyOffVariable_M5=FlyOffVariable;
                x.FlyOnVariable_M5=FlyOnVariable;

        elseif(strcmp(LAYER,'M9'))
                x.FlyOffVariable_M9=FlyOffVariable;
                x.FlyOnVariable_M9=FlyOnVariable;

        else
                    Disp('Not such a CATEGORY implemented yet');
        end

            allDataCell{i} = x;
    end


%% Plotting mean across flies
    f1=figure(1);
    hold on
    a= 0; % Initialyzing variable

    for i = 1:numberOfgroups
        cm=colormap('lines');
        if BaselineShift
            switch NeuronType
                case 'ON'
                    m_all_mat(i,:) = m_all_mat(i,:) - mean(m_all_mat(i,40:50));
                case 'OFF'
                    m_all_mat(i,:) = m_all_mat(i,:) - mean(m_all_mat(i,90:100));
```

```
                end
            end
            try
                h =
plot_err_patch_v2(cur_t,m_all_mat(i,:),e_all_mat(i,:),colorss{i+a},colorss{i+1+a});
            catch
                % In the case of plotting together more groups than
                % colors available in the variable "colorss"
                h = plot_err_patch_v2(cur_t,m_all_mat(i,:),e_all_mat(i,:),colorss{1},colorss{3});
            end

            if Automatized
                gen_str = Original_gen_str;
                gen_str = strsplit(gen_str,',');
            end

            h.DisplayName = sprintf([gen_str{i} ', N = %d ( %d
)'],numOfflies_mat(i),numOfROIs_mat(i));
            GenotypeSampleInfo{i} = sprintf([gen_str{i} ', N = %d ( %d
)'],numOfflies_mat(i),numOfROIs_mat(i));
            handle(i).handle = h;

            %Adjusting the plot and adding information
            switch NEURONCLASS
                case 'Tm3'
                    ylim ([-0.8 5])
                    plot(cur_t, (round(mean(Stimulus_trace))*0.1)+1.8);
                    if i == numberOfgroups
                        text(8,1.5,GenotypeSampleInfo');
                    end

                case 'Mi1'
                    ylim ([-0.8 4])
                    plot(cur_t, (round(mean(Stimulus_trace))*0.1)+1.8);
                    if i == numberOfgroups
                        text(8,1.5,GenotypeSampleInfo');
                    end

                case 'T4_T5'
                    ylim ([-0.5 3]);
                    plot(cur_t, (round(mean(Stimulus_trace))*0.2)+2.5);
                    if i == numberOfgroups
                        text(8,0.5,GenotypeSampleInfo');
                    end

                case 'T4_T5_small'
                    ylim ([-0.5 0.8]);
                    plot(cur_t, (round(mean(Stimulus_trace))*0.1)+0.6);
                    if i == numberOfgroups
                        text(8,0.5,GenotypeSampleInfo');
                    end
                case 'iGluSnFR'
                    ylim([-0.4 0.4]);
                    plot(cur_t, (round(mean(Stimulus_trace))*0.1)+0.3);
                    if i == numberOfgroups
                        text(6,0.3,GenotypeSampleInfo');
                    end
                case 'L1'
                    ylim([-0.8 0.8]);
```

```
                plot(cur_t, (round(mean(Stimulus_trace))*0.1)+0.5);
                if i == numberOfgroups
                    text(6,0.3,GenotypeSampleInfo');
                end
            end

            set(gcf,'Color','w');
            niceaxes;
            a = a +1;
        end

        title([EXPERIMENT,' Mean by fly, LAYER:',LAYER]);
        ylabel('dF/F');
        xlabel ('s');

        if numberOfgroups == 1
            figure(2);
            plot(cur_t,x_all)
            title('individual fly means');
        end

%% Plotting mean across flies (for normalized-by-fly vairable)

        if NORMALIZE_FLY && numberOfgroups < 8
            f3=figure(3);
            hold on
            a= 0;
            for i = 1:numberOfgroups

                Data = DataNames{i};
                %Loading normalized data
                load([load_data_from,Data,'\',LAYER], 'final_mat',
'final_IDs','Stimulus_trace','x_final_norm','e_final_norm');

                if BaselineShift
                    m_x_final_norm = mean(x_final_norm);
                    x_final_norm = x_final_norm - mean(m_x_final_norm(40:50));
                end
                try
                    h3 =
plot_err_patch_v2(cur_t,mean(x_final_norm),e_final_norm,colorss{i+a},colorss{i+1+a});
                catch
                    % In the case of plotting together more groups than
                    % colors available in the variable "colorss"
                    h3 =
plot_err_patch_v2(cur_t,mean(x_final_norm),e_final_norm,colorss{1},colorss{3});
                end


                axis('tight');
                a= a+1;
            end

            % Adjusting the plot and adding information
            ylim ([-0.8 1.5])
            plot(cur_t, (round(mean(Stimulus_trace))*0.1)+1);

            text(8,0.7,GenotypeSampleInfo');
```

```
            string1 =([EXPERIMENT,' LAYER: ',LAYER]);
            title({string1;'Mean by Fly, Normalized by Fly to Control'});
        end

%% Bar plot: Data Analysis and arrangement
        gen_str = gen_str';
        if Automatized
            gen_str = gen_str';
        end

        Variables_str = {'ON'; 'OFF'};
        for i = 1:numberOfgroups
            gen_str2(i) = {gen_str{1,i}};
        end

        % Assigning Variables
        meanOnVariableMatrix = cell(numberOfgroups,1);
        stdOnVariableMatrix = cell(numberOfgroups,1);
        semOnVariableMatrix = cell(numberOfgroups,1);

        meanOffVariableMatrix = cell(numberOfgroups,1);
        stdOffVariableMatrix = cell(numberOfgroups,1);
        semOffVariableMatrix = cell(numberOfgroups,1);

        for j = 1:numberOfgroups
                OnVariableMatrix{j} = eval(['allDataCell{j}.FlyOnVariable_',LAYER]);
                meanOnVariableMatrix{j}  =
mean(eval(['allDataCell{j}.FlyOnVariable_',LAYER]));
                stdOnVariableMatrix{j} = std(eval(['allDataCell{j}.FlyOnVariable_',LAYER]));
                semOnVariableMatrix{j} =
std(eval(['allDataCell{j}.FlyOnVariable_',LAYER]))/sqrt(length(eval(['allDataCell{j}.FlyOnVariable
_',LAYER])));

                OffVariableMatrix{j} = eval(['allDataCell{j}.FlyOffVariable_',LAYER]);
                meanOffVariableMatrix{j}  =
mean(eval(['allDataCell{j}.FlyOffVariable_',LAYER]));
                stdOffVariableMatrix{j} = std(eval(['allDataCell{j}.FlyOffVariable_',LAYER]));
                semOffVariableMatrix{j} =
std(eval(['allDataCell{j}.FlyOffVariable_',LAYER]))/sqrt(length(eval(['allDataCell{j}.FlyOffVariable
_',LAYER])));
        end


        On_y = meanOnVariableMatrix;
        Off_y= meanOffVariableMatrix;
        On_y = cell2mat(On_y)';
        Off_y = cell2mat(Off_y)';

        On_sem = semOnVariableMatrix;
        Off_sem= semOffVariableMatrix;
        On_sem = cell2mat(On_sem)';
        Off_sem = cell2mat(Off_sem)';

%% Statistical Analysis

        if numberOfgroups == 2

        %ON Variable
```

```
            disp('>>>>>>>>>>> Statistical Analysis for ON Variable: ');

            X = OnVariableMatrix{1};
            Y = OnVariableMatrix{2};

            %Normality test
            try
                [h,p,kstat,critval] = lillietest(X,'Alpha',0.01); display(['Is X normal distributed?'
,fprintf('For X, h= %d, p=%f',h,p)]); %Normality test
                [h,p,kstat,critval] = lillietest(Y,'Alpha',0.01); display(['Is Y normal
distributed?',fprintf('For Y, h= %d, p=%f',h,p)]);% Normality test
            catch
                disp('Error in Normality test');
            end

            % Performing T-TEST
            if pairedData
                try
                    [h,p1] = ttest(X,Y); display(fprintf('For X vs Y, h= %d, p=%f',h,p1)); %Ttest
                    OnVariablePvalue = sprintf('Pvalue ON: %f ', p1);
                catch
                    [h,p1] = ttest2(X,Y); display(fprintf('For X vs Y, h= %d, p=%f',h,p1)); %Ttest
                    OnVariablePvalue = sprintf('Pvalue ON: %f ', p1);
                    display('DIFFERENT SAMPLE SIZE AMONG GROUPS');
                end
            else
                [h,p1] = ttest2(X,Y); display(fprintf('For X vs Y, h= %d, p=%f',h,p1)); %Ttest
                OnVariablePvalue = sprintf('Pvalue ON: %f ', p1);
            end

        %OFF Variable
            disp('>>>>>>>>>>> Statistical Analysis for OFF Variable: ');

            X = OffVariableMatrix{1};
            Y = OffVariableMatrix{2};

            %Normality test
            try
                [h,p,kstat,critval] = lillietest(X,'Alpha',0.01); display(['Is X normal distributed?'
,fprintf('For X, h= %d, p=%f',h,p)]); %Normality test
                [h,p,kstat,critval] = lillietest(Y,'Alpha',0.01); display(['Is Y normal
distributed?',fprintf('For Y, h= %d, p=%f',h,p)]);% Normality test
            catch
                 disp('Error in Normality test');
            end

            % Performing T-TEST
            if pairedData
                try
                    [h,p1] = ttest(X,Y); display(fprintf('For X vs Y, h= %d, p=%f',h,p1)); %Ttest
                    OffVariablePvalue = sprintf('Pvalue OFF: %f ', p1);
                catch
                    [h,p1] = ttest2(X,Y); display(fprintf('For X vs Y, h= %d, p=%f',h,p1)); %Ttest
                    OffVariablePvalue = sprintf('Pvalue OFF: %f ', p1);
                    display('DIFFERENT SAMPLE SIZE AMONG GROUPS, data is not paired');
                end
            else
                [h,p1] = ttest2(X,Y); display(fprintf('For X vs Y, h= %d, p=%f',h,p1)); %Ttest
                OffVariablePvalue = sprintf('Pvalue OFF: %f ', p1);
```

```
            end

        elseif numberOfgroups > 2
        %ON Variable
            disp('>>>>>>>>>>>> Statistical Analysis for ON Variable: ');
            h_all_OnVariable = {};
            On_p_values=[];
            h_all_OnVariable{1} = 'Pvalues ON Variable';
            X = OnVariableMatrix{1};
            X_name = gen_str{1};

            %Normality test for first group
            try
                [h,p,kstat,critval] = lillietest(X,'Alpha',0.01);
            catch
                h = ' Not computed';
                disp(['>>>>> Error in Normality test
',EXPERIMENT,'_',LAYER,'_',char(variable_str(var))]);
            end

            if h == 1 %if h=1, the null hypothesis ("the data is normally distributed") IS rejected
                ANSWER1 = ' NO';
                display(['Normal distributed?' ,fprintf('For %s, h= %d, p=%f',X_name,h,p),
ANSWER1]); %Normality test
            elseif strcmp(h,' Not computed')
                ANSWER1 = h;
                display(['Normal distributed?',ANSWER1,]); %Normality test
            elseif h == 0
                ANSWER1 = ' YES';
                display(['Normal distributed?' ,fprintf('For %s, h= %d, p=%f',X_name,h,p),
ANSWER1]); %Normality test
            end

            % Normality test for the other groups and T-TEST
            for s = 2:numberOfgroups
                Y = OnVariableMatrix{s};
                Y_name = gen_str{s};
                try
                    [h,p,kstat,critval] = lillietest(Y,'Alpha',0.01);
                catch
                    h = ' Not computed';
                    disp(['>>>>> Error in Normality test
',EXPERIMENT,'_',LAYER,'_',char(variable_str(var))]);
                end

                if h == 1 %if h=1, the null hypothesis ("the data is normally distributed") IS
rejected
                    ANSWER1 = ' NO';
                    display(['Normal distributed?' ,fprintf('For %s, h= %d, p=%f',Y_name,h,p),
ANSWER1]); %Normality test
                elseif strcmp(h,' Not computed')
                    ANSWER1 = h;
                    display(['Normal distributed?',ANSWER1,]); %Normality test
                elseif h == 0
                    ANSWER1 = ' YES';
                    display(['Normal distributed?' ,fprintf('For %s, h= %d, p=%f',Y_name,h,p),
ANSWER1]); %Normality test
                end
```

```
                [h,p1] = ttest2(X,Y); display(fprintf('T-TEST. For %s vs %s, h= %d,
p=%f',X_name,Y_name,h,p1)); %Ttest
                OnVariablePvalue = sprintf('Pvalue ON: %f ', p1);
                h_all_OnVariable{s} =p1;
                On_p_values(s-1)= p1;
            end

            % For Anova. Variable's matrix organization and tests

                %Initializing variables
                Y_all= [];
                groups = {};
                %Organizing matrices
                for s = 1:numberOfgroups
                    if strcmp(Statistics, 'ANOVA1')
                        Y_all = vertcat(Y_all ,OnVariableMatrix{s}); %Organizing y vector for Anova
                        for g =1:length(OnVariableMatrix{s})
                            groups = [groups gen_str(s)];
                        end
                    elseif strcmp(Statistics, 'ANOVAn')
                        Y_all = vertcat(Y_all ,OnVariableMatrix{s}); %Organizing y vector for
Anovan

                        for g =1:length(OnVariableMatrix{s})
                            groups = [groups gen_str(s)];
                        end

                    elseif strcmp(Statistics, 'ANOVA2')
                        Y_all(s) = mean(OnVariableMatrix{s});
                    end
                end

                % Performing tests
                if strcmp(Statistics, 'ANOVA1') %One-way Anova
                    [p,tb1,stats] = anova1(Y_all,groups);
                    f2= figure(2); title([EXPERIMENT,variable_str(var),' ON Response ']);
                    f4= figure(4); title([EXPERIMENT,variable_str(var),' ON Response ']);
                    [c] = multcompare(stats,'ctype','bonferroni');

                    % Bonferroni correction for multiple comparisons
                    if strcmp(Correction, 'Bonferroni')
                        [BonfCor_p_values,cor_h]=bonf_holm(On_p_values,0.05);
                        for b = 1:length(cor_h)
                            display(fprintf('Bonferroni correction %s: h= %d,
p=%f\n',gen_str{b+1},cor_h(b),BonfCor_p_values(b))); % b+1 because condition in position 1 is
being compared with all the others
                        end

                        h_all_OnVariable{1} = 'BonfCor Pvalues ON Variable';
                        for s = 2:numberOfgroups
                            h_all_OnVariable{s} = BonfCor_p_values(s-1);
                        end

                    end

                elseif strcmp(Statistics, 'ANOVAn') %Unblanced two-way Anova
                      g1={};
                      g2={};
                      for i = 1:length(groups)
                          tempName =groups{i};
```

```
                g1{i} = tempName(1:5);
                g2{i}= tempName(end);
            end
            [p,tb1,stats] = anovan(Y_all,{g1 g2},'model',2,...
                    'varnames',{'PTX','allele'});
            f2= figure(2); title([EXPERIMENT,variable_str(var),' ON Response ']);
            f4 = figure(4);
            hold on
            [c,m,h,nms] = multcompare(stats,'Dimension',[1 2]);
            title([EXPERIMENT,variable_str(var),' ON Response ']);
        end



    %OFF Variable
        disp('>>>>>>>>>>>> Statistical Analysis for OFF Variable: ');
        h_all_OffVariable = {};
        Off_p_values=[];
        h_all_OffVariable{1} = 'Pvalues OffVariable';
        for s = 2:numberOfgroups
            X = OffVariableMatrix{1};
            Y = OffVariableMatrix{s};
            [h,p1] = ttest2(X,Y); display(fprintf('For X vs Y, h= %d, p=%f',h,p1)); %Ttest
            OnVariablePvalue = sprintf('Pvalue ON: %f ', p1);
            h_all_OffVariable{s} =p1;
            Off_p_values(s-1)= p1;
        end

    end
%% Bar Plot
    y = [On_y;Off_y];
    sem = [On_sem;Off_sem];
    labels= gen_str2;

    if numberOfgroups == 2
       DataSummary = sprintf(['Data Summary: \nGenotype %s:\n ON: %.3f +/- %.3f, \n
OFF %.3f +/- %.3f \n'...
    'Genotype %s:\n ON: %.3f +/- %.3f, \n OFF %.3f +/- %.3f'], labels{1},On_y(1),
On_sem(1), Off_y(1), Off_sem(1),labels{2},On_y(2), On_sem(2), Off_y(2), Off_sem(2));
    elseif numberOfgroups == 3
        DataSummary = sprintf(['Data Summary: \nGenotype %s:\n ON: %.3f +/- %.3f, \n
OFF %.3f +/- %.3f \n'...
    'Genotype %s:\n ON: %.3f +/- %.3f, \n OFF %.3f +/- %.3f\n'...
    'Genotype %s:\n ON: %.3f +/- %.3f, \n OFF %.3f +/- %.3f']...
    , labels{1},On_y(1), On_sem(1), Off_y(1), Off_sem(1),labels{2},On_y(2), On_sem(2),
Off_y(2), Off_sem(2),labels{3},On_y(3), On_sem(3), Off_y(3), Off_sem(3));
    else
        DataSummary = sprintf(['Implement the Code for Data Summary']);
    end

    % Adjusting x position variable for bar plot
    if numberOfgroups == 2
        x= [0.86,1.14];
    elseif numberOfgroups == 3
        x= [0.785,1,1.225];
    elseif numberOfgroups == 4
        x= [0.73,0.915,1.09,1.275];
    elseif numberOfgroups == 7
        x= [0.655,0.77,0.885,1.0,1.115,1.23,1.34];
    elseif numberOfgroups == 8
```

```
        x= [0.65,0.75,0.85,0.95,1.05,1.15,1.25,1.35];
    end

    if numberOfgroups == 2
        %For two groups is useful to have p-values visible in
        %the title
        f5= figure(5); title([EXPERIMENT,variable_str(var),' Response ',
OnVariablePvalue,OffVariablePvalue]);
    else
        f5= figure(5); title([EXPERIMENT,variable_str(var),' Response ']);
    end
    hold on;
    hB = bar(y,'FaceColor','flat');

    hB(1).FaceColor = colorss{1};
    hB(2).FaceColor = colorss{3};

    hAx=gca;  % getting a variable for the current axes handle
    xticks([1 2]);
    hAx.XTickLabel=Variables_str;   % label the ticks

    pause(3) % Pausing here allows matlab to plot text correctly

    hT=[];   % placeholder for text object handles
    for i=1:length(hB)% iterate over number of bar objects
        if numberOfgroups > 3
            hT=[hT,text(hB(i).XData+hB(i).XOffset-
0.05,hB(i).YData,labels(i),'VerticalAlignment','bottom','horizontalalign','center','FontSize',10)];
        else
            hT=[hT,text(hB(i).XData+hB(i).XOffset-
0.05,hB(i).YData,labels(i),'VerticalAlignment','bottom','horizontalalign','center','FontSize',14)];
        end
    end

    ex = x;
    for i=1:size(y,1)  % iterate over Variables
      e = errorbar(ex,y(i,:),sem(i,:),'k.');
      e.LineWidth = 0.75;
      ex = ex +1;
    end



    sz = 10; % Scatter-feature's size
    % Scatter Plot ON
    for j = 1:size(OnVariableMatrix,2)
        xData= x(j);
        for i = 1:size(OnVariableMatrix{j},1)-1
            xData = [xData; x(j)];
        end
        s1 = scatter(xData,OnVariableMatrix{j},sz,'o');
        s1.MarkerEdgeColor = 'k';
%           s1.MarkerFaceColor = 'k';
        hold on
    end
    % Scatter Plot OFF
    for j = 1:size(OffVariableMatrix,2)
        xData= x(j)+1;
        for i = 1:size(OffVariableMatrix{j},1)-1
            xData = [xData; x(j)+1];
```

```
            end
            s1 = scatter(xData,OffVariableMatrix{j},sz,'o');
            s1.MarkerEdgeColor = 'k';
%               s1.MarkerFaceColor = 'k';
            hold on
        end

        % Adding some information to the plot
        if numberOfgroups > 2
            upperlimit = hAx.YLim(2);
            text(1.5,upperlimit/2, h_all_OnVariable,'FontSize',5);
            text(2,upperlimit/2, h_all_OffVariable,'FontSize',5);
            plot_ylim=get(gca,'ylim');
            text(2,plot_ylim(1)-(plot_ylim(1)/3), DataSummary,'FontSize',5);
        else
            plot_ylim=get(gca,'ylim');
            switch NeuronType
                case 'OFF'
                    text(1,plot_ylim(2)-(plot_ylim(2)/3), DataSummary,'FontSize',5);
                case 'ON'
                    text(2,plot_ylim(2)-(plot_ylim(2)/3), DataSummary,'FontSize',5);
            end

        end

        %% Saving Data
        if savingData
           if NORMALIZE_ROI

                cd('C:\MATLAB_FOLDER\2p\2p_pData\5secfff\NORMALIZE_ROI')
                listing =dir(EXPERIMENT);
                if isempty(listing)
                    mkdir(EXPERIMENT);
                end


cd(['C:\MATLAB_FOLDER\2p\2p_pData\5secfff\NORMALIZE_ROI\',EXPERIMENT])
                listing =dir(CONDITION);
                if isempty(listing)
                    mkdir(CONDITION);
                end

cd(['C:\MATLAB_FOLDER\2p\2p_pData\5secfff\NORMALIZE_ROI\',EXPERIMENT,'\',CONDITI
ON])
                saveas(f1,[EXPERIMENT,'_',CONDITION,'_',LAYER,'_5sfff_trace.pdf'])

saveas(f1,[EXPERIMENT,'_',CONDITION,'_',LAYER,'_5sfff_NORMALIZED_trace.pdf'])

saveas(f5,[EXPERIMENT,'_',CONDITION,'_',LAYER,'_5sfff_NORMALIZED_',char(variable_str(
var)),'.pdf'])
           else

                cd(save_in_folder)
                listing =dir(EXPERIMENT);
                if isempty(listing)
                    mkdir(EXPERIMENT);
                end

                cd([save_in_folder,EXPERIMENT])
```

```
                listing =dir(CONDITION);
                if isempty(listing)
                    mkdir(CONDITION);
                end
                cd([save_in_folder,EXPERIMENT,'\',CONDITION])
                saveas(f1,[EXPERIMENT,'_',CONDITION,'_',LAYER,'_5sfff_trace.pdf']);
                if NORMALIZE_FLY

saveas(f5,[EXPERIMENT,'_',CONDITION,'_',LAYER,'_5sfff_NormalyzedByFly_',char(variable_s
tr(var)),'.pdf']);
                    if strcmp(Statistics, 'ANOVA1')

saveas(f2,[EXPERIMENT,'_',CONDITION,'_',LAYER,'_5sfff_NormalyzedByFly_one_way_ANO
VA_table_ON_',char(variable_str(var)),'.pdf']);

saveas(f4,[EXPERIMENT,'_',CONDITION,'_',LAYER,'_5sfff_NormalyzedByFly_one_way_ANO
VA_ON_',char(variable_str(var)),'.fig']);
                        save([EXPERIMENT,'_',CONDITION,'_',LAYER,'_Multiple comparisons
statistics','_ON_',char(variable_str(var)),'.txt'],'-ASCII','c');
                    elseif strcmp(Statistics, 'ANOVA2')

saveas(f2,[EXPERIMENT,'_',CONDITION,'_',LAYER,'_5sfff_NormalyzedByFly_two_way_ANOV
A_table_ON_',char(variable_str(var)),'.pdf']);

saveas(f4,[EXPERIMENT,'_',CONDITION,'_',LAYER,'_5sfff_NormalyzedByFly_two_way_ANOV
A_ON_',char(variable_str(var)),'.fig']);
                    elseif strcmp(Statistics, 'ANOVAn')

saveas(f2,[EXPERIMENT,'_',CONDITION,'_',LAYER,'_5sfff_NormalyzedByFly_two_way_unbal
anced_ANOVA_table_ON_',char(variable_str(var)),'.pdf']);

saveas(f4,[EXPERIMENT,'_',CONDITION,'_',LAYER,'_5sfff_NormalyzedByFly_two_way_unbal
anced_ANOVA_ON_',char(variable_str(var)),'.fig']);
                        save([EXPERIMENT,'_',CONDITION,'_',LAYER,'_Multiple comparisons
statistics','_ON_',char(variable_str(var)),'.txt'],'-ASCII','c');

                    end

                    try

saveas(f3,[EXPERIMENT,'_',CONDITION,'_',LAYER,'_5sfff_trace_NormalyzedByFly.pdf']);
                    catch
                        disp('Normalized trace was not generated');
                    end
                else

saveas(f5,[EXPERIMENT,'_',CONDITION,'_',LAYER,'_5sfff_',char(variable_str(var)),'.pdf']);
                end

              end

            end
            % Initializing structures
            close(f2);
            close all;
            clear x;
            clear allDataCell;
        end
```

end

Fff plot and statistical analysis-related script

Script name: load_experiment_conditions.m

```
% It load the different experiment conditions provided in a txt file
% (Experiments_List.txt)

function
[EXPERIMENT,CONDITION,NEURONCLASS,NORMALIZE_ROI,NORMALIZE_FLY,LAYER,COLORS,pairedData,...

NUMBER_OF_VARIABLES,NUMBER_OF_GROUPS,variable_str,gen_str,DataNames,Statistics,Correction]...

=load_experiment_conditions(Automatized,EXPERIMENT,CONDITION,NEURONCLASS,NORMALIZE_ROI,...

NORMALIZE_FLY,LAYER,COLORS,pairedData,NUMBER_OF_VARIABLES,NUMBER_OF_GROUPS,variable_str,gen_str,DataNames,Statistics,Correction)

if Automatized
    disp('------------ Automatized Analysis MODE ------------')
    pdatapath=('C:\MATLAB_FOLDER\2p\2p_pData');
    cd(pdatapath);

[EXPERIMENT,CONDITION,NEURONCLASS,NORMALIZE_ROI,NORMALIZE_FLY,LAYER,COLORS,pairedData,...

NUMBER_OF_VARIABLES,NUMBER_OF_GROUPS,variable_str,gen_str,DataNames,Statistics,Correction]...
    =textread('Experiments_List_2.txt','%s %s %s %s %s %s %s %s %f %f %q %q %q %s
%s\r','headerlines',1,'delimiter','\t');

    % Transforming all logical strings to logicals
    pairedData = strcmpi(pairedData, 'true');
    NORMALIZE_ROI = strcmpi(NORMALIZE_ROI, 'true');
    NORMALIZE_FLY = strcmpi(NORMALIZE_FLY, 'true');

else
    disp('------------ Not Automatized Analysis MODE ------------');

end
```

Moving edge pre- analysis

Script name: find_centers_ROIs_moving_edge_8dir_elife.m

```
%% Find centers for manually drawn ROIs for 4 or 8 directions moving-edge stimulation. The
input of this script in a folder containing pData files.It finds automatic ROI centers based on
response maximum for ON and OFF epochs, separatelly. It also calculates dF/F for the
responses ans stores it in a variable


% Authors: Miriam Henning and Sebastian Molina-Obando

clc;
clear all;
close all;

%add the path of needed functions
addpath(genpath('C:\MATLAB_FOLDER\TP_code_Seb'));
pData_Folder = uigetdir;
cd(pData_Folder);
N_pDATA=dir('*_pData.mat');

for pDATA=1:size(N_pDATA,1)

        x{1,1}=load(N_pDATA(pDATA).name); % Loading pData file
        Stimulus=x{1,1}.strct.stim_type;  % Checking stimulus



        mb_str =
{'DriftingEdge_LumDecLumInc_1period_20degPerSec_90deg_BlankRand_4Dirs_optimized'};
        mb_str2 = {''};
        mb_str3={''};
        mb_str4={''};

mb_str5={'DriftingEdge_LumDecLumInc_1period_20degPerSec_90deg_BlankRand_8Dirs_opti
mized'};


        GO=0;

        if(strcmp(x{1,1}.strct.stim_type,mb_str))
           GO=1;
        elseif (strcmp(x{1,1}.strct.stim_type,mb_str2))
           GO=1;
        elseif (strcmp(x{1,1}.strct.stim_type,mb_str3))
           GO=2;
        elseif (strcmp(x{1,1}.strct.stim_type,mb_str4))
           GO=2;
        elseif (strcmp(x{1,1}.strct.stim_type,mb_str5))
           GO=3;
        end

        % Only pData with moving edges stimulation will be analysed

        if GO==1||3

           if GO==1
```

```
        NUM_EPOCHS=4;
    elseif GO==3
        NUM_EPOCHS=8;
    end

    % Preparing the matrices analyze the data

    DATA_strct=eval(['x{1,1}.strct']);
    n_cells = size(DATA_strct.masks,2);

    % Finding the  Epochs of stimulus presentation
    fstimval=DATA_strct.fstimval;
    changes=diff(fstimval);
    start_epoch=find(changes>0)+1;
    end_epoch=find(changes<0);
    dur=diff([start_epoch(1:length(end_epoch)),end_epoch]');
    avdur=min(dur);
    ep_all=nan(size(end_epoch,1), avdur);

    %ep_all will contain the range of frames belonging to each stimulus epoch
    for pp=1:length(end_epoch)
        epoch=start_epoch(pp):start_epoch(pp)+avdur-1;
        ep_all(pp,:)=epoch;
    end

    Epochs=ep_all';
    dur=avdur;

    Stim=DATA_strct.fstimval(Epochs(2,:)); % Stimulus identity for each epoch

    %Prepare matrices

    resp_dir=nan(dur,NUM_EPOCHS);
    AV_ROIS_resp=cell(1, n_cells); % This is not yet dF/F it is not even background
subtracted
    centers = zeros(NUM_EPOCHS,n_cells);

    stimuluspos=x{1,1}.strct.fstimpos1;
    stimulus=stimuluspos(Epochs(:,1));

    fps=x{1,1}.strct.xml.framerate;

    Epochs=Epochs(:,1:end-1);  % taking out last epoch, because it can be interrupted
    Stim=Stim(1:end-1);
    Epochs=[Epochs;Epochs(end,:)+1];

    if mod(length(Epochs),2) %If length uneven
        EPLength=length(Epochs)+1;
        Epochs=[Epochs;Epochs(end,:)+1];
    else
        EPLength=length(Epochs);
    end

    ONedgeResp=nan(EPLength/2,NUM_EPOCHS);
    OFFedgeResp=nan(EPLength/2,NUM_EPOCHS);
    resp_dir=nan(EPLength,NUM_EPOCHS);

    % Taking the OFF-edge and ON-edge indices to cut the responses of each ROI/cell
```

```
for k=1:n_cells  %for each cell
    ROI_Resp=DATA_strct.avSignal1(k,:); % Not background subtracted

    Mask=DATA_strct.masks{1,k}; % Mask of this cell
    centers(k) = 500;   % preset to default values;

    try
        BaseLine=mean(DATA_strct.BaseLine,3); %If a baseline sequence is there, take it
        Baselineresp=BaseLine(Mask);
        avBaselineresp=mean(Baselineresp); % Average Baseline response
    catch
        BaseLine=mean(DATA_strct.avSignal1(k,:)); % If no baseline recorded,the mean of
the whole trace is taken as baseline.
        avBaselineresp=BaseLine; % Average Baseline response
    end

    %dF/F Calculation.(F(stim)-F(rest))/F(rest)
    ROI_Resp_DF=(ROI_Resp-avBaselineresp)/avBaselineresp;

    %Trial averaging
    for kk=1:NUM_EPOCHS % loop through all directions of movement
        Double=find(Stim==kk);
        if length(Double)>1
            allepochs=[];
            for pp=1:length(Double)
                allepochs=[allepochs; ROI_Resp_DF(Epochs(:,Double(pp)))];
            end
            resp_dir(:,kk)=mean(allepochs,1); % Mean ROI response across repetitions of
stimulus direction
        else
            resp_dir(:,kk)= ROI_Resp_DF(Epochs(:,Double));

        end
        OFFedgeResp(:,kk)=resp_dir(1:round(length(resp_dir)/2),kk);
        ONedgeResp(:,kk)=resp_dir(round(length(resp_dir)/2)+1:length(resp_dir),kk);
    end



    %Response maximum
    Centers_i=zeros(NUM_EPOCHS,1);
    for kk=1:NUM_EPOCHS %for each stimulus direction
        % calculate the maximum of the response, which is later
        % used to shift the response into the center.

        [maxvON,TmaxON]=max(ONedgeResp(:,kk));
        [maxvOFF,TmaxOFF]=max(OFFedgeResp(:,kk));

        %Is the response to the OFF edge higher or to ON edge?
        OFFneuron=maxvOFF>maxvON;

        if OFFneuron ==1 % If response to OFF edge is higher

            if maxvOFF>mean(OFFedgeResp(:,kk))+1*std(OFFedgeResp(:,kk)) % If
response pass a threshold...
                Centers_i(kk)=TmaxOFF;    %... the max response is shifted to the center.
            end
```

```
                elseif OFFneuron==0 % If response of ON edge is higher

                    if maxvON>mean(ONedgeResp(:,kk))+1*std(ONedgeResp(:,kk)) % If response
pass a threshold...
                        Centers_i(kk)=TmaxON;     %... the max response is shifted to the center.
                    end
                end

            end

            %Contains the  centers (directions) of each ROIs
            centers(:,k)=Centers_i;

            Middle=round(size(ONedgeResp,1)/2);
            shifts=zeros(NUM_EPOCHS,1);
            for ll=1:NUM_EPOCHS;
                if Centers_i(ll)>0;
                    shifts(ll) = Middle- Centers_i(ll);
                end
            end


            resp_dir_cut=zeros(size(resp_dir));

            for lll=1:NUM_EPOCHS
                resp_dir_cut(:,lll)=circshift(resp_dir(:,lll),shifts(lll));
            end

            AV_ROIS_resp{1,k}=resp_dir_cut';
        end

    end

    %Saving centers data in each pData file
    if GO==1||2||3

        x{1,1}.strct.centers=centers;
        x{1,1}.strct.AV_ROIS_resp=AV_ROIS_resp;

        out = x{1,1}.strct;
        strct=out;
        if(isfield(strct,'ch1'))
            strct = rmfield(strct,'ch1');
            strct = rmfield(strct,'ch1b');
            strct = rmfield(strct,'ch3');
        end

        pathroot=pData_Folder;
        cd(pathroot)
        save(N_pDATA(pDATA).name,'strct' );
    end
end
```

Moving edge analysis

Script name: moving_edge_analysis_elife.m

Sub-functions needed:

create_experimental_conditions_structure_elife.m

load_neuron_data10Hz_byLayer_avResp_elife.m

aggregate_avROIResp_elife.m

plot_err_patch_v2.m

```
% This script analyses moving ON and OFF edges from manually selected ROIs. It generates
some plots to visualized the data and saves a matlab file with all important variable for future
use.

% Authors: Miriam Henning and Sebastian Molina-Obando


% clc;
clear all;
close all;


%% USER INPUT: CONSTANTS

savingData = true; %"True" for saving data (variables and plots) in a folder in your computer.
Otherwise, write "false".

%Naming folders to store data
save_as= 'D Mi 2.5uM PTX Control'; %'+ +', 'D +', 'Df +', 'D Df', '+ + 2.5uM PTX', '+ + 2.5uM PTX
Control', 'D Mi',
save_in_folder =
'C:\MATLAB_FOLDER\2p\2p_pData\T4_T5_Layer_Data\T4_T5_LexA_LexAop_GCaMP6f_rec2
8\LumDecLumInc_8Dir_opt';

% Choose (comment out) the experimental conditions. Choos only one.

    %
Condition='i_drifting_edge_20degps_Rand8DIR_IncDec_opt.*i_T4_T5_LexA_LexAop_GCaMP
6f_28rec.*i_Control';
    %
Condition='i_drifting_edge_20degps_Rand8DIR_IncDec_opt.*i_T4_T5_LexA_LexAop_GCaMP
6f_28rec_GluClalphaFlpSTOP.*i_Control.*i_GluCla_KD_exp';
    %
Condition='i_drifting_edge_20degps_Rand8DIR_IncDec_opt.*i_T4_T5_LexA_LexAop_GCaMP
6f_28rec_DfED6025.*i_PTX_Control';
    %
Condition='i_drifting_edge_20degps_Rand8DIR_IncDec_opt.*i_T4_T5_LexA_LexAop_GCaMP
6f_28rec_GluClalphaFlpSTOP_DfED6025.*i_Control.*i_GluCla_KD_exp';

    %
Condition='i_drifting_edge_20degps_Rand8DIR_IncDec_opt.*i_T4_T5_LexA_LexAop_GCaMP
6f_28rec_GluCla_MiMIC_14426_GluCla_Flp_D.*i_GluCla_KO_exp_PTX_2_5uM';
```

```
    %
Condition='i_drifting_edge_20degps_Rand8DIR_IncDec_opt.*i_T4_T5_LexA_LexAop_GCaMP
6f_28rec_GluCla_MiMIC_14426_GluCla_Flp_D.*i_PTX_2_5uM.*i_Exp1.*i_PTX_exp_2_5uM';
    %
Condition='i_drifting_edge_20degps_Rand8DIR_IncDec_opt.*i_T4_T5_LexA_LexAop_GCaMP
6f_28rec_GluCla_MiMIC_14426_GluCla_Flp_D.*i_PTX_2_5uM_3min.*i_Exp1.*i_PTX_exp_2_5
uM';

Condition='i_drifting_edge_20degps_Rand8DIR_IncDec_opt.*i_T4_T5_LexA_LexAop_GCaMP
6f_28rec_GluCla_MiMIC_14426_GluCla_Flp_D.*i_Exp2.*i_GluCla_KO_exp_PTX_2_5uM';

    %
Condition='i_drifting_edge_20degps_Rand8DIR_IncDec_opt.*i_T4_T5_LexA_LexAop_GCaMP
6f_28rec_GluCla_MiMIC_14426.*i_Exp1.*i_GluCla_KO_exp';


%Condition='i_drifting_edge_20degps_Rand8DIR_IncDec_opt.*i_T4_T5_LexA_LexAop_GCaM
P6f_28rec.*i_PTX_Control.*i_PTX_exp_2_5uM';
    %
Condition='i_drifting_edge_20degps_Rand8DIR_IncDec_opt.*i_T4_T5_LexA_LexAop_GCaMP
6f_28rec.*i_PTX_2_5uM.*i_PTX_exp_2_5uM';

    %
Condition='i_drifting_edge_20degps_Rand8DIR_IncDec_opt.*i_T4_T5_LexA_LexAop_GCaMP
6f_28rec.*i_PTX_Control.*i_Exp1.*i_PTX_exp_5uM';
    %
Condition='i_drifting_edge_20degps_Rand8DIR_IncDec_opt.*i_T4_T5_LexA_LexAop_GCaMP
6f_28rec.*i_PTX_5uM.*i_Exp1.*i_PTX_exp_5uM';

    %
Condition='i_drifting_edge_20degps_Rand8DIR_IncDec_opt.*i_T4_T5_LexA_LexAop_GCaMP
6f_28rec.*i_PTX_Control.*i_Exp1.*i_PTX_exp_100uM';
    %
Condition='i_drifting_edge_20degps_Rand8DIR_IncDec_opt.*i_T4_T5_LexA_LexAop_GCaMP
6f_28rec.*i_PTX_100uM.*i_Exp1.*i_PTX_exp_100uM';



    %
Condition='i_drifting_edge_20degps_Rand8DIR_IncDec_opt.*i_T4_T5_LexA_LexAop_GCaMP
6f_28rec.*i_PTX_Control_1min.*i_Exp1.*i_PTX_exp_100uM';
    %
Condition='i_drifting_edge_20degps_Rand8DIR_IncDec_opt.*i_T4_T5_LexA_LexAop_GCaMP
6f_28rec.*i_PTX_100uM_1min.*i_Exp1.*i_PTX_exp_100uM';

    %
Condition='i_drifting_edge_20degps_Rand8DIR_IncDec_opt.*i_T4_T5_LexA_LexAop_GCaMP
6f_28rec.*i_MPEP_Control.*i_Exp1.*i_MPEP_exp_100uM';
    %
Condition='i_drifting_edge_20degps_Rand8DIR_IncDec_opt.*i_T4_T5_LexA_LexAop_GCaMP
6f_28rec.*i_MPEP_100uM.*i_Exp1.*i_MPEP_exp_100uM';

%% Analysis


    %add the path of needed functions
    addpath(genpath('C:\MATLAB_FOLDER\TP_code_Seb'));
    pdatapath='C:\MATLAB_FOLDER\2p\2p_pData';
    addpath(pdatapath);
    cd(pdatapath);
```

```
    database_select_samples_seb; % Loading information from database
    experimental_conditions = find(eval(Condition)); %% Concatenation of all conditions that
define the experimental GENOTYPE

    % Creating a data structure with info about pdata to load:
    ExpCondStrct = create_experimental_conditions_structure_elife(experimental_conditions);

    % Loading all the pDatas and save info in a structure for each LAYER.
    %Here we interpolates our data at 10 Hz

    T4T5_LPA = load_neuron_data10Hz_byLayer_avResp_elife(ExpCondStrct,pdatapath,'LPA');
    T4T5_LPB = load_neuron_data10Hz_byLayer_avResp_elife(ExpCondStrct,pdatapath,'LPB');
    T4T5_LPC = load_neuron_data10Hz_byLayer_avResp_elife(ExpCondStrct,pdatapath,'LPC');
    T4T5_LPD = load_neuron_data10Hz_byLayer_avResp_elife(ExpCondStrct,pdatapath,'LPD');
    T4T5_M9 = load_neuron_data10Hz_byLayer_avResp_elife(ExpCondStrct,pdatapath,'M9');
    try
        T4T5_Lo = load_neuron_data10Hz_byLayer_avResp_elife(ExpCondStrct,pdatapath,'Lo');
        T4T5_Lo_agg=aggregate_avROIResp_elife(T4T5_Lo);
        Lo_LAYER = 1;
    catch
        T4T5_Lo = 'NO Lo LAYER in this data set';
        T4T5_Lo_agg = 'NO Lo LAYER in this data set';
        disp('>>>>>>> This Data set has NO Lo LAYER');
        Lo_LAYER = 0;
    end



   % Aggregation (trial average) of all selected ROI responses

    T4T5_LPA_agg=aggregate_avROIResp_elife(T4T5_LPA);
    T4T5_LPB_agg=aggregate_avROIResp_elife(T4T5_LPB);
    T4T5_LPC_agg=aggregate_avROIResp_elife(T4T5_LPC);
    T4T5_LPD_agg=aggregate_avROIResp_elife(T4T5_LPD);
    T4T5_M9_agg=aggregate_avROIResp_elife(T4T5_M9);




 %%  Plotting

    %*************************************************
    %  PLOTS - by FLY
    %*************************************************

            COLOR_OF_PLOT_A = [0 .5 0]; % GREEN
            COLOR_CLOUD_A =[.5 .7 .5];  % GREEN cloud

            COLOR_OF_PLOT_B = [0 0 1];  %BLUE;
            COLOR_CLOUD_B = [.5 .5 1];  %BLUE cloud

            COLOR_OF_PLOT_C = [1 0 0];  %RED
            COLOR_CLOUD_C = [1 .5 .5];  %RED cloud

            COLOR_OF_PLOT_D = [.7 .7 0];%YELLOW
            COLOR_CLOUD_D = [.9 .9 .5]; %YELLOW cloud
```

```
COLOR_OF_PLOT_M9 = [99/256 99/256 99/256]; %GREY
COLOR_CLOUD_M9 = [189/256 189/256 189/256]; %GREY cloud

TRACE_OFFSET_PLOT = 6; % spacing in dF/F between the traces for the 4 directions

t=[0.1:0.1:size(T4T5_LPA_agg.rats,3)/10];


F1=  figure(1);

subplot(2,2,1);hold on; %Layer 1/A

patch([0 4 4 0]',[-0.5 -0.5 45 45]',[0.85 0.85 0.85],'EdgeColor','none');
set(gca, 'XLim', [0 8])
set(gca, 'YLim', [-0.5 45])


plot(t,squeeze(T4T5_LPA_agg.MeanOF_Cells(:,1,:))+0*TRACE_OFFSET_PLOT,'Color',COLO
R_OF_PLOT_A);

plot(t,squeeze(T4T5_LPA_agg.MeanOF_Cells(:,2,:))+1*TRACE_OFFSET_PLOT,'Color',COLO
R_OF_PLOT_A);

plot(t,squeeze(T4T5_LPA_agg.MeanOF_Cells(:,3,:))+2*TRACE_OFFSET_PLOT,'Color',COLO
R_OF_PLOT_A);

plot(t,squeeze(T4T5_LPA_agg.MeanOF_Cells(:,4,:))+3*TRACE_OFFSET_PLOT,'Color',COLO
R_OF_PLOT_A);

plot(t,squeeze(T4T5_LPA_agg.MeanOF_Cells(:,5,:))+4*TRACE_OFFSET_PLOT,'Color',COLO
R_OF_PLOT_A);

plot(t,squeeze(T4T5_LPA_agg.MeanOF_Cells(:,6,:))+5*TRACE_OFFSET_PLOT,'Color',COLO
R_OF_PLOT_A);

plot(t,squeeze(T4T5_LPA_agg.MeanOF_Cells(:,7,:))+6*TRACE_OFFSET_PLOT,'Color',COLO
R_OF_PLOT_A);

plot(t,squeeze(T4T5_LPA_agg.MeanOF_Cells(:,8,:))+7*TRACE_OFFSET_PLOT,'Color',COLO
R_OF_PLOT_A);

title('Layer1/A')


subplot(2,2,2);hold on; %Layer 2/B

patch([0 4 4 0]',[-0.5 -0.5 45 45]',[0.85 0.85 0.85],'EdgeColor','none');
set(gca, 'XLim', [0 8])
set(gca, 'YLim', [-0.5 45])


plot(t,squeeze(T4T5_LPB_agg.MeanOF_Cells(:,1,:))+0*TRACE_OFFSET_PLOT,'Color',COLO
R_OF_PLOT_B);

plot(t,squeeze(T4T5_LPB_agg.MeanOF_Cells(:,2,:))+1*TRACE_OFFSET_PLOT,'Color',COLO
R_OF_PLOT_B);
```

```
plot(t,squeeze(T4T5_LPB_agg.MeanOF_Cells(:,3,:))+2*TRACE_OFFSET_PLOT,'Color',COLO
R_OF_PLOT_B);

plot(t,squeeze(T4T5_LPB_agg.MeanOF_Cells(:,4,:))+3*TRACE_OFFSET_PLOT,'Color',COLO
R_OF_PLOT_B);

plot(t,squeeze(T4T5_LPB_agg.MeanOF_Cells(:,5,:))+4*TRACE_OFFSET_PLOT,'Color',COLO
R_OF_PLOT_B);

plot(t,squeeze(T4T5_LPB_agg.MeanOF_Cells(:,6,:))+5*TRACE_OFFSET_PLOT,'Color',COLO
R_OF_PLOT_B);

plot(t,squeeze(T4T5_LPB_agg.MeanOF_Cells(:,7,:))+6*TRACE_OFFSET_PLOT,'Color',COLO
R_OF_PLOT_B);

plot(t,squeeze(T4T5_LPB_agg.MeanOF_Cells(:,8,:))+7*TRACE_OFFSET_PLOT,'Color',COLO
R_OF_PLOT_B);

 title('Layer2/B')

 subplot(2,2,3);hold on; %Layer 3/C

 patch([0 4 4 0]',[-0.5 -0.5 45 45]',[0.85 0.85 0.85],'EdgeColor','none');
 set(gca, 'XLim', [0 8])
 set(gca, 'YLim', [-0.5 45])


plot(t,squeeze(T4T5_LPC_agg.MeanOF_Cells(:,1,:))+0*TRACE_OFFSET_PLOT,'Color',COLO
R_OF_PLOT_C);

plot(t,squeeze(T4T5_LPC_agg.MeanOF_Cells(:,2,:))+1*TRACE_OFFSET_PLOT,'Color',COLO
R_OF_PLOT_C);

plot(t,squeeze(T4T5_LPC_agg.MeanOF_Cells(:,3,:))+2*TRACE_OFFSET_PLOT,'Color',COLO
R_OF_PLOT_C);

plot(t,squeeze(T4T5_LPC_agg.MeanOF_Cells(:,4,:))+3*TRACE_OFFSET_PLOT,'Color',COLO
R_OF_PLOT_C);

plot(t,squeeze(T4T5_LPC_agg.MeanOF_Cells(:,5,:))+4*TRACE_OFFSET_PLOT,'Color',COLO
R_OF_PLOT_C);

plot(t,squeeze(T4T5_LPC_agg.MeanOF_Cells(:,6,:))+5*TRACE_OFFSET_PLOT,'Color',COLO
R_OF_PLOT_C);

plot(t,squeeze(T4T5_LPC_agg.MeanOF_Cells(:,7,:))+6*TRACE_OFFSET_PLOT,'Color',COLO
R_OF_PLOT_C);

plot(t,squeeze(T4T5_LPC_agg.MeanOF_Cells(:,8,:))+7*TRACE_OFFSET_PLOT,'Color',COLO
R_OF_PLOT_C);

 title('Layer3/C')

 subplot(2,2,4);hold on; %Layer 4/D

 patch([0 4 4 0]',[-0.5 -0.5 45 45]',[0.85 0.85 0.85],'EdgeColor','none');
 set(gca, 'XLim', [0 8])
 set(gca, 'YLim', [-0.5 45])
```

```
plot(t,squeeze(T4T5_LPD_agg.MeanOF_Cells(:,1,:))+0*TRACE_OFFSET_PLOT,'Color',COLO
R_OF_PLOT_D);

plot(t,squeeze(T4T5_LPD_agg.MeanOF_Cells(:,2,:))+1*TRACE_OFFSET_PLOT,'Color',COLO
R_OF_PLOT_D);

plot(t,squeeze(T4T5_LPD_agg.MeanOF_Cells(:,3,:))+2*TRACE_OFFSET_PLOT,'Color',COLO
R_OF_PLOT_D);

plot(t,squeeze(T4T5_LPD_agg.MeanOF_Cells(:,4,:))+3*TRACE_OFFSET_PLOT,'Color',COLO
R_OF_PLOT_D);

plot(t,squeeze(T4T5_LPD_agg.MeanOF_Cells(:,5,:))+4*TRACE_OFFSET_PLOT,'Color',COLO
R_OF_PLOT_D);

plot(t,squeeze(T4T5_LPD_agg.MeanOF_Cells(:,6,:))+5*TRACE_OFFSET_PLOT,'Color',COLO
R_OF_PLOT_D);

plot(t,squeeze(T4T5_LPD_agg.MeanOF_Cells(:,7,:))+6*TRACE_OFFSET_PLOT,'Color',COLO
R_OF_PLOT_D);

plot(t,squeeze(T4T5_LPD_agg.MeanOF_Cells(:,8,:))+7*TRACE_OFFSET_PLOT,'Color',COLO
R_OF_PLOT_D);

 title('Layer4/D')



  %%
    %**************************************************
    %  PLOTS - average across FLY
    %**************************************************



    F2= figure(2);
     subplot(2,2,1)
     patch([0 4 4 0]',[-0.5 -0.5 45 45]',[0.85 0.85 0.85],'EdgeColor','none');
     set(gca, 'XLim', [0 8])
     set(gca, 'YLim', [-0.5 45])

    for i=1:8;
       plot_err_patch_v2(t, TRACE_OFFSET_PLOT*(i-
1)+squeeze(nanmean(T4T5_LPA_agg.MeanOF_Cells(:,i,:),1))',...

squeeze(nanstd(T4T5_LPA_agg.MeanOF_Cells(:,i,:),1)),COLOR_OF_PLOT_A,COLOR_CLOU
D_A);
       line([t(1) t(end)],[TRACE_OFFSET_PLOT*(i-1)  TRACE_OFFSET_PLOT*(i-1)],...
       'color',[0 0 0],'LineStyle','--');
    end

   title(['T4&T5  LayerA - NFlies = ', num2str(size(T4T5_LPA_agg.MeanOF_Cells,1))])



       subplot(2,2,2)
     patch([0 4 4 0]',[-0.5 -0.5 45 45]',[0.85 0.85 0.85],'EdgeColor','none');
     set(gca, 'XLim', [0 8])
     set(gca, 'YLim', [-0.5 45])
```

```
    for i=1:8;
       plot_err_patch_v2(t, TRACE_OFFSET_PLOT*(i-
1)+squeeze(nanmean(T4T5_LPB_agg.MeanOF_Cells(:,i,:),1))',...

squeeze(nanstd(T4T5_LPB_agg.MeanOF_Cells(:,i,:),1)),COLOR_OF_PLOT_B,COLOR_CLOU
D_B);
       line([t(1) t(end)],[TRACE_OFFSET_PLOT*(i-1)  TRACE_OFFSET_PLOT*(i-1)],...
       'color',[0 0 0],'LineStyle','--');
    end

   title(['T4&T5  LayerB - NFlies = ', num2str(size(T4T5_LPB_agg.MeanOF_Cells,1))])



        subplot(2,2,3)
     patch([0 4 4 0]',[-0.5 -0.5 45 45]',[0.85 0.85 0.85],'EdgeColor','none');
     set(gca, 'XLim', [0 8])
     set(gca, 'YLim', [-0.5 45])

    for i=1:8;
       plot_err_patch_v2(t, TRACE_OFFSET_PLOT*(i-
1)+squeeze(nanmean(T4T5_LPC_agg.MeanOF_Cells(:,i,:),1))',...

squeeze(nanstd(T4T5_LPC_agg.MeanOF_Cells(:,i,:),1)),COLOR_OF_PLOT_C,COLOR_CLOU
D_C);
       line([t(1) t(end)],[TRACE_OFFSET_PLOT*(i-1)  TRACE_OFFSET_PLOT*(i-1)],...
       'color',[0 0 0],'LineStyle','--');
    end

   title(['T4&T5  LayerC - NFlies = ', num2str(size(T4T5_LPC_agg.MeanOF_Cells,1))])



        subplot(2,2,4)
     patch([0 4 4 0]',[-0.5 -0.5 45 45]',[0.85 0.85 0.85],'EdgeColor','none');
     set(gca, 'XLim', [0 8])
     set(gca, 'YLim', [-0.5 45])

    for i=1:8;
       plot_err_patch_v2(t, TRACE_OFFSET_PLOT*(i-
1)+squeeze(nanmean(T4T5_LPD_agg.MeanOF_Cells(:,i,:),1))',...

squeeze(nanstd(T4T5_LPD_agg.MeanOF_Cells(:,i,:),1)),COLOR_OF_PLOT_D,COLOR_CLOU
D_D);
       line([t(1) t(end)],[TRACE_OFFSET_PLOT*(i-1)  TRACE_OFFSET_PLOT*(i-1)],...
       'color',[0 0 0],'LineStyle','--');
    end

   title(['T4&T5  LayerD - NFlies = ', num2str(size(T4T5_LPD_agg.MeanOF_Cells,1))])


 %%

    %*********************************************
    %  PLOTS - by FLY Medulla Layer
    %*********************************************

 F3=  figure(3);
```

```
hold on; %Layer M9

 patch([0 4 4 0]',[-0.5 -0.5 45 45]',[0.85 0.85 0.85],'EdgeColor','none');
 set(gca, 'XLim', [0 8])
 set(gca, 'YLim', [-0.5 45])


plot(t,squeeze(T4T5_M9_agg.MeanOF_Cells(:,1,:))+0*TRACE_OFFSET_PLOT,'Color',COLOR
_OF_PLOT_M9);

plot(t,squeeze(T4T5_M9_agg.MeanOF_Cells(:,2,:))+1*TRACE_OFFSET_PLOT,'Color',COLOR
_OF_PLOT_M9);

plot(t,squeeze(T4T5_M9_agg.MeanOF_Cells(:,3,:))+2*TRACE_OFFSET_PLOT,'Color',COLOR
_OF_PLOT_M9);

plot(t,squeeze(T4T5_M9_agg.MeanOF_Cells(:,4,:))+3*TRACE_OFFSET_PLOT,'Color',COLOR
_OF_PLOT_M9);

plot(t,squeeze(T4T5_M9_agg.MeanOF_Cells(:,5,:))+4*TRACE_OFFSET_PLOT,'Color',COLOR
_OF_PLOT_M9);

plot(t,squeeze(T4T5_M9_agg.MeanOF_Cells(:,6,:))+5*TRACE_OFFSET_PLOT,'Color',COLOR
_OF_PLOT_M9);

plot(t,squeeze(T4T5_M9_agg.MeanOF_Cells(:,7,:))+6*TRACE_OFFSET_PLOT,'Color',COLOR
_OF_PLOT_M9);

plot(t,squeeze(T4T5_M9_agg.MeanOF_Cells(:,8,:))+7*TRACE_OFFSET_PLOT,'Color',COLOR
_OF_PLOT_M9);

 title('Layer M9')



 F4= figure(4);
     patch([0 4 4 0]',[-0.5 -0.5 45 45]',[0.85 0.85 0.85],'EdgeColor','none');
     set(gca, 'XLim', [0 8])
     set(gca, 'YLim', [-0.5 45])

    for i=1:8;
       plot_err_patch_v2(t, TRACE_OFFSET_PLOT*(i-
1)+squeeze(nanmean(T4T5_M9_agg.MeanOF_Cells(:,i,:),1))',...

squeeze(nanstd(T4T5_M9_agg.MeanOF_Cells(:,i,:),1)),COLOR_OF_PLOT_M9,COLOR_CLOU
D_M9);
       line([t(1) t(end)],[TRACE_OFFSET_PLOT*(i-1)  TRACE_OFFSET_PLOT*(i-1)],...
       'color',[0 0 0],'LineStyle','--');
    end

   title(['T4&T5  Layer M9 - NFlies = ', num2str(size(T4T5_LPA_agg.MeanOF_Cells,1))])


 %%
 %%

    %***********************************************
    %  PLOTS - by FLY Lo Layer
```

```
    %**********************************************

    if  Lo_LAYER
        F5=  figure(5);

        hold on; %Layer Lo

         patch([0 4 4 0]',[-0.5 -0.5 45 45]',[0.85 0.85 0.85],'EdgeColor','none');
         set(gca, 'XLim', [0 8])
         set(gca, 'YLim', [-0.5 45])


plot(t,squeeze(T4T5_Lo_agg.MeanOF_Cells(:,1,:))+0*TRACE_OFFSET_PLOT,'Color',COLOR_
OF_PLOT_M9);

plot(t,squeeze(T4T5_Lo_agg.MeanOF_Cells(:,2,:))+1*TRACE_OFFSET_PLOT,'Color',COLOR_
OF_PLOT_M9);

plot(t,squeeze(T4T5_Lo_agg.MeanOF_Cells(:,3,:))+2*TRACE_OFFSET_PLOT,'Color',COLOR_
OF_PLOT_M9);

plot(t,squeeze(T4T5_Lo_agg.MeanOF_Cells(:,4,:))+3*TRACE_OFFSET_PLOT,'Color',COLOR_
OF_PLOT_M9);

plot(t,squeeze(T4T5_Lo_agg.MeanOF_Cells(:,5,:))+4*TRACE_OFFSET_PLOT,'Color',COLOR_
OF_PLOT_M9);

plot(t,squeeze(T4T5_Lo_agg.MeanOF_Cells(:,6,:))+5*TRACE_OFFSET_PLOT,'Color',COLOR_
OF_PLOT_M9);

plot(t,squeeze(T4T5_Lo_agg.MeanOF_Cells(:,7,:))+6*TRACE_OFFSET_PLOT,'Color',COLOR_
OF_PLOT_M9);

plot(t,squeeze(T4T5_Lo_agg.MeanOF_Cells(:,8,:))+7*TRACE_OFFSET_PLOT,'Color',COLOR_
OF_PLOT_M9);

         title('Layer Lo')



         F6= figure(6);
             patch([0 4 4 0]',[-0.5 -0.5 45 45]',[0.85 0.85 0.85],'EdgeColor','none');
             set(gca, 'XLim', [0 8])
             set(gca, 'YLim', [-0.5 45])

            for i=1:8;
               plot_err_patch_v2(t, TRACE_OFFSET_PLOT*(i-
1)+squeeze(nanmean(T4T5_Lo_agg.MeanOF_Cells(:,i,:),1))',...

squeeze(nanstd(T4T5_Lo_agg.MeanOF_Cells(:,i,:),1)),COLOR_OF_PLOT_M9,COLOR_CLOU
D_M9);
               line([t(1) t(end)],[TRACE_OFFSET_PLOT*(i-1)  TRACE_OFFSET_PLOT*(i-1)],...
               'color',[0 0 0],'LineStyle','--');
            end

           title(['T4&T5  Layer Lo - NFlies = ', num2str(size(T4T5_LPA_agg.MeanOF_Cells,1))])

    end
```

```
%% Saving Data

if savingData

   set(F1,'PaperSize', [10 10])
   set(F2,'PaperSize', [10 10])
   set(F3,'PaperSize', [10 10])
   set(F4,'PaperSize', [10 10])

   saveas(F2, [save_in_folder,'\', save_as,'\Average T4&T5 responses to Edges manually drawn ROIs 8Dir_Ax.pdf'])
   saveas(F4, [save_in_folder,'\', save_as,'\Average T4&T5 responses to Edges manually drawn ROIs 8Dir_M9.pdf'])
   try
       set(F5,'PaperSize', [10 10])
       set(F6,'PaperSize', [10 10])
       saveas(F6, [save_in_folder,'\', save_as,'\Average T4&T5 responses to Edges manually drawn ROIs 8Dir_Lo.pdf'])
   catch
        disp('>>>>>>> NO Plots for Lo LAYER');
   end

   save([save_in_folder,'\',
save_as,'\processed_Data_manualROIs'],'T4T5_LPA','T4T5_LPB','T4T5_LPC','T4T5_LPD','T4T5_M9','T4T5_Lo','T4T5_LPA_agg','T4T5_LPB_agg','T4T5_LPC_agg','T4T5_LPD_agg','T4T5_M9_agg','T4T5_Lo_agg');
end
```

Moving edge analysis-related script

Script name: load_neuron_data10Hz_byLayer_avResp_elife.m

```
% This function figures out,among all recorded ROIs, which ROI belongs
% to a specific layer. The identification number of each ROIs in each layer
% is specified in the "layer" column of the masterfolder txt file. For all
% selected ROIs, important information is extracted and created. E.g. the stimulus and response time vectors are genertaed and
% extrapolated to 10 Hz.



function out=load_neuron_data10Hz_byLayer_avResp_elife(in,locdir, layerTypeWanted)

% Reads the masterfolder and seperates the different categories
[fname,datetime,frames,zdepth,tottime,stimbouts,locnum,...
   activecells,inverted,stimcode,quality,driver,...
   moving,layer,wavelength,flyID, responsivefly,condition1, condition2, condition3]...
   =textread('MASTER_foldersummary_Seb.txt',...
   '%s %s %f %f %f %f %f %s %f %s %f %s %f %s %f %f %f %s %s %s \r',...
   'headerlines',1,'delimiter','\t');
%% Reads now string for layers
currd=pwd;
cd(locdir);

count=1;
```

```
for ii=1:length(in)  % all the presentations of the correct stimulus by ROI

    qq=in(ii).cell;  % which ROI 'cell' we are currently on...
    ss=strcmpi(in(ii).name,fname);  % logical that tells you which LDM out of all of them we are
currently on.
    if exist(in(ii).name) * (qq>0)
        x=load(in(ii).name);  % open that data file

        currLayerInfo=layer{ss};
        % now use this info to determine if the current "cell" value stored
        % in qq matches any of the numbers that follow the "layer" that
        % we want  e.g. LPA 1:5
        str_i_layer=strfind(currLayerInfo, layerTypeWanted); % gives you the first location of
layerTypeWanted in currLayerInfo

        if(~isempty(str_i_layer))
            str_start= str_i_layer + length(layerTypeWanted)-1;
            i=str_start; % index one before where the numbers will start

            while(true)
                i=i+1;

                if(isletter(currLayerInfo(i))) % a letter denotes next layer "label tag"
                    break;
                end

                if(i==length(currLayerInfo)) % end of the layer string
                    i=i+1; % this is the last number so step one more to get the indexing to include it....
                    break;
                end

            end
            %Numbers of the ROI's that we want the data from
            layersToUse=str2num(currLayerInfo(str_start+1:i-1));


            if qq<=size(x.strct.dSignal1,1) && sum(layersToUse==qq) % AND this ROI is within the
correct
                % if this ROI is within the 'layerToUse' list of number of ROIs recorded
                % and if this ROI was listed within the wanted layer of the
                % brain...
            try
                out(count).layer=layer(ss);
                out(count).flyID=flyID(ss);
                out(count).name=in(ii).name;
                out(count).cell=in(ii).cell;



                frame_nums = x.strct.frame_nums;
                fps=x.strct.xml.framerate;
                %now: # frames / framerate makes timing vector
                AV_ROIS_resp=x.strct.AV_ROIS_resp{1,qq};
                t=[1:length(AV_ROIS_resp)]/fps;
                %Timing vector for interpolation at 10Hz (0.1), starts at ends with 0.5/fps shift
                it=[0.5/fps:0.1:(length(AV_ROIS_resp)+0.5)/fps];



                iAV_ROI_resp=nan(size(AV_ROIS_resp,1), length(it));
```

```
            for oo=1:size(AV_ROIS_resp,1)
                iAV_ROI_resp(oo,:)=interp1(t,AV_ROIS_resp(oo,:),it,'linear','extrap');
            end

            out(count).iAV_ROI_resp=iAV_ROI_resp;

            count=count+1;
        catch
        end

        end
    end

    else
        disp([in(ii).name ' unfound -- skipping']);
    end
end

cd(currd);
```

Moving edge analysis-related script

Script name: aggregate_avROIResp_elife.m

```
%It aggregates (trial average)responses for all ROIs and organiye data by fly.

function out = aggregate_avROIResp_elife(in)

n_epoch=size(in(1).iAV_ROI_resp,1);
%find min epoch duration, therefore take randomly responses of cells from
%diff flies and check the length of the AV_ROI_resp:

Ind=randi(length(in),10);
for IND=1:size(Ind,1);
    dur(IND)=size(in(Ind(IND)).iAV_ROI_resp,2);
end
dur= min(dur);


rats = zeros([length(in),n_epoch,dur]); % all cell responses
name = cell(length(in),1);
flyID=zeros(length(in),1);
for ii=1:length(in)

    rats(ii,:,:) = in(ii).iAV_ROI_resp(:,1:dur);
    name{ii}=in(ii).name;


end

flyID = [in.flyID];
Flys=unique(flyID);

MeanOf_Cells=zeros(length(Flys),n_epoch,dur);
```

```
StdOf_Cells=zeros(length(Flys),n_epoch,dur);

for NFly=1:length(Flys)

    FlyID=Flys(NFly);
    Ind=find(flyID==FlyID);

    MeanOf_Cells(NFly,:,:)=mean(rats(Ind,:,:),1);
    StdOf_Cells(NFly,:,:)=std(rats(Ind,:,:),1);
end


out.rats = rats;
out.neuron = [in.cell];
out.flyID = [in.flyID];
out.name = name;
out.MeanOF_Cells = MeanOf_Cells;
out.StdOf_Cells = StdOf_Cells;
```

DSI, PD, ND calculation and polar plots script

Script name: Polarplots_PD_ND_DSI_quantification_elife

```
% This script plots Polar plots, PD, ND, and DSI quantification for  moving
% ON and OFF edges from manually selected ROIs.
% Input data must have been generated by moving_edge_analysis script.


% Authors: Miriam Henning and Sebastian Molina-Obando

close all
clear all
clc

%% USER INPUT: CONSTANTS

savingData = false; %"True" for saving data (variables and plots) in a folder in your computer.
Otherwise, write "false".
% Type the name of the folder
save_as= 'Df +';%'+ +', 'D +', 'Df +', 'D Df', '+ + 2.5uM PTX', '+ + 2.5uM PTX Control', '+ + 100uM
MPEP'
load_data_from =
'C:\MATLAB_FOLDER\2p\2p_pData\T4_T5_Layer_Data\T4_T5_LexA_LexAop_GCaMP6f_rec2
8\LumDecLumInc_8Dir_opt\Df +'; % Select where your data has been stored

% Choosing colors for every layer

COLOR_OF_PLOT_LPA = [0 .5 0]; % GREEN
COLOR_CLOUD_LPA =[.5 .7 .5];  % GREEN cloud

COLOR_OF_PLOT_LPB = [0 0 1];  %BLUE;
COLOR_CLOUD_LPB = [.5 .5 1];  %BLUE cloud

COLOR_OF_PLOT_LPC = [1 0 0];  %RED
COLOR_CLOUD_LPC = [1 .5 .5];  %RED cloud

COLOR_OF_PLOT_LPD = [.7 .7 0];%YELLOW
COLOR_CLOUD_LPD = [.9 .9 .5]; %YELLOW cloud

COLOR_OF_PLOT_M9 = [99/256 99/256 99/256]; %GREY
COLOR_CLOUD_M9 = [189/256 189/256 189/256]; %GREY cloud

COLOR_OF_PLOT_Lo = [99/256 99/256 99/256]; %GREY
COLOR_CLOUD_Lo = [189/256 189/256 189/256]; %GREY cloud

Color=[COLOR_OF_PLOT_LPA;COLOR_OF_PLOT_LPB;COLOR_OF_PLOT_LPC;COLOR_O
F_PLOT_LPD];

%% Data Analysis

% Loading Data and data organization
Condition = load([load_data_from,'\processed_Data_manualROIs.mat']);
Conditions={'Condition'};
LAYERS = ["LPA","LPB","LPC","LPD"]; % ["LPA","LPB","LPC","LPD","M9","Lo"];
["LPA","LPB","LPC","LPD"];
onedeg=2*pi/360; %in rad
theta=[90, 45, 0, 315, 270, 225, 180, 135, 90]; % For 8 directions
```

```
direction = theta;
theta=theta*onedeg;

%% Extract response peaks to every direction for the OFF and ON edge

NCon=2; %Number of Contrasts (here Only ON and OFF)

for NCon=1:length(Conditions)
    COND=eval(Conditions{NCon});

        LPA=COND.T4T5_LPA_agg.MeanOF_Cells;
        LPB=COND.T4T5_LPB_agg.MeanOF_Cells;
        LPC=COND.T4T5_LPC_agg.MeanOF_Cells;
        LPD=COND.T4T5_LPD_agg.MeanOF_Cells;

        %Plotting whole traces for each layer and all directions
        t= 0.1:0.1:size(COND.T4T5_LPA_agg.rats,3)/10;
        if  length(LAYERS)> 4
            M9=COND.T4T5_M9_agg.MeanOF_Cells;
            Lo=COND.T4T5_Lo_agg.MeanOF_Cells;
        end

        for L = 1: length(LAYERS)
            LAYER = char(LAYERS(L));
            cur_LAYER = eval(['COND.T4T5_',LAYER,'_agg.MeanOF_Cells']);
            COLOR_OF_PLOT = eval(['COLOR_OF_PLOT_',LAYER]);
            COLOR_CLOUD = eval(['COLOR_CLOUD_',LAYER]);
            figure(L)
            hold on
            for d = 1:8
                subplot(4,2,d)
                patch([0 4 4 0]',[-0.5 -0.5 45 45]',[0.85 0.85 0.85],'EdgeColor','none');
                set(gca, 'XLim', [0 8])
                set(gca, 'YLim', [-0.5 2.5])
                plot_err_patch_v2(t, squeeze(nanmean(cur_LAYER(:,d,:),1))',...
                (squeeze(nanstd(cur_LAYER(:,d,:),1)))/sqrt(size(cur_LAYER,1))...
                ,COLOR_OF_PLOT,COLOR_CLOUD);
                title(['T4&T5 NFlies = ', num2str(size(cur_LAYER,1)),' ', char(LAYER), ' Deg ',
int2str(direction(d))])

            end

        end

        F1 = figure(1);
        F2 = figure(2);
        F3 = figure(3);
        F4 = figure(4);

        if  length(LAYERS)> 4
            F5 = figure(5);
            F6 = figure(6);
        end

       %Before check if all Layers have same N(number of flies)
       N=unique([size(LPA,1), size(LPB,1), size(LPC,1), size(LPD,1)]);
       if N>1  %if one of the Layers was not selected in all Flies
           new=min(N);
```

```
        LPA=LPA(1:new,:,:);
        LPB=LPB(1:new,:,:);
        LPC=LPC(1:new,:,:);
        LPD=LPD(1:new,:,:);
      end


      OFF_A=max(LPA(:,:,1:40),[],3);
      ON_A=max(LPA(:,:,41:80),[],3);
      OFF_B=max(LPB(:,:,1:40),[],3);
      ON_B=max(LPB(:,:,41:80),[],3);
      OFF_C=max(LPC(:,:,1:40),[],3);
      ON_C=max(LPC(:,:,41:80),[],3);
      OFF_D=max(LPD(:,:,1:40),[],3);
      ON_D=max(LPD(:,:,41:80),[],3);

   % Polar Plot-responses to ON Edge
      F7= figure('Color', [1 1 1],'Position', [200 200 800 400]);
      subplot(1,2,1)
      P = polar(theta, 2 * ones(size(theta)));
      set(P, 'Visible', 'off')
      hold on

      for i=1:4 %four Layers

          LAYERS=['A','B','C','D'];

          tuningPdirPFly=eval(['ON_',LAYERS(i)])'; %tuning (strength of response) per direction
and per fly
          mtuningPdir=nanmean(tuningPdirPFly,2);
          stuningPdir=nanstd(tuningPdirPFly')/sqrt(length(tuningPdirPFly));
          rho=[mtuningPdir;mtuningPdir(1)]';
          PPp(i)=polar(theta,rho);
          PPp(i).Color=Color(i,:);
          hold on

          % Adding the error bars
          for ii=1:length(theta)-1

              e=stuningPdir(ii);
              m=mtuningPdir(ii);
              angle2=theta(ii);
              P=polar([angle2 angle2], [m-e, m+e]);
              P.Color=Color(i,:);

          end
      end
      [PPp.LineWidth] = deal(1.5)

      LegendName={'LayerA', 'LayerB', 'LayerC', 'LayerD'};
      set(gca,'YTickLabel','')
      set(gca,'FontSize',13)

      title([save_as, '( N= ', num2str(size(LPA,1)),') : Responses to ON Edge']);

      %For ON: DSI, PD and ND

      F10= figure('Color', [1 1 1],'Position', [200 200 800 400]);
```

```
        subplot(1,2,1)

        for i=1:4 %four Layers

            LAYERS=['A','B','C','D'];

            tuningPdirPFly=eval(['ON_',LAYERS(i)])'; %tuning (strength of response) per direction
and per fly

            % Calculation of direction selectivity index for ON
            tuningPdirPFly = tuningPdirPFly + 1;
            ON_Edge_PD_response_Fly = max(tuningPdirPFly,[],1);
            for r = 1: size(tuningPdirPFly,2)
                FlyVector = tuningPdirPFly(:,r);
                i_PD(r) = find(FlyVector == ON_Edge_PD_response_Fly(r));
                FlyVector2 = [FlyVector;FlyVector];
                i_ND(r) = i_PD(r) + 4;
                ON_Edge_ND_response_Fly(r) = FlyVector2 (i_ND(r));
            end
            ON_ND_response_Fly{i} = (ON_Edge_ND_response_Fly)-1; % Seb: substract the 1
previously added
            ON_PD_response_Fly{i} = (ON_Edge_PD_response_Fly) -1;% Seb: substract the 1
previously added
            ON_DSindexFly = (ON_Edge_PD_response_Fly -
ON_Edge_ND_response_Fly)./ON_Edge_PD_response_Fly;
            LAYER_ON_DSindexFly{i} = ON_DSindexFly;
            ON_DSindex_mean = mean(ON_DSindexFly)
            ON_DSindex_std = std(ON_DSindexFly);
            ON_DSindex_sem = ON_DSindex_std/sqrt(length(ON_DSindexFly))

            %BarPlot direction selective index (DSI) for ON
            hold on;
            b1= bar(i,ON_DSindex_mean,'FaceColor','flat');
            e = errorbar(i,ON_DSindex_mean,ON_DSindex_sem,'k.');
            b1.FaceColor = Color(i,:);
            e.LineWidth = 0.75;
            ylim([0 1]);

        end
            ylabel('DSI');
            title(['ON Edge 8D. LAYERS ', save_as]);

        % Amplitude Response PD BarPlot
        F11= figure('Color', [1 1 1],'Position', [200 200 800 400]);
        subplot(1,2,1)

        for i=1:4 %four Layers

            LAYERS=['A','B','C','D'];

            tuningPdirPFly=eval(['ON_',LAYERS(i)])'; %tuning (strength of response) per direction
and per fly

            ON_PD_mean = mean(ON_PD_response_Fly{i})
            ON_PD_std = std(ON_PD_response_Fly{i});
            ON_PD_sem = ON_PD_std/sqrt(length(ON_PD_response_Fly{i}))

            %BarPlot prefer direction (PD) for ON
```

```
        hold on;
        b2= bar(i,ON_PD_mean,'FaceColor','flat');
        e = errorbar(i,ON_PD_mean,ON_PD_sem,'k.');
        b2.FaceColor = Color(i,:);
        e.LineWidth = 0.75;
        ylim([0 3]);

    end
    ylabel('PD dF/F');
    title(['ON Edge 8D. LAYERS ', save_as]);

    % Amplitude Response ND BarPlot
    F12= figure('Color', [1 1 1],'Position', [200 200 800 400]);
    subplot(1,2,1)

    for i=1:4 %four Layers

        LAYERS=['A','B','C','D'];

        tuningPdirPFly=eval(['ON_',LAYERS(i)])'; %tuning (strength of response) per direction
and per fly

        ON_ND_mean = mean(ON_ND_response_Fly{i});
        ON_ND_std = std(ON_ND_response_Fly{i});
        ON_ND_sem = ON_ND_std/sqrt(length(ON_ND_response_Fly{i}));

        %BarPlot null direction (ND) for ON
        hold on;
        b2= bar(i,ON_ND_mean,'FaceColor','flat');
        e = errorbar(i,ON_ND_mean,ON_ND_sem,'k.');
        b2.FaceColor = Color(i,:);
        e.LineWidth = 0.75;
        ylim([0 3]);

    end
    ylabel('ND dF/F');
    title(['ON Edge 8D. LAYERS ', save_as]);

    % Polar Plot-responses to OFF Edge
    figure(F7)
    subplot(1,2,2)
    P = polar(theta, 2 * ones(size(theta)));
    set(P, 'Visible', 'off')
    hold on

    for i=1:4 %four Layers

        LAYERS=['A','B','C','D'];

        tuningPdirPFly=eval(['OFF_',LAYERS(i)])'; %tuning (strength of response) per direction
and per fly
        mtuningPdir=nanmean(tuningPdirPFly,2);
        stuningPdir=nanstd(tuningPdirPFly')/sqrt(length(tuningPdirPFly));
        rho=[mtuningPdir;mtuningPdir(1)]';
        PPp(i)=polar(theta,rho);
        PPp(i).Color=Color(i,:);
        hold on
        PolarPlot_std_new
```

```
    end
    [PPp.LineWidth] = deal(1.5)

    set(gca,'YTickLabel','')
    set(gca,'FontSize',13)

    title([Conditions{NCon}, '( N= ', num2str(size(LPA,1)),') : Responses to OFF Edge']);

    %For OFF: DSI, PD and ND
    figure(F10)
    subplot(1,2,2)

    for i=1:4 %four Layers

        LAYERS=['A','B','C','D'];

        % Calculation of direction selectivity index for OFF
        tuningPdirPFly=eval(['OFF_',LAYERS(i)])'; %tuning (strength of response) per direction
and per fly
        % Calculation of direction selectivity index for OFF
        tuningPdirPFly = tuningPdirPFly + 1; %Seb: adding 1
        OFF_Edge_PD_response_Fly = max(tuningPdirPFly,[],1);
        for r = 1: size(tuningPdirPFly,2)
            FlyVector = tuningPdirPFly(:,r);
            i_PD(r) = find(FlyVector == OFF_Edge_PD_response_Fly(r));
            FlyVector2 = [FlyVector;FlyVector];
            i_ND(r) = i_PD(r) + 4;
            OFF_Edge_ND_response_Fly(r) = FlyVector2 (i_ND(r));
        end
        OFF_ND_response_Fly{i} = (OFF_Edge_ND_response_Fly)- 1; % Seb: substract the 1
previously added
        OFF_PD_response_Fly{i} = (OFF_Edge_PD_response_Fly)- 1; % Seb: substract the 1
previously added
        OFF_DSindexFly = (OFF_Edge_PD_response_Fly -
OFF_Edge_ND_response_Fly)./OFF_Edge_PD_response_Fly;
        LAYER_OFF_DSindexFly{i} = OFF_DSindexFly;
        OFF_DSindex_mean = mean(OFF_DSindexFly)
        OFF_DSindex_std = std(OFF_DSindexFly);
        OFF_DSindex_sem = OFF_DSindex_std/sqrt(length(OFF_DSindexFly))

        %BarPlot direction selective index (DSI) for OFF
        hold on;
        b1= bar(i,OFF_DSindex_mean,'FaceColor','flat');
        e = errorbar(i,OFF_DSindex_mean,OFF_DSindex_sem,'k.');
        b1.FaceColor = Color(i,:);
        e.LineWidth = 0.75;
        ylim([0 1]);

    end
    ylabel('DSI');
    title(['OFF Edge 8D. LAYERS ', save_as]);

    % Amplitude Response ND BarPlot
    figure(F12)
    subplot(1,2,2)

    for i=1:4 %four Layers
```

```
            LAYERS=['A','B','C','D'];

            tuningPdirPFly=eval(['OFF_',LAYERS(i)])'; %tuning (strength of response) per direction
and per fly

            OFF_ND_mean = mean( OFF_ND_response_Fly{i});
            OFF_ND_std = std( OFF_ND_response_Fly{i});
            OFF_ND_sem = OFF_ND_std/sqrt(length(OFF_ND_response_Fly{i}));

            %BarPlot null direction (ND) for OFF
            hold on;
            b2= bar(i,OFF_ND_mean,'FaceColor','flat');
            e = errorbar(i,OFF_ND_mean,OFF_ND_sem,'k.');
            b2.FaceColor = Color(i,:);
            e.LineWidth = 0.75;
            ylim([0 3]);

        end
        ylabel('ND dF/F');
        title(['OFF Edge 8D. LAYERS ', save_as]);

        % Amplitude Response PD BarPlot
        figure(F11)
        subplot(1,2,2)

        for i=1:4 %four Layers

            LAYERS=['A','B','C','D'];

            tuningPdirPFly=eval(['OFF_',LAYERS(i)])'; %tuning (strength of response) per direction
and per fly

            OFF_PD_mean = mean(OFF_PD_response_Fly{i})
            OFF_PD_std = std(OFF_PD_response_Fly{i});
            OFF_PD_sem = OFF_PD_std/sqrt(length(OFF_PD_response_Fly{i}))

            %BarPlot prefer direction (PD) for OFF
            hold on;
            b2= bar(i,OFF_PD_mean,'FaceColor','flat');
            e = errorbar(i,OFF_PD_mean,OFF_PD_sem,'k.');
            b2.FaceColor = Color(i,:);
            e.LineWidth = 0.75;
            ylim([0 3]);

        end
        ylabel('PD dF/F');
        title(['OFF Edge 8D. LAYERS ', save_as]);

end

%% Saving Data
if savingData

    saveas(F1,
['C:\MATLAB_FOLDER\2p\2p_pData\T4_T5_Layer_Data\T4_T5_LexA_LexAop_GCaMP6f_rec
28\LumDecLumInc_8Dir_opt\', save_as, '\LPA_traces.pdf']);
```

```
    saveas(F2,
['C:\MATLAB_FOLDER\2p\2p_pData\T4_T5_Layer_Data\T4_T5_LexA_LexAop_GCaMP6f_rec
28\LumDecLumInc_8Dir_opt\', save_as, '\LPB_traces.pdf']);
    saveas(F3,
['C:\MATLAB_FOLDER\2p\2p_pData\T4_T5_Layer_Data\T4_T5_LexA_LexAop_GCaMP6f_rec
28\LumDecLumInc_8Dir_opt\', save_as, '\LPC_traces.pdf']);
    saveas(F4,
['C:\MATLAB_FOLDER\2p\2p_pData\T4_T5_Layer_Data\T4_T5_LexA_LexAop_GCaMP6f_rec
28\LumDecLumInc_8Dir_opt\', save_as, '\LPD_traces.pdf']);
    saveas(F5,
['C:\MATLAB_FOLDER\2p\2p_pData\T4_T5_Layer_Data\T4_T5_LexA_LexAop_GCaMP6f_rec
28\LumDecLumInc_8Dir_opt\', save_as, '\M9_traces.pdf']);
    saveas(F6,
['C:\MATLAB_FOLDER\2p\2p_pData\T4_T5_Layer_Data\T4_T5_LexA_LexAop_GCaMP6f_rec
28\LumDecLumInc_8Dir_opt\', save_as, '\Lo_traces.pdf']);


    saveas(F7,
['C:\MATLAB_FOLDER\2p\2p_pData\T4_T5_Layer_Data\T4_T5_LexA_LexAop_GCaMP6f_rec
28\LumDecLumInc_8Dir_opt\', save_as, '\PolarPlot_8D.pdf']);
    saveas(F10,
['C:\MATLAB_FOLDER\2p\2p_pData\T4_T5_Layer_Data\T4_T5_LexA_LexAop_GCaMP6f_rec
28\LumDecLumInc_8Dir_opt\', save_as, '\DSI_8D_Fly_LAYER.pdf']);
    saveas(F11,
['C:\MATLAB_FOLDER\2p\2p_pData\T4_T5_Layer_Data\T4_T5_LexA_LexAop_GCaMP6f_rec
28\LumDecLumInc_8Dir_opt\', save_as, '\PD_Response.pdf']);
    saveas(F12,
['C:\MATLAB_FOLDER\2p\2p_pData\T4_T5_Layer_Data\T4_T5_LexA_LexAop_GCaMP6f_rec
28\LumDecLumInc_8Dir_opt\', save_as, '\ND_Response.pdf']);


save(['C:\MATLAB_FOLDER\2p\2p_pData\T4_T5_Layer_Data\T4_T5_LexA_LexAop_GCaMP6
f_rec28\LumDecLumInc_8Dir_opt\', save_as,
'\PD_Response_and_DSI_LAYER'],'LAYER_OFF_DSindexFly','LAYER_ON_DSindexFly','ON_
PD_response_Fly','OFF_PD_response_Fly','ON_ND_response_Fly','OFF_ND_response_Fly');
end
```
